# Emerging compound air–soil heatwaves amplify global vegetation losses

**DOI:** 10.1093/nsr/nwag603

**Published:** 2026-09-17

**Authors:** Sijia Wu, Ming Luo, R Iestyn Woolway, Zhen Liu, Xing Li, Haicheng Zhang, Weilin Liao, Xiaoyu Wang, Manqing Shi, Qiuting Wang, Tao Pei, Xiaoping Liu

**Affiliations:** Guangdong Provincial Key Laboratory of Urbanization and Geo-Simulation, School of Geography and Planning, Sun Yat-sen University, Guangzhou 510006, China; Guangdong Provincial Key Laboratory of Urbanization and Geo-Simulation, School of Geography and Planning, Sun Yat-sen University, Guangzhou 510006, China; School of Ocean Sciences, Bangor University, Menai Bridge LL59 5AB, UK; Earth, Ocean and Atmospheric Sciences (EOAS) Thrust, Function Hub, The Hong Kong University of Science and Technology (Guangzhou), Guangzhou 511453, China; Guangdong Provincial Key Laboratory of Urbanization and Geo-Simulation, School of Geography and Planning, Sun Yat-sen University, Guangzhou 510006, China; Guangdong Provincial Key Laboratory of Urbanization and Geo-Simulation, School of Geography and Planning, Sun Yat-sen University, Guangzhou 510006, China; Guangdong Provincial Key Laboratory of Urbanization and Geo-Simulation, School of Geography and Planning, Sun Yat-sen University, Guangzhou 510006, China; Guangdong Provincial Key Laboratory of Urbanization and Geo-Simulation, School of Geography and Planning, Sun Yat-sen University, Guangzhou 510006, China; Guangdong Provincial Key Laboratory of Urbanization and Geo-Simulation, School of Geography and Planning, Sun Yat-sen University, Guangzhou 510006, China; Guangdong Provincial Key Laboratory of Urbanization and Geo-Simulation, School of Geography and Planning, Sun Yat-sen University, Guangzhou 510006, China; State Key Laboratory of Resources and Environmental Information System, Institute of Geographic Sciences and Natural Resources Research, Chinese Academy of Sciences, Beijing 100101, China; University of Chinese Academy of Sciences, Beijing 100049, China; Jiangsu Center for Collaborative Innovation in Geographical Information Resource Development and Application, Nanjing 210023, China; Guangdong Provincial Key Laboratory of Urbanization and Geo-Simulation, School of Geography and Planning, Sun Yat-sen University, Guangzhou 510006, China

**Keywords:** compound air–soil heatwaves, soil heatwaves, gross primary productivity (GPP), climate change, ecological impacts

## Abstract

Compound air–soil heatwaves—periods in which extreme air and soil temperatures co-occur—pose a growing threat to terrestrial ecosystems, yet their occurrence and impacts remain poorly understood. Here, we show that compound air–soil heatwaves have become more frequent (0.59 ± 0.07 events decade^−1^), longer-lasting (0.18 ± 0.04 days decade^−1^) and more intense (4.02 ± 0.77°C × days decade^−1^) since 1979. These rates of change far exceed those for independent air and soil heatwaves and are driving increasingly widespread reductions in vegetation productivity. On average, ∼98.5 million km^2^ of land experienced gross primary productivity (GPP) losses during compound air–soil heatwaves. Globally, annual GPP losses have risen by 0.61 gC m^−2^ year⁻^1^ decade⁻^1^, which is a 30.5- and 12.2-fold increase relative to those of independent air and soil heatwaves, respectively. Climate model projections indicate that compound air–soil heatwaves will continue to intensify and expand spatially under future warming, particularly under the high-emission scenario. Given pronounced GPP reductions during compound heatwaves, their projected intensification is likely to pose an increasing threat to terrestrial ecosystem productivity. Our findings highlight the escalating ecological impacts of compound heatwaves and underscore the urgent need for adaptation and preparedness strategies to address this compound hazard in a warming world.

## Teaser

Compound air-soil heatwaves are becoming more frequent, longer-lasting, and more intense worldwide, with increasing potential impacts on global vegetation productivity.

## INTRODUCTION

Heatwaves are among the most damaging extreme climate events, with profound consequences for ecosystems, agriculture and global carbon cycle [1–3]. As the climate warms, atmospheric heatwaves are becoming more frequent, longer lasting and more intense across most land regions [4–6]. At the same time, warming is propagating below the land surface, driving substantial increases in soil temperatures and heat extremes [7–12]. Yet research and risk assessments have largely treated heatwaves as an atmospheric phenomenon, despite many biological and hydrological processes being strongly influenced by temperatures below ground [12–16].

Soil temperature plays a central role in regulating plant growth, microbial activity and land–atmosphere exchanges of water and carbon [17–20]. Importantly, plants respond simultaneously to conditions above and below the surface [21,22]. When periods of hot air coincide with those of hot soils, vegetation experiences a unique combination of stresses that cannot be inferred from either component alone. Specifically, hot and typically dry air increases atmospheric evaporative demand, rapidly drawing water from leaves, while hot soils impair root water uptake and accelerate root respiration [23]. This combination can force plants to close their stomata, shutting down transpiration and photosynthesis while increasing leaf temperatures beyond ambient air temperature [24,25]. Such conditions can trigger hydraulic failure, carbon starvation and widespread productivity losses [21]. Despite these strong mechanistic links, the co-occurrence of atmospheric and soil heat extremes remains poorly understood.

Isolated examples suggest that periods of concurrent hot air and soil can have severe consequences. During the 2003 European heatwave, widespread hot and dry conditions led to extensive crop failures, forest mortality and ∼30% reduction in gross primary productivity (GPP) [3,26] over Europe. More recently, record-breaking temperatures in 2023 highlighted the increasing likelihood that extreme heat simultaneously affects both the land surface and the atmosphere. Previous work has shown that soil temperatures alone can explain a large fraction of ecosystem productivity variability, even exceeding the influence of near-surface air temperature [17]. However, the frequency, evolution and ecological impacts of compound air–soil heatwaves globally are largely unknown. It is unclear how often these events occur, whether they are changing faster than independent air or soil heatwaves, and how strongly they affect terrestrial carbon uptake.

Here, we present a global assessment of compound air–soil heatwaves and their impacts on vegetation productivity from 1979 to 2024, with future projections until the end of the 21st century. Using multiple reanalysis datasets and Coupled Model Intercomparison Project Phase 6 (CMIP6) climate simulations, we quantify how the frequency, duration, intensity and spatial extent of compound air–soil heatwaves compare with independent air and soil heat extremes. We then investigate the local heating processes associated with these events through a surface energy budget diagnostic analysis and assess their ecological consequences by quantifying the associated changes in GPP. Our results suggest that compound air–soil heatwaves are emerging as a rapidly intensifying climate hazard with disproportionate impacts on the terrestrial carbon sink, underscoring the need to explicitly consider coupled above- and below-ground heat extremes in climate risk assessments.

## RESULTS

### Compound air–soil heatwaves are widespread and linked to land–atmosphere coupling

We define compound air–soil heatwaves as periods when daily near-surface air temperature at 2 m above the land surface and daily mean soil temperature in the upper 0–10 cm below the land surface simultaneously exceed their local, seasonally varying 90th percentile thresholds for at least three consecutive days during the extended summer season (Materials and Methods and Text S1). This shallow soil layer (0–10 cm) is ecologically relevant, as the temperature sensitivity of root respiration in shallow roots is often higher than that in deep roots [27]. Historically (1979–2024), global land areas experienced an average of 1.73 compound air–soil heatwave events per year, substantially more frequent than independent air (0.19 events year⁻^1^) or soil (0.32 events year⁻^1^) heatwaves (Fig. 1; Figs S1 and S2). Compound events occur most frequently in tropical and arid regions, with pronounced hotspots across sub-Saharan Africa, southern North America, northern South America and South and Southeast Asia (Fig. 1a). Compound air–soil heatwaves are also more persistent and intense than independent events (Fig. 1b–d). On average, compound air–soil heatwaves last 4.54 days, compared with 3.34 and 3.56 days for independent air and soil heatwaves, respectively, and exhibit higher peak temperature anomalies (2.53°C) and cumulative intensity (18.12°C × days). These patterns are robust across reanalysis products, alternative percentile thresholds, temperature metrics, spatial resolutions and seasons (Figs S3–>S16).

**Figure 1.**
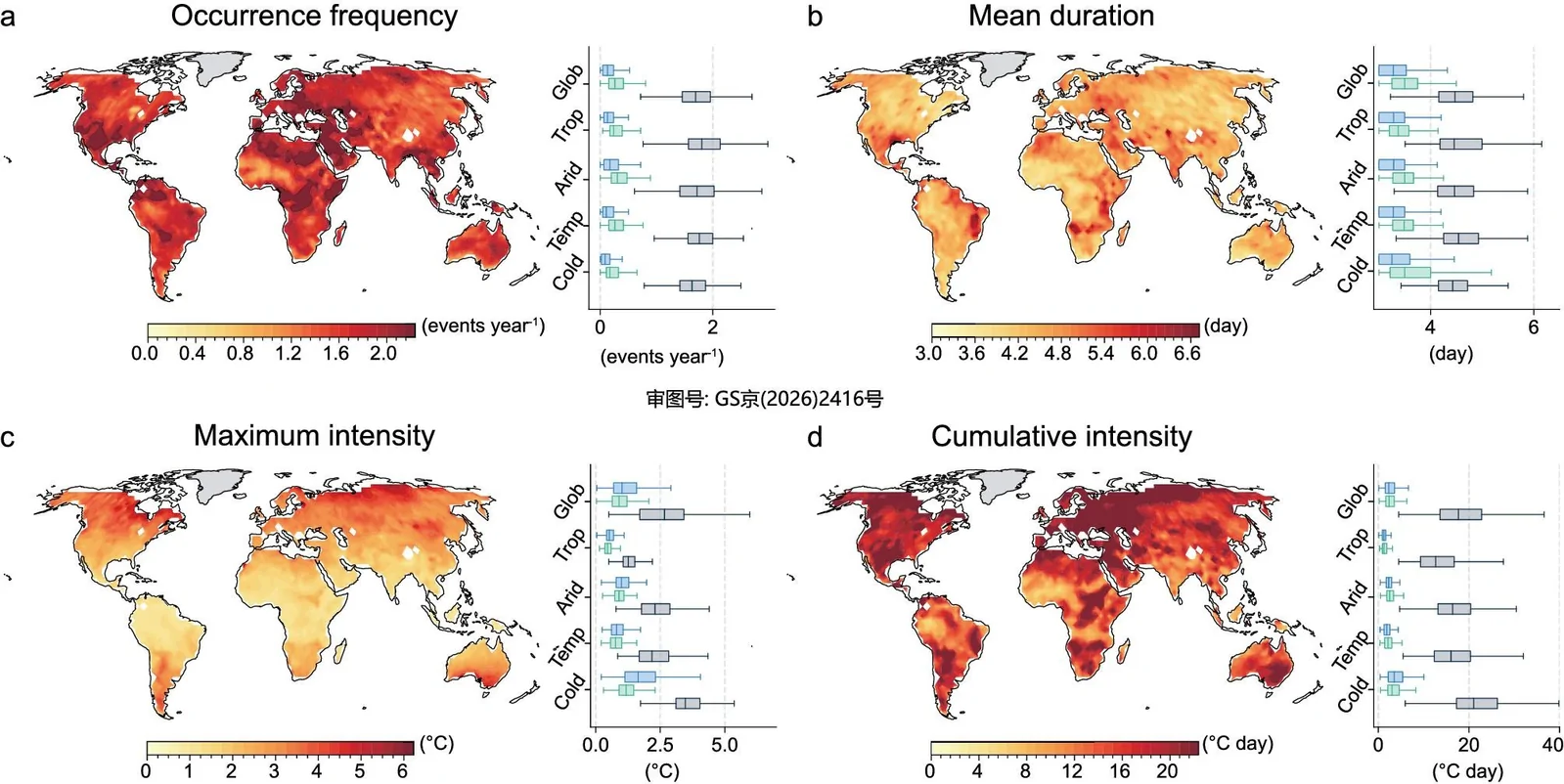
Observed climatology of compound air–soil heatwaves. (a–d) Spatial distribution of the climatological mean of annual mean occurrence frequency (a), mean duration (b), maximum intensity (c) and cumulative intensity (d) of compound air–soil heatwaves over global land areas from 1979 to 2024. The box plots charts accompanying the maps showing the distributions of climatological mean of the corresponding metrics of independent air heatwaves (blue), independent soil heatwaves (green) and compound air–soil heatwaves (gray) averaged over global land areas and across different climate zones (Glob, global; Trop, tropical zone; Arid, arid zone; Temp, temperate zone; Cold, cold zone). The centerline indicates the median value, the box bounds depict the 25th and 75th percentile values and the whiskers indicate the lower and upper quartiles ±1.5 times the interquartile range of the values.

Although the mechanisms underlying independent air heatwaves have been extensively studied [28–30], the local energetic processes accompanying compound air–soil heatwaves remain poorly understood, and how these processes differ from independent air or soil heatwave events is yet to be examined. We therefore diagnose the energetic processes contributing to near-surface air and shallow soil warming using a surface energy budget decomposition framework (Materials and Methods, Texts S2 and S3 and Figs S17–>S22). Near-surface air temperature anomalies are decomposed into contributions from advection heating, adiabatic heating and diabatic heating, whereas shallow soil temperature anomalies are partitioned into contributions from surface radiative fluxes, turbulent heat fluxes and conductive heat exchange with deeper soil layers (Texts S2 and S3). Compound air–soil heatwaves feature simultaneous and amplified warming of both near-surface air (+4.3°C) and shallow soils (+3.7°C), exceeding the anomalies during independent events. For near-surface air temperature, the largest positive contribution comes from diabatic heating, mainly associated with enhanced shortwave and longwave radiations (Figs S18, S20–>S22). Similar radiative processes contribute to soil warming and are partly offset by turbulent and subsurface heat fluxes (Figs S19–>S22).

We conduct a composite analysis to characterize the hydroclimatic conditions associated with each type of heatwave events [31,32] (Text S4). The composite mean patterns reveal distinct atmospheric and land-surface conditions among the three heatwave types, with compound air–soil heatwave events coincide with reduced cloud cover, depleted soil moisture and strongly elevated vapor pressure deficit (VPD; Fig. S23). In brief, reduced cloudiness enhances radiative heating, while high VPD increases atmospheric evaporative demand. At the same time, soil moisture depletion limits evaporative cooling, shifting surface energy partitioning toward sensible heating and subsurface heat storage [33]. These anomalies characterize a tightly coupled land–atmosphere state associated with concurrent warming of the atmosphere and soil, particularly in moisture-limited regions.

The temporal evolution of composite anomalies further reveals distinct energy partitioning processes and hydroclimatic conditions among heatwave types (Fig. S24). For independent air heatwaves, rapid atmospheric warming is accompanied by enhanced radiation, but relatively sufficient antecedent soil moisture sustains evaporative cooling, which helps modulate soil temperatures. Conversely, independent soil heatwaves are characterized by pre-existing soil moisture deficits that restrict evaporative cooling, channeling radiation largely into soil heating, while atmospheric heating stays moderate. Compound air–soil heatwaves combine and amplify these features through a consistent set of reinforcing feedbacks. Additional ecoregion and aridity analyses show that air–soil temperature coupling and compound event frequency vary among ecosystem types (Fig. S25). Notably, the annual frequency of compound events is positively correlated with local air–soil coupling strength (*ρ * = 0.386, *P* < 0.001). These results indicate that the observed hotspots in arid or sparsely vegetated regions partly reflect pre-existing land-surface conditions that favor co-occurring above- and below-ground heat extremes.

### Compound air–soil heatwaves suppress global vegetation productivity

We quantify GPP losses during independent air, independent soil and compound air–soil heatwaves (Fig. 2; see Materials and Methods). All heatwave types reduce ecosystem productivity, but compound heatwaves produce the strongest impacts. Globally, independent soil and air heatwaves reduce GPP by 0.156 and 0.319 gC m^−2^ day⁻^1^, respectively, whereas compound air–soil heatwaves cause a substantially larger mean reduction of 0.491 gC m^−2^ day⁻^1^ and a broader range of negative responses (Fig. 2). This suggests that concurrent above- and below-ground heat stress is associated with stronger ecosystem impacts than either heatwave type alone. After matching independent and compound heatwaves within comparable temperature exceedance and duration categories, compound events still show larger GPP reductions than independent heatwaves across most regions, indicating that this stronger response is not solely explained by differences in event intensity or duration (Fig. S26). However, the factorial interaction analysis shows that the global response is approximately additive rather than synergistic (Text S5). The global interaction effect is small and statistically insignificant (0.006 gC m^−2^ day⁻^1^), reflecting an approximately additive global mean response. Regionally, temperate ecosystems show a negative departure from additivity (−0.103 gC m^−2^ day^−1^, *P* = 0.001), whereas arid ecosystems show a sub-additive response (0.028 g C m^−2^ day^−1^, *P* = 0.007).

**Figure 2.**
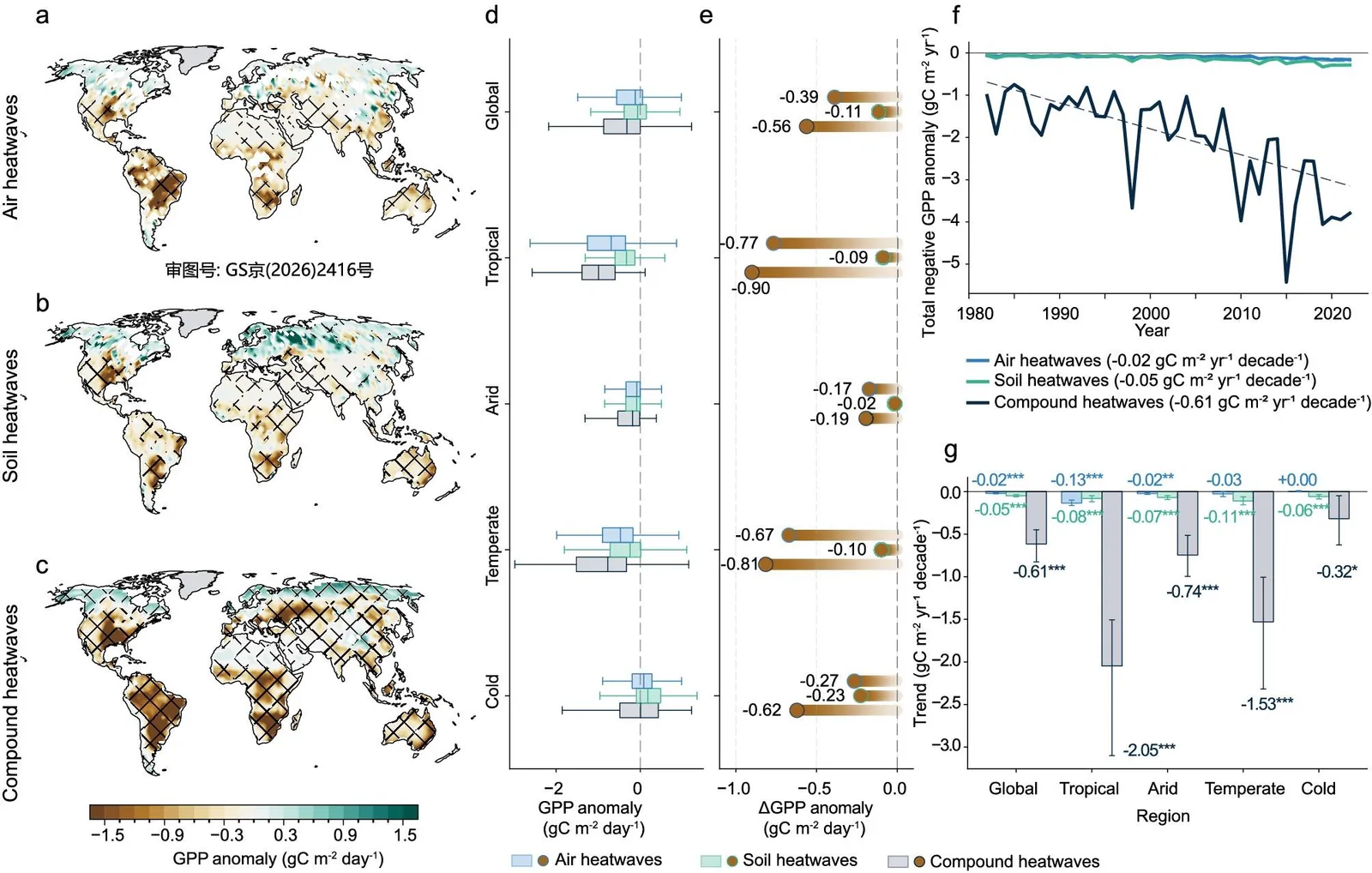
Ecological impacts of independent and compound air–soil heatwaves. (a–c) Spatial distribution of the composite GPP anomalies during independent air heatwaves (a), independent soil heatwaves (b) and compound air–soil heatwaves (c) over global land areas. The GPP product is derived from the BESS v2.0 dataset (1982–2022) [71]. Regions where the composite mean passes the 0.1 significance level by the Student’s *t*-test are marked by cross-hatching. (d) Boxplots showing the distribution of regional composite GPP anomalies during independent air heatwaves (blue), independent soil heatwaves (green) and compound air–soil heatwaves (gray) over global land areas and across different climate zones. (e) Difference between the minimum composite anomalies of GPP during the heatwave event and the GPP anomaly on 1 day before the onset over global land areas and across different climate zones. Negative values (brown) indicate a reduction in GPP triggered by the heatwave relative to the pre-event state. (f and g) Changes in ecological impacts of independent and compound air–soil heatwaves. (f) Time series of the spatially averaged annual GPP loss, defined as the sum of negative GPP anomalies on heatwave days from 1982 to 2022. Dashed lines indicate the corresponding linear trends that pass the 0.05 significance level based on the Theil–Sen estimator. Slope estimates for the trend per decade are given in parentheses. (g) Decadal trends of the annual GPP loss for three types of heatwaves over global land areas and across different climate zones. The error bars indicate the 95% bootstrap confidence interval of the trends. *P < 0.05 for long-term trend; **P < 0.01; ***P < 0.001.

Compound heatwaves also affect a much larger fraction of vegetated land. Significant negative GPP anomalies occur across 40.7% and 38.0% of vegetated grids during independent air and soil heatwaves, respectively, but expand to 69.7% of vegetated grid cells (∼98.5 million km^2^) during compound air–soil heatwave events (Fig. 2a–c). Spatially, the strongest GPP reductions during compound air–soil heatwaves are concentrated in the central USA, eastern Brazil, southern Africa and forested regions of Australia, highlighting these regions as particularly vulnerable to compound air–soil heatwaves.

Ecosystem sensitivity varies across climate zones (Fig. 2d). Tropical and temperate regions show the strongest productivity reductions during compound air–soil heatwaves, whereas cold regions exhibit positive GPP responses to independent soil heatwaves. Moderate soil warming in cold regions can enhance root activity and nutrient availability by relaxing root-zone temperature limitation, whereas this positive effect may be offset when warming is accompanied by enhanced respiration, soil moisture loss and increased atmospheric evaporative demand [34–36]. This suggests that the alleviation of low temperature limitation may outweigh concurrent moisture and atmospheric constraints under these extreme events in cold regions [37]. Rapid vegetation responses during these events further reveal the severity of compound heat stress: compound air–soil heatwaves produce the steepest immediate GPP reductions following event onset, particularly in tropical and temperate ecosystems (Fig. 2e and Fig. S27).

These short-term physiological responses accumulate into substantial long-term productivity losses. Here, the annual GPP loss is calculated at grid level as the annual sum of negative daily GPP anomalies over all heatwave days [38], averaged across all valid vegetated grid cells within each region (Text S5). During 1982–2022, the global annual GPP loss associated with compound air–soil heatwaves strengthened significantly (0.61 gC m^−2^ year⁻^1^ decade⁻^1^), which is larger than trends for independent soil heatwaves (Fig. 2f and g; Text S5). The strongest increases in productivity losses occur in tropical and temperate regions, demonstrating that compound air–soil heatwaves are becoming increasingly associated with weakened terrestrial carbon uptake.

To examine the spatial heterogeneity of GPP responses, we fit random forest models [39] separately for each heatwave type using a common set of hydroclimatic and surface energy predictors (Materials and Methods and Text S6). This analysis identifies the environmental conditions associated with GPP reductions across grid cells. The models are trained on 80% of the grid cells and evaluate using the remaining 20% and out-of-bag validation, while predictor contributions are interpreted using the SHAP (SHapley Additive exPlanations) [40] values. Testing *R*^2^ values are 0.522, 0.575 and 0.709 for independent air, independent soil and compound air–soil heatwaves, respectively (Table S1). During compound air–soil heatwaves, the sensible heat flux contribution to soil temperature ranks the highest, followed by VPD and the shortwave radiation contribution to air temperature (Materials and Methods and Text S6; Figs S28–>S31). Increasing VPD is associated with stronger predicted GPP reductions. In contrast, soil moisture ranks the highest during independent air heatwaves, with drier conditions associated with more negative model predicted GPP anomalies. During independent soil heatwaves, the highest-ranked predictors are primarily turbulent heat flux contributions to air and soil temperatures.

### Compound air–soil heatwaves have increased rapidly since 1979

All three heatwave types have become more frequent since 1979, and compound air–soil heatwaves are increasing much faster than independent air or soil heatwaves (Fig. 3a and Fig. S32a). The global mean occurrence frequency of compound air–soil heatwaves has risen by 0.59 ± 0.07 events decade⁻^1^ (3.05 ± 0.46 total days decade⁻^1^; ‘±’ denotes the standard deviation of the bootstrapped Theil–Sen slope estimates), compared with 0.05 ± 0.004 events decade⁻^1^ (0.17 ± 0.02 total days decade⁻^1^) and 0.12 ± 0.02 events decade⁻^1^ (0.46 ± 0.06 total days decade^−1^) for independent air and soil heatwaves, respectively. This indicates a rapidly growing prevalence of compounding heat hazards over recent decades. To examine what underlies the rapid increase in compound heatwave frequency, we further decompose the historical trend into contributions from changes in mean temperature, temperature variability and air–soil temperature dependence [41,42] (Figs S33 and S34). The increase in compound heatwave frequency is primarily associated with upward shifts in mean near-surface air and soil temperatures, whereas changes in temperature variability and air–soil temperature dependence make smaller contributions. This result is consistent with previous evidence that marine heatwave trends are primarily associated with rising mean sea surface temperatures rather than changes in temperature variability [43].

**Figure 3.**
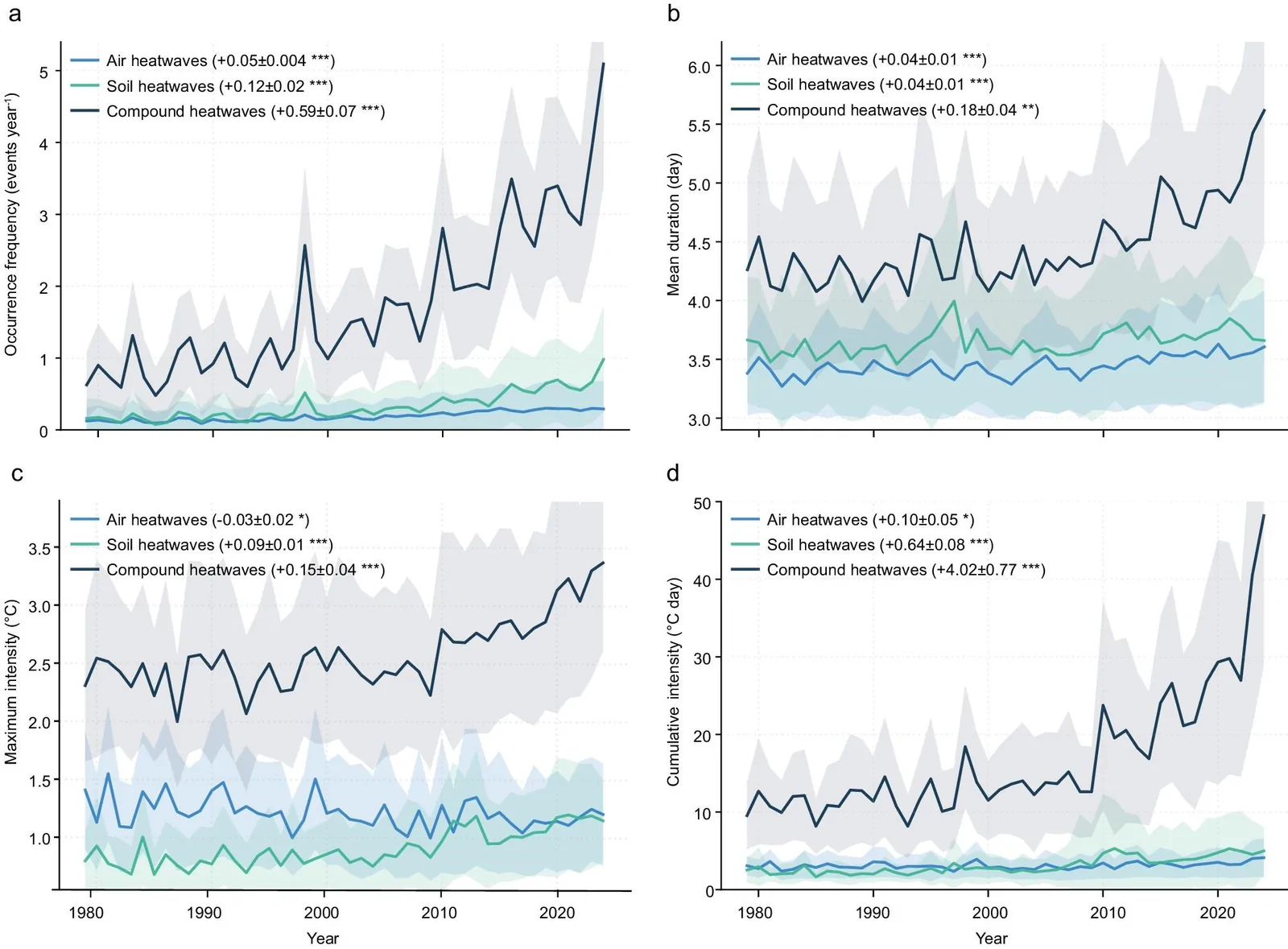
Long-term changes in independent and compound air–soil heatwaves. (a–d) Time series of the spatially averaged annual mean of occurrence frequency (a), mean duration (b), maximum intensity (c) and cumulative intensity (d) of independent air heatwaves (blue), independent soil heatwaves (green) and compound air–soil heatwaves (gray) over global land areas from 1979 to 2024. Solid line represents the time series of regional mean and the shaded regions represent half of the standard deviation. Slope estimates for the trend per decade are given in parentheses. *P < 0.05 for long-term trend; **P < 0.01; ***P < 0.001.

Globally, the duration, maximum intensity and cumulative intensity of compound air–soil heatwaves have increased by 0.18 ± 0.04 days decade⁻^1^, 0.15 ± 0.04°C decade⁻^1^ and 4.02 ± 0.77°C × days decade⁻^1^, respectively (Fig. 3b–d). Comparisons between earlier (1981–2010) and recent (2011–2024) periods and distributional shifts further confirm this rapid intensification (Fig. S35; Text S7). The increase in compound air–soil heatwaves is spatially widespread, with significant upward trends across most global land areas (Fig. 4). The strongest increases occur in tropical and arid regions, including western North America, Central and northern South America, western and southeastern Eurasia and much of Africa. In addition to becoming more frequent, compound air–soil heatwaves are expanding in spatial extent (Fig. S32c and e). The annual maximum daily affected area has increased by 3.01 ± 0.44 million km^2^ per decade, far exceeding trends for independent air (0.17 ± 0.03 million km^2^ decade⁻^1^) and soil heatwaves (0.48 ± 0.05 million km^2^ decade⁻^1^). Similarly, the annual mean daily affected area has increased by 1.28 ± 0.20 million km^2^ decade⁻^1^, compared with 0.07 ± 0.01 and 0.19 ± 0.02 million km^2^ decade⁻^1^ for independent air and soil heatwaves, respectively.

**Figure 4.**
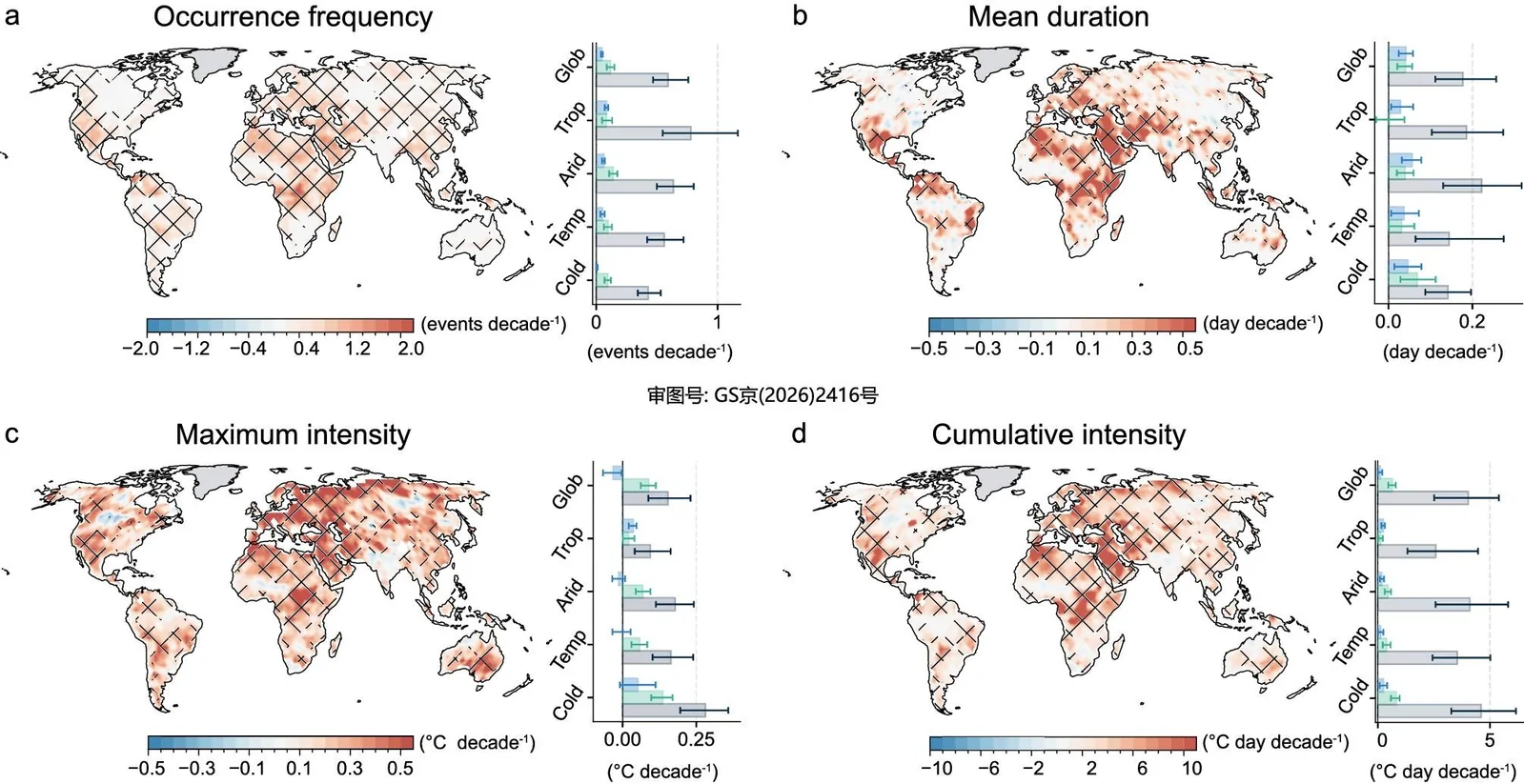
Observed historical trends of compound air–soil heatwaves. (a–d) Spatial distribution of long-term trends in the occurrence frequency (a), mean duration (b), maximum intensity (c) and cumulative intensity (d) of compound air–soil heatwaves over global land areas from 1979 to 2024. The trends passing the 0.1 significance level based on the modified nonparametric Mann–Kendall test [75] are marked by cross-hatching. The bar charts accompanying the maps showing the decadal trends of the corresponding metrics of independent air heatwaves (blue), independent soil heatwaves (green) and compound air–soil heatwaves (gray) averaged over global land areas and across different climate zones (Glob, global; Trop, tropical zone; Arid, arid zone; Temp, temperate zone; Cold, cold zone). Error bars indicate the 95% confidence interval of the trends.

### Future warming is projected to intensify more compound air–soil heatwaves

We project continued intensification of compound and independent heatwaves throughout the 21st century by CMIP6 simulations, which capture the observed climatology and historical trends (Fig. 5 and Fig. S32). Across all scenarios, compound air–soil heatwaves show the largest increases in frequency, duration, intensity and spatial extent, reinforcing the growing dominance of compounding extremes under continued warming. Under a high-emission scenario (SSP5-8.5), compound air–soil heatwaves intensify dramatically by the end of the century (2081–2100) relative to the historical baseline (1981–2010). Global mean frequency is projected to increase by 1678.6% ± 1179.8% (‘±’ denotes half of the inter-model standard deviation), accompanied by large increases in mean duration (616.2% ± 144.3%), maximum intensity (222.8% ± 30.6%) and cumulative intensity (3603.7% ± 1160.4%) (Fig. 5a–d). The spatial footprint of compound air–soil heatwaves expands sharply, with the annual mean affected area rising from 2.19 ± 0.56 to 40.91 ± 3.04 million km^2^ (Fig. S32f). Projected changes are substantially smaller under the intermediate-emission scenario SSP2-4.5, highlighting a strong dependence of future compound air–soil heatwave risk on greenhouse gas mitigation (Fig. 5 and Fig. S32).

**Figure 5.**
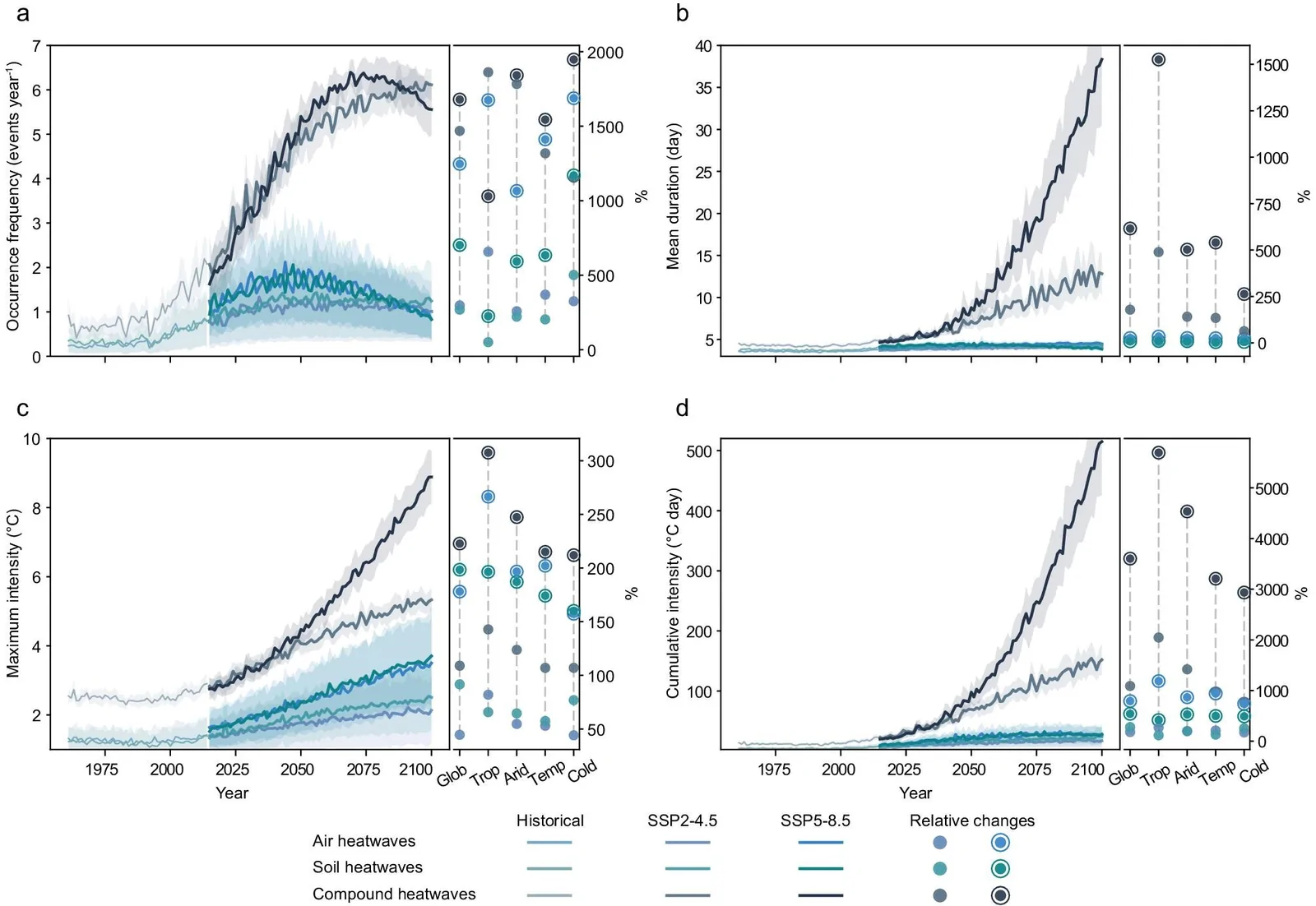
Projected future changes in independent and compound air–soil heatwaves. (a–d) Time series of spatially averaged annual mean of occurrence frequency (a), mean duration (b), maximum intensity (c) and cumulative intensity (d) of independent air heatwaves (blue), independent soil heatwaves (green) and compound air–soil heatwaves (gray) over global land areas during 1961–2100. Solid lines denote the multi-model ensemble means under the historical period (1961–2014) and future SSP2-4.5 and SSP5-8.5 (2015–2100) scenarios. Shading indicates the uncertainty, measured as half of the inter-model standard deviation. The embedded droplet plot showing the projected percentage changes in the corresponding metrics during the far future period (2081–2100) relative to the historical baseline (1981–2010) over global land areas and across different climate zones (Glob, global; Trop, tropical zone; Arid, arid zone; Temp, temperate zone; Cold, cold zone). Solid dots represent the SSP2-4.5 scenario and concentric circles represent the SSP5-8.5 scenario.

Given the larger historical GPP reductions during compound air–soil heatwaves (Fig. 2f and g), the projected intensification and spatial expansion suggest a growing potential threat to vegetation productivity under future climate warming. However, because daily GPP projections required to match individual heatwave events are highly limited across the CMIP6 multi-model ensemble, preventing a robust assessment, we do not directly quantify future GPP losses in our current study. Future work combining high-frequency climate projections with ecosystem carbon cycle simulations will be needed to assess how the productivity impacts of compound air–soil heatwaves evolve under climate warming. Such assessments should also account for biogeochemical and biophysical land climate feedbacks, as contrasting vegetation and land-use pathways can produce markedly different carbon storage and regional climate responses even under comparable radiative forcing [44].

## DISCUSSION AND IMPLICATIONS

Heatwave risk has traditionally been framed as an atmospheric phenomenon [4,5,29,45]. Our results show that this view is somewhat incomplete: compound air–soil heatwaves are emerging as the dominant form of terrestrial heat extreme, increasing faster than independent air or soil heatwaves and exerting stronger impacts on vegetation productivity. By explicitly linking above- and below-ground temperature extremes, our findings highlight a previously underappreciated climate hazard that poses a potential risk to the terrestrial carbon sink. The rapid intensification and spatial expansion of compound air–soil heatwaves have important ecological and societal implications. Longer-lasting hot and dry conditions can accelerate soil and vegetation drying, extending periods of flammable conditions and increasing wildfire risk [46]. These changes suggest that compound air–soil heatwaves represent a growing multi-sector climate risk.

We note that our compound framework does not assume that air and soil temperatures are physically independent; instead, it characterizes a compound heat state within a coupled land–atmosphere system. Although both air and soil temperatures may respond to common radiative and surface forcing, their responses are not identical [9]. Near-surface air temperature is influenced by atmospheric transport, turbulent mixing and boundary layer processes, whereas shallow soil temperature is additionally modulated by soil moisture, vegetation cover, soil thermal properties and subsurface heat storage [7,47]. Compound air–soil heatwaves capture ecosystem thermal stress that is not fully characterized by air or soil temperature alone. This joint perspective is consistent with the broader compound event framework, which emphasizes risks arising from the co-occurrence of multiple climate drivers and hazards [48]. Our sensitivity analyses within comparable temperature exceedance bins suggest that the longer duration and broader extent of compound events are not solely due to event definition (Fig. S36).

Our results further demonstrate that compound air–soil heatwaves suppress ecosystem productivity more strongly than independent heatwaves. Importantly, these GPP reductions cannot be attributed to heat alone, as heatwave events are often accompanied by atmospheric and soil dryness. This larger response reflects the simultaneous intensification of atmospheric heat, subsurface warming and atmospheric moisture demand and soil moisture depletion. Soil moisture depletion contributes to this response by limiting root water uptake and reducing evaporative cooling. The magnitude of this water limitation may vary among ecosystems because effective rooting depth regulates plant access to subsurface water and the sensitivity of transpiration to soil moisture deficits [49]. Elevated VPD in the overlying atmosphere further increases evaporative demand and promotes stomatal closure, rapidly constraining photosynthesis [50,51]. Considering that soil moisture, heat fluxes, evaporation and VPD are tightly coupled during heatwaves, we interpret these variables as linked components of a hydroclimatic stress pathway rather than as independent controls [52]. The strong association between atmospheric dryness and GPP reductions identified here highlights the importance of coupled water-energy limitations in shaping ecosystem responses to climate extremes [52]. The effects on ecosystem productivity extend beyond the heatwave period (Fig. S27). Although the magnitude of GPP reduction weakens after heatwave termination, negative GPP anomalies persist for up to 7 days, particularly following compound events in tropical, arid and temperate regions. This delayed recovery suggests that concurrent air and soil thermal stress likely induces deeper physiological and hydraulic damage. Future work should integrate physiological and hydraulic indicators to fully assess long-term consequences, including carbon starvation, hydraulic damage, delayed mortality and incomplete ecosystem recovery.

The interpretation of heatwave changes and their vegetation responses partly depend on the choice of climatological baseline. In our study, independent and compound air–soil heatwaves are identified using the 1981–2010 baseline, which is appropriate for quantifying additional above- and below-ground thermal stress relative to recent historical climate. However, if vegetation acclimation or community turnover allows ecosystems to track the evolving climate background under global warming, a sliding baseline may provide a complementary perspective. We therefore repeat the heatwave identification and GPP impact analyses using a 30-year sliding baseline [53] (Figs S37 and S38). Compound air–soil heatwaves also show significant increases in the frequency, duration and cumulative intensity, although these changes are smaller than those based on a fixed baseline. Similarly, the increasing GPP losses associated with compound heatwaves are reduced in magnitude but remain significant, and compound events retain larger GPP reductions than independent events. We note that the selection between fixed and moving climatological baselines depend on the application [53]. A fixed baseline is more appropriate for assessing additional thermal stress relative to historical climate conditions, especially for ecosystems whose thermal tolerance or structure adjusts slowly, while a sliding baseline is useful for identifying extreme events relative to an evolving climate state and may partially represent long-term ecosystem acclimation or community adjustment [54].

Several uncertainties remain in our investigation. The mechanism analysis is interpreted as a reanalysis-based process diagnostic rather than direct observational attribution, which is due to that complete long-term, collocated coverage of the land-surface and atmospheric variables needed to diagnose the mechanisms of compound air–soil heatwaves are unavailable [55]. Our major analysis focuses on the warm season, when vegetation activity and land–atmosphere coupling tend to be strongest; however, cold-season and year-round monthly sensitivity analyses show that compound air–soil heatwaves remain more frequent and longer lasting than independent air or soil heatwaves outside the warm season.

Large-scale climate variability may further modulate heatwave occurrence. Modes such as the El Niño-Southern Oscillation and the Indian Ocean Dipole can influence heatwave occurrence through changes in cloud cover, precipitation and atmospheric circulation [56,57]. Quantifying the relative contributions of remote climate drivers and local land–atmosphere feedbacks will be an important direction for future work. In addition, ecosystem responses may vary across biomes and be modified by land management and irrigation, highlighting the need for biome-specific assessments of compound heatwave impacts [9,51]. For example, irrigation can modify the water-energy pathways through which compound air–soil heatwaves affect vegetation by increasing soil water availability and evapotranspiration and shifting surface energy partitioning from sensible toward latent heat [58,59]. The datasets used in this study do not explicitly account for irrigation effects, and future work should incorporate irrigation information to better quantify how agricultural water management modulates vegetation responses to compound air–soil heatwaves. In addition, temperature-moisture coupling may change under future warming [60], altering the co-occurrence of heat and dryness relative to historical conditions, highlighting the need to account for evolving temperature-moisture coupling in future ecological risk assessments. Vegetation change may also modify the hydroclimatic background through land–atmosphere feedbacks [61], further suggesting that historical relationships among vegetation, soil properties and atmospheric conditions may not remain stationary under future environmental change.

As the climate continues to warm, compound air–soil heatwaves are projected to intensify strongly, with substantially lower risks under mitigation scenarios. Incorporating coupled above- and below-ground heat extremes into climate risk assessments will therefore be essential for improving adaptation planning, protecting ecosystem productivity and safeguarding the future land carbon sink.

## MATERIALS AND METHODS

### Reanalysis datasets and model simulations

We use daily mean near-surface air temperature (${T}_a$, 2 m above the surface) and soil temperature (${T}_s$, 0–10 cm below the surface) during 1979–2024 from the fifth-generation European Centre for Medium-Range Weather Forecasts (ECMWF) reanalysis (ERA5) [62]. To assess the robustness of our results, we additionally use the Modern-Era Retrospective analysis for Research and Applications, version 2 (MERRA2) [63] during 1980–2024. To obtain a consistent shallow soil temperature in the depth of 0–10 cm across datasets, we remap the native soil layers by applying a linear depth-weighted average (a linear interpolation), as suggested by refs. [64,65] (Text S8). Daily mean ${T}_a$ and ${T}_s$ are calculated from hourly data. We note that MERRA2 yields consistent results with ERA5 in terms of similar spatial distributions and temporal changes in three types of heatwaves (Figs 1, 3 and 4; Figs S3–>S5).

The projected changes in heatwave characteristics during 1961–2100 are examined using daily mean ${T}_a$ and ${T}_s$ outputs from the simulations of eight climate models participating in the Scenario Model Intercomparison Project within the CMIP6 (see Table S2 for details) project [66]. The ${T}_a$ and ${T}_s$ outputs over 1961–2014 utilize the historical simulations, whereas the 2015–2100 period uses the future climate projection under the intermediate (SSP2-4.5) and high-emission (SSP5-8.5) scenarios [66]. Models providing daily mean or sub-daily soil temperature are used, and the first realization (r1i1p1f1) is used for each model, as suggested by previous studies [67]. Since CMIP6 models have nonuniform soil depth definitions, ${T}_s$ is harmonized to represent the upper 0–10 cm soil layer based on each model’s native vertical structure (Table S2; Text S8). Daily ${T}_a$ and ${T}_s$ in two global reanalysis products and all climate model simulations are interpolated into a horizontal resolution of 2.5° × 2.5° using bilinear interpolation to balance detail with computational efficiency [68]. Sensitivity to spatial resolution is additionally evaluated using ERA5 dataset at its native 0.25° × 0.25° resolution (Figs S11 and S12).

### Identification of independent and compound heatwaves

Independent and compound heatwaves are identified by jointly considering daily exceedances of ${T}_a$ and ${T}_s$. Specifically, an independent air heatwave event is defined when the ${T}_a$ exceeds its corresponding calendar-specific 90th percentile threshold for at least three consecutive days while the daily ${T}_s$ does not; whereas an independent soil heatwave event occurs when ${T}_s$ exceeds its percentile threshold for at least three consecutive days in the absence of an air heatwave. A compound air–soil heatwave event is recorded when ${T}_a$ and ${T}_s$ simultaneously exceed their corresponding thresholds at the same grid cell and on the same day. The criterion for heatwaves is three or more consecutive days where:


\begin{eqnarray*}
{\mathrm{Independent\ air\ heatwave}}:{T}_a{\mathrm{\ }} > T_a^{90p}\ \& \ {T}_s{\mathrm{\ }} \le T_s^{90p};\\
{\mathrm{Independent\ soil\ heatwave}}:\ {T}_s{\mathrm{\ }} > T_s^{90p}\ \& \ {T}_a{\mathrm{\ }} \le T_a^{90p};\\
{\mathrm{Compound\ air}}-{\mathrm{soil\ heatwave}}:{\mathrm{\ }}{T}_a{\mathrm{\ }} > T_a^{90p}\ \& \ {T}_s{\mathrm{\ }} > T_s^{90p};
\end{eqnarray*}

where ${T}_a$ is the 2-m temperature and ${T}_s$ is the soil temperature at a depth of 0–10 cm. $T_a^{90p}$ and $T_s^{90p}$ are the calendar 90th percentile thresholds for ${T}_a$ and ${T}_s$, respectively.

The calendar-specific 90th percentile is calculated for each calendar day using ${T}_a$ and ${T}_s$within a 15-day window centered on the date across all years within the 30-year baseline period of 1981–2010 (i.e. a set of 15 × 30 = 450 daily values). Using a sliding 30-year climatological baseline to identify independent and compound heatwaves also yields robust results (Fig. S37). More detailed definitions and calculations of annual heatwave metrics are provided in Text S8.

### Physical processes of independent and compound heatwaves

We decompose daily anomalies of near-surface air temperature and soil temperature in the depth of 0–10 cm based on surface energy budget to diagnose the local thermodynamic and dynamical processes associated with each type of heatwaves (Texts S2 and S3). Near-surface air temperature is characterized by horizontal advection of air from climatologically warmer regions, adiabatic warming associated with subsiding motion and diabatic heating due to radiative and turbulent fluxes [29,69]. In contrast, soil temperature in the depth of 0–10 cm is primarily related to diabatic processes, including variations in surface radiation, latent and sensible heat exchange and conductive heat transfer into deeper layers. To characterize the hydroclimatic conditions accompanying each type of heatwaves, we conduct composite analyses of atmospheric circulation, surface radiation, turbulent heat fluxes, soil moisture, VPD, evaporation and related land–atmosphere variables from ERA5 dataset [31,32] (Text S4). Daily anomalies of each variable are calculated after removing the climatological seasonal cycle and long-term variations, and individual heatwave events are averaged with equal weight.

### Impacts of heatwaves on GPP

To assess how independent and compound heatwaves affect terrestrial carbon uptake, we calculate GPP anomalies associated with each type of heatwaves. We extract the minimum GPP anomaly during each heatwave episode at each grid cell and then average these anomalies over the available GPP record [70]. Daily GPP dataset utilized in this study is derived from the Breathing Earth System Simulator (BESS) v2.0 product [71], a satellite-driven model integrating coupled carbon, water and energy processes. The product provides spatially continuous, long-term GPP estimates at 0.05° resolution for 1982–2022, enabling consistent characterization of productivity anomalies associated with heatwave events. To match the resolution of the heatwave events data, we regrid the GPP dataset to 2.5° × 2.5° using area-weighted aggregation. To test the robustness of our findings, we additionally use two additional GPP products (i.e. Boreal Ecosystem Productivity Simulator (BEPS) [72] and the FluxSat GPP v2.2 product [73]) and repeat the analysis at 0.25° × 0.25° resolution (Text S5; Figs S39–>S41). These sensitivity analyses yield consistent results, indicating that the estimated GPP responses are insensitive to the choice of GPP product and spatial resolution.

To provide a detailed picture of how terrestrial carbon uptake is modulated by different types of heatwaves and their background conditions, we construct separate random forest regression models [39] for independent air heatwaves, independent soil heatwaves and compound air–soil heatwaves (Text S6). Predictors include surface energy contributions to near-surface air and shallow soil temperature anomalies, along with background hydroclimatic conditions such as cloud cover, soil moisture, VPD and evaporation. Each grid cell with a statistically significant GPP response is treated as one sample in the machine learning analysis for the corresponding heatwave type. Samples are randomly partitioned into training (80%) and testing (20%) subsets, and model performance is evaluated using the coefficient of determination (*R*²), root mean square error, explained variance and out-of-bag performance (Table S1). Predictor contributions are interpreted using SHAP [40] values calculated for the independent testing samples.

### Trend estimation

The long-term trends in heatwave characteristics are evaluated using the Theil–Sen slope estimator [74], and their statistical significance is evaluated by a modified non-parametric Mann–Kendall test [75]. These non-parametric methods are robust to missing values and outliers, and have been widely applied in previous climate change studies [53,76]. To quantify the uncertainty of the trends, we apply bootstrap resampling (1000 iterations) to calculate standard errors and 95% confidence intervals for the estimated slopes.

## Supplementary Material

nwag603_Supplemental_File

## Data Availability

All data used in this study are publicly available under the following URLs. The ERA5 dataset is available via the Copernicus Climate Data Store (CDS) at https://cds.climate.copernicus.eu/cdsapp#!/dataset/reanalysis-era5-complete?tab=form. The MERRA2 data are available at https://disc.gsfc.nasa.gov/datasets/M2T1NXSLV_5.12.4/summary. Data for historical simulations (1961–2014) and future projections (2015–2100) of daily near-surface air temperature and daily (or sub-daily) soil temperature are from the sixth phase of the Coupled Model Intercomparison Project Phase 6 (CMIP6) (https://esgf-node.llnl.gov/search/cmip6/). BESS v2.0 GPP data are downloaded from https://drive.google.com/drive/folders/1ZJWSwBDcz9mHU2FXXtUTpAMrjstj1b6n. BEPS GPP model data are downloaded from https://www.nesdc.org.cn/sdo/detail?id=612f42ee7e28172cbed3d809. FluxSat GPP data are downloaded from https://doi.org/10.5067/MEASURES/FLUXSAT/FLUXSATMGPP_L3_DAILY_P5DEG.2.2. The codes used for this study are publicly available in a Zenodo repository (https://doi.org/10.5281/zenodo.22725608).
