# Supplementary material for: Emerging compound air–soil heatwaves amplify global vegetation losses
Source: Natl Sci Rev. 2026 Sep 17;13(20):nwag603. doi: 10.1093/nsr/nwag603 (PMC13702032; doi:10.1093/nsr/nwag603)
Supplement: Supplementary file 1 [file nwag603_supplemental_file.pdf]

Supplementary Information for

Emerging compound air-soil heatwaves amplify global vegetation losses

Sijia Wu[1,†], Ming Luo[1,†,*], R. Iestyn Woolway[2,†], Zhen Liu[3], Xing Li[1], Haicheng Zhang[1], Weilin Liao[1], Xiaoyu Wang[1], Manqing Shi[1], Qiuting Wang[1], Tao Pei[4,5,6*], Xiaoping Liu[1*]

1. Guangdong Provincial Key Laboratory of Urbanization and Geo-Simulation, School of Geography and Planning, Sun Yat-sen University, Guangzhou, 510006, China.

2. School of Ocean Sciences, Bangor University, Menai Bridge, LL59 5AB.

3. Earth, Ocean and Atmospheric Sciences (EOAS) Thrust, Function Hub, The Hong Kong University of Science and Technology (Guangzhou), Guangzhou, 511453, China.

4. State Key Laboratory of Resources and Environmental Information System, Institute of Geographic Sciences and Natural Resources Research, Chinese Academy of Sciences, Beijing, 100101, China.

5. University of Chinese Academy of Sciences, Beijing, 101408, China.

6. Jiangsu Center for Collaborative Innovation in Geographical Information Resource Development and Application, Nanjing, 210023, China.

*Correspondence: M.L. (luom38@mail.sysu.edu.cn), X.L. (liuxp3@mail.sysu.edu.cn), and T.P. (peit@lreis.ac.cn).

†. These authors contribute equally to the work.

Content:

1. Supplementary Texts S1–S8.

2. Supplementary Figures S1–S41.

3. Supplementary Table S1–S2.

Supplementary Text 1. Methodological details and sensitivity analyses of heatwave identification

Under the ‘pure’ heatwave classification adopted in this study, independent heatwave events are identified outside the window of compound air-soil events. We acknowledge that some warm spells exhibit ‘hybrid’ characteristics. For instance, a sequence where T_a and T_s exceedances alternate or overlap in a way that does not satisfy the 3-day consistency required for any single pure type. Such ‘hybrid’ sequences are therefore excluded from our primary analysis to maintain the conceptual distinction among the three heatwave types. Our definition of independent heatwaves differs from the conventional T_a-only (or T_s-only) definitions used in previous studies [1, 2], which typically ignore the condition of soil (or air) heat. Accordingly, independent air or soil heatwaves reported here may be weaker or less frequent than T_a-only (or T_s-only) heatwaves in literature [1], as days of concurrent exceedance are partitioned into the compound category rather than independent events.

To characterize independent and compound heatwaves, we quantify their occurrence frequency (events), mean duration (days), maximum intensity (°C), cumulative intensity (°C×days), and affected area (km²) during summertime (May–September in the Northern Hemisphere and November–March in the Southern Hemisphere). For each grid cell, the annual frequency of each type of heatwave is defined as the total number of events occurring during summer of each year. Mean duration is the average event length of all events in each year. Heatwave intensity is defined as the exceedance above the varying percentile threshold, and the maximum intensity is taken as the largest values of event-level maxima within that year. Cumulative intensity is calculated as the sum of intensity over the duration of every heatwave event in each year. Affected area is the sum of the areas of all land grid cells experiencing a heatwave event on that day. Annual maximum and mean affected areas are calculated as the maximum and mean of daily affected area of the heatwave events in each year, respectively. We calculate the spatially-averaged means for different climate zones identified by the Köppen-Geiger climate classification system [3],

excluding Antarctica and polar regions where the presence of permafrost may influence the soil temperature.

To test the robustness of our classification, we evaluate the robustness of independent and compound heatwave identification to the choice of climatological baseline, temperature threshold, temperature metric, and seasonal definition. In addition to the fixed 1981–2010 reference period used in the primary analysis, heatwaves are identified using a sliding 30-year baseline. For each target year, calendar specific 90th percentile thresholds of daily mean near-surface air temperature (T_a, 2 m above the surface) and soil temperature (T_s, 0–10 cm below the surface) are calculated from the corresponding 30-year reference period centered in that year. For years near the beginning or end of the record, the nearest available 30-year window is used (Supplementary Figs. S37–S38). Robustness to threshold choice is assessed by repeating the analysis using a higher threshold at the 95th percentile (Supplementary Figs. S6–S8). We also identify independent and compound heatwaves during the cold season, which is defined as November–March in the Northern Hemisphere and May–September in the Southern Hemisphere, and further conduct a year-round monthly analysis (Supplementary Figs. S13–S16). These sensitivity analyses yield results consistent with the primary analysis, indicating that our main findings are insensitive to the choice of climatological baseline, temperature threshold, and seasonal definition.

Supplementary Text 2. Diagnostic decomposition of near-surface air temperature anomalies

At each land grid cell, near-surface air temperature anomalies (ΔTa) is decomposed into four components: horizontal temperature advection heating (ΔT_{advec}), adiabatic heating (ΔT_{adiab}), diabatic heating (ΔT_{diab}), and a residual term (ΔT_{res}) [4, 5]. The decomposition is expressed as [5, 6]

$$\Delta Ta = \Delta T_{advec} + \Delta T_{adiab} + \Delta T_{diab} \tag{1}$$

The residual term is calculated as:

$$\Delta T_{res} = \Delta Ta - (\Delta T_{advec} + \Delta T_{adiab} + \Delta T_{diab}) \tag{2}$$

Advection heating (ΔT_{advec}) represents the effect of horizontal transport of air masses with different temperatures. In pressure coordinates, it is diagnosed as [5]:

$$\Delta T_{advec,pl} = -\Delta t \left(u_{pl} \frac{\partial T_{pl}}{\partial x} + v_{pl} \frac{\partial T_{pl}}{\partial y} \right) \tag{3}$$

where u_{pl} and v_{pl} are the zonal and meridional wind components, respectively; $\frac{\partial T_{pl}}{\partial x}$ and $\frac{\partial T_{pl}}{\partial y}$ are the temperature gradients in the zonal and meridional directions; and Δt is the time step (i.e., one day in this study); pl denotes the given pressure level.

Adiabatic heating (ΔT_{adiab}) describes temperature changes linked to vertical motion and the background vertical temperature structure [5], which is calculated as [5]:

$$\Delta T_{adiab,pl} = -\Delta t\, \omega_{pl} \left(k \frac{T_{pl}}{P_s} + \frac{\partial T_{pl}}{\partial p} \right) \tag{4}$$

where ω_{pl} and T_{pl} are the vertical velocity and air temperature at a given pressure level, respectively; P_s is the surface pressure, and k is Poisson's constant (i.e., 0.286). $\omega_{pl} k \frac{T_{pl}}{P_s}$ describes the ideal adiabatic temperature change resulting from vertical motion in the atmosphere. $\omega_{pl} \frac{\partial T_{pl}}{\partial p}$ quantifies how the vertical motion of air interacts with the existing vertical temperature gradient (i.e., $\frac{\partial T_{pl}}{\partial p}$) to cause temperature changes.

The calculations of advection heating and adiabatic heating are performed at four pressure levels (i.e., 1000 hPa, 850 hPa, 700 hPa, and 600 hPa), covering the lower and mid-troposphere [5]. The selection of pressure level (i.e., pl) depends on the surface pressure of the location where heatwaves occur, which is influenced by topography.

The diagnosed diabatic contribution to near-surface air temperature (ΔT_{diab}) is highly related to the variations in skin temperature (ΔT_{skin}) [7]. Their relationship is represented by a factor f, which quantifies how skin temperature variations translate into the changes in air temperature (Eq. (5)) [8], and is typically assumed to be 1 in previous studies [5, 6, 8].

$$\Delta T_{diab} = f \cdot \Delta T_{skin} \tag{5}$$

Based on land surface energy, the diabatic heating process can be further decomposed as follows:

$$H_g = H_s + H_{ld} + H_{lu} + H_{lh} + H_{sh} \tag{6}$$

where H_s is the net solar radiation, H_{ld} and H_{lu} are the downward and the emitted upward longwave radiations, respectively, H_{lh} and H_{sh} are the surface latent and sensible heat fluxes, respectively. Note that all radiation and flux terms are downward positive [9].

Taking the differential of both sides in Eq. (6), the changes in surface energy budget can be written as

$$\Delta H_g = \Delta H_s + \Delta H_{ld} + \Delta H_{lu} + \Delta H_{lh} + \Delta H_{sh} \tag{7}$$

Expressing H_{lu} as $\sigma \cdot T_{skin}^4$ where σ is the Stefan-Boltzmann constant (i.e., 5.67×10^{-8}W m^{-2} k^{-4}), and taking its differential, Eq. (7) can be written as

$$\Delta T_{skin} = \frac{(\Delta H_s + \Delta H_{ld} + \Delta H_{lh} + \Delta H_{sh} - \Delta H_g)}{4\sigma \cdot \bar{T}_{skin}^3} \tag{8}$$

Combining Eqs. (1) and (8), the contributions to the near-surface air temperature anomalies (Ta) during heatwaves are decomposed into $\Delta T_{advec,pl}$, $\Delta T_{adiab,pl}$, $\frac{\Delta H_s}{4\sigma\cdot\bar{T}_{skin}^3}$, $\frac{\Delta H_{ld}}{4\sigma\cdot\bar{T}_{skin}^3}$, $\frac{\Delta H_{lh}}{4\sigma\cdot\bar{T}_{skin}^3}$, $\frac{\Delta H_{sh}}{4\sigma\cdot\bar{T}_{skin}^3}$, $\frac{\Delta H_g}{4\sigma\cdot\bar{T}_{skin}^3}$, and ΔT_{res}.

Supplementary Text 3. Diagnostic decomposition of soil temperature anomalies

The soil temperature anomalies (ΔTs) in the 0–10 cm layer are decomposed using a one-layer heat storage model [10]. The 0–10 cm soil layer is treated as a homogeneous slab with volumetric heat capacity $\rho_s c_s$ and thickness $\Delta z = 0.1$ m. The volumetric heat capacity is fixed at $\rho_s c_s \approx 2.19 \times 10^6\ Jm^{-3}K^{-1}$, as recommended by Ref.[10], which lies within the observed range for soils. Under this approximation, the layer-integrated anomalous heat budget is written as:

$$\rho_s c_s \Delta z \frac{\partial \Delta Ts}{\partial t} = \Delta H_{sfc} - \Delta H_{1\to2} \tag{9}$$

where ΔH_{sfc} is the anomalous net surface heat flux into the soil (downward positive), and $\Delta H_{1\to2}$ is the diagnosed residual subsurface heat exchange term (i.e., the

anomalous conductive flux from the upper layer into deeper layers). Over a daily time step Δt, we approximate $\frac{\partial \Delta Ts}{\partial t} \approx \frac{\Delta Ts}{\Delta t}$, so that Eq. (9) is written as:

$$\Delta Ts = \frac{\Delta t}{\rho_s c_s \Delta z}\left(\Delta H_{sfc} - \Delta H_{1\to 2}\right) \tag{10}$$

Consistent with the air-temperature decomposition, the anomalous net surface heat flux into the soil is written as:

$$\Delta H_{sfc} = \Delta H_s + \Delta H_{ld} + \Delta H_{lh} + \Delta H_{sh} \tag{11}$$

Combining Eqs. (10) and (11), the contributions to the soil temperature anomalies (Ts) during heatwaves are decomposed as follows:

$$\Delta Ts = \frac{\Delta t}{\rho_s c_s \Delta z}\left(\Delta H_s + \Delta H_{ld} + \Delta H_{lh} + \Delta H_{sh} - \Delta H_{1\to 2}\right) \tag{12}$$

which expresses the contributions to the soil temperature anomalies (ΔTs) as the sum of temperature-equivalent contributions from shortwave, longwave, latent and sensible heat flux anomalies at the surface and from conductive exchange with deeper layers.

In the air temperature budget (Supplementary Figs. S18 and S20–S22), advective and adiabatic heating are comparatively small and partly compensating, whereas diabatic heating provides the largest positive contribution and is stronger during compound extremes (+4.38 °C) relative to independent air (+2.71 °C) and independent soil (+2.73 °C) heatwaves. This diabatic warming is mainly associated with enhanced net shortwave and downward longwave radiation, both of which peak in cold regions (+7.88 °C and +4.16 °C, respectively), in line with the cold regions experiencing the strongest cumulative heatwave intensity (recall Fig. 1). Soil warming is stronger during compound air-soil heatwaves (+3.68 °C) and independent soil heatwaves (+2.72 °C), but is weaker during independent air heatwaves (+1.64 °C) (Supplementary Figs. S19 and S20–S22). As for air temperature, soil warming is mainly accompanied by enhanced net shortwave and downward longwave radiation, and is partly offset by cooling from turbulent heat exchange and conductive heat transfer into deeper soil layers (ground heat flux).

Supplementary Text 4. Composite analysis and regional dependence of land-atmosphere conditions during heatwaves

To characterize the hydroclimatic background accompanying each type of heatwaves, we employ composite analysis to examine the key atmospheric and surface variables associated with independent air heatwaves, independent soil heatwaves, and compound air-soil heatwaves [11, 12]. The key variables used in this study include skin temperature (T_{skin}), total cloud cover (Cloud), vapor pressure deficit (VPD), surface net shortwave radiation (SWR), surface downward longwave radiation (LWR), total precipitation (TP), surface soil moisture (SM), surface latent heat flux (LHF), surface sensible heat flux (SHF), and surface pressure (Ps) from the single-level ERA5 dataset. Additionally, we incorporate air temperature (T_{pl}), and vertical velocity (ω_{pl}) at the 1000 hPa, 850 hPa, 700 hPa, and 600 hPa levels, and horizontal wind components (u_{pl} and v_{pl}) at various pressure levels. The VPD is defined as the difference between the saturated vapor pressure and the actual vapor pressure in the atmosphere, determined

by near-surface air temperature and dew-point temperature. Therefore, we use the daily T_a and dew-point temperature data from the ERA5 to calculate daily VPD.

In this analysis, we first calculate the daily anomalies of each variable by removing the climatological seasonal cycle from the original daily series. The seasonal cycle is obtained from the multi-year average for each calendar day over the reference period of 1981–2010, with a 15-day rolling mean applied to exclude possible short-term fluctuations [13]. To exclude the effects of long-term climate warming on the analysis the composite anomalies, we detrend the time series of anomalies of each variable using a third-degree polynomial fitting method. We then calculate the composite anomalies for a particular type of heatwave by averaging daily variable anomalies during all participating days throughout a certain type of heatwave event, and then averaging all heatwave events with equal weight. This computation is repeated for each type of heatwave events, with the significance of these anomalies evaluated by the Student's *t*-test.

Although all heatwave types are associated with reduced cloud cover, depleted soil moisture and elevated vapor pressure deficit, their magnitudes differ among heatwave types (Supplementary Fig. 23). Independent air and compound heatwaves show larger cloud reductions and vapor pressure deficit increases, consistent with a stronger radiative contribution to warming and enhanced atmospheric evaporative demand. In contrast, independent soil and compound heatwaves show more pronounced soil moisture depletion, indicating a stronger land-surface constraint on evapotranspiration and latent cooling. These feedbacks differ between moisture-limited and energy-limited regimes. In energy-limited regimes (e.g., the Amazon and parts of North Africa and the Middle East), ample soil moisture sustains evaporation and moderates warming, whereas in moisture-limited regimes (including southern Africa, southern South America, and India), soil moisture losses suppress evaporative cooling and promote sensible heating, amplifying temperature anomalies through positive soil moisture-temperature feedbacks.

Supplementary Text 5. GPP responses to independent and compound heatwaves

We evaluate the robustness of GPP responses to independent and compound heatwaves using two additional daily GPP products. The Boreal Ecosystem Productivity Simulator (BEPS) [14] provides daily GPP at a horizontal resolution of 0.072727° × 0.072727° covering 1981–2019 and the FluxSat GPP version 2.2 (FluxSat GPP v2.2) product [15] provides daily GPP at a horizontal resolution of 0.5°×0.625° covering the period from March 2000 to the present. Consistent with the primary analysis based on BESS v2.0, both products yield consistent spatial patterns and changes of GPP anomalies in response to independent and compound heatwaves (Fig. 2; Supplementary Figs.S39–S40). In addition, considering that ERA5 provides the temperature fields at a native resolution of 0.25°×0.25°, we also perform a sensitivity analysis by regridding the GPP products to the 0.25° resolution as well. The resulting GPP responses to independent and compound heatwaves are consistent with those obtained at the 2.5°×2.5° resolution (Fig. 2; Supplementary Fig. S41).

At each grid cell, we first detrend the GPP time series by subtracting the slope of linear regression during the period to remove the fertilizing effects of rising CO_2 on vegetation productivity [16, 17]. The GPP time series in which the temporal linear regression is significantly positive (slope > 0 and p-value < 0.05) are exclusively detrended, and the series are left as original if the linear regression is negative or insignificant. Daily GPP anomalies are then calculated by removing the climatological seasonal cycle from daily GPP time series [18, 19]. The seasonal cycle is obtained from the multi-year average for each calendar day over the reference period (i.e., 1982–2010 for BESS v2.0, 1981–2010 for BEPS, and 2001–2024 for FluxSat GPP v2.2), with a 15-day rolling mean applied to exclude possible short-term fluctuations. The sensitivity of GPP impacts to an evolving climatic baseline is evaluated by recalculating daily GPP anomalies using the corresponding 30-year sliding baseline (Supplementary Fig. S38). These analyses indicate that our main findings are insensitive to the choice of GPP product, spatial resolution, and climatological baseline.

To examine delayed GPP responses, we track daily GPP anomalies for seven days following heatwave termination. Events followed by another heatwave within these

seven days are excluded to avoid interference from renewed heat stress. To evaluate how GPP responses differ between independent and compound heatwaves, we conduct a comparison analysis of controlling intensity and duration of heatwave event. To test whether the GPP response to compound air-soil heatwaves departs from the sum of the responses associated with independent air and soil heatwaves, we introduce an interaction analysis based on a two-by-two factorial interaction contrast [20]. At each grid cell, days are classified into four mutually exclusive states: no heatwave (i.e., 00), independent air heatwave (i.e., 10), independent soil heatwave (i.e., 01), and compound air-soil heatwave (i.e., 11). The expected additive response to a compound event is calculated as:

$$GPP_{add} = \overline{GPP_{10}} + \overline{GPP_{01}} - \overline{GPP_{00}} \tag{13}$$

and the interaction effect is defined as:

$$I = \overline{GPP_{11}} - GPP_{add} = \overline{GPP_{11}} - (\overline{GPP_{10}} + \overline{GPP_{01}} - \overline{GPP_{00}}) \tag{14}$$

where $\overline{GPP_{00}}$, $\overline{GPP_{10}}$, $\overline{GPP_{01}}$, and $\overline{GPP_{11}}$ denote the mean daily GPP anomalies under the four states, respectively. A negative I indicates that the compound response is more negative than expected from additivity, whereas a positive I indicates a sub-additive response.

Annual GPP loss is calculated at each grid cell as the annual sum of negative daily GPP anomalies over all heatwave days [21], averaged across all valid vegetation grid cells within each region. During 1982–2022, the global annual GPP loss associated with compound air-soil heatwaves exhibits a significant strengthening trend of 0.61 gC m^{-2} yr^{-1} $decade^{-1}$, which is more than ten times the corresponding trend for independent soil heatwaves (0.05 gC m^{-2} yr^{-1} $decade^{-1}$). This outpacing is concentrated in low- and mid-latitude climate zones. The largest increases in annual GPP loss during compound heatwaves occur in the tropics (2.05 gC m^{-2} yr^{-1} $decade^{-1}$) and temperate regions (1.53 gC m^{-2} yr^{-1} $decade^{-1}$), followed by arid regions (0.74 gC m^{-2} yr^{-1} $decade^{-1}$). Across these zones, trends in annual GPP loss during independent soil heatwaves are consistently weaker than those during compound heatwaves. These results indicate that long-term productivity losses during compound heatwaves are increasing more rapidly than those during independent soil heatwaves. Consistent with these regional contrasts,

compound heatwaves are becoming both more frequent and more widespread, coinciding with increasingly severe and spatially extensive reductions in terrestrial carbon uptake.

Supplementary Text 6. Random forest modelling and SHAP interpretation

To characterize the environmental conditions associated with GPP responses during different heatwave types, we construct separate random forest regression models for independent air heatwaves, independent soil heatwaves, and compound air-soil heatwaves [22]. The same predictor set is used for the three models and comprises three categories: (1) diabatic surface-energy contributions to near-surface air temperature anomalies, represented by composite anomalies of net surface shortwave radiation (ΔTa_{SWR}), surface downward longwave radiation (ΔTa_{LWR}), surface latent heat flux (ΔTa_{LHF}), surface sensible heat flux (ΔTa_{SHF}), and ground heat flux (ΔTa_{Q}); (2) surface energy contributions to soil temperature anomalies (i.e., ΔTs_{SWR}, ΔTs_{LWR}, ΔTs_{LHF}, ΔTs_{SHF}, and ΔTs_{Q}); and (3) background hydrometeorological conditions, including anomalies of total cloud cover ($\Delta Cloud$), soil moisture (ΔSM), vapor pressure deficit (ΔVPD), and evaporation (ΔE). In this way, the random forest models characterize how GPP anomalies during each type of heatwaves are associated with the combined configuration of atmospheric and soil energy contributions together with background conditions.

Each grid cell with a statistically significant GPP response is treated as one sample for the corresponding heatwave type. For each type of heatwave, we randomly partition the samples into training (80%) and testing (20%) subsets and fit an independent random forest ensemble. We evaluate ensembles with 200, 300, 400 and 500 trees, and select the number of trees according to the highest out-of-bag R^2 calculated from the training data. Model performance is evaluated using the coefficient of determination (R^2), root mean square error (RMSE), and explained variance (EV) for both the training and testing subsets, together with out-of-bag performance metrics (Supplementary Table S1). We use the SHAP (SHapley Additive exPlanations) [23] values to interpret the random forest models. Tree SHAP is calculated for the independent testing samples,

using 200 randomly selected training samples as the background distribution. SHAP values quantify the contribution of each predictor to the model predicted GPP anomaly for each grid cell. Predictors are ranked by mean absolute SHAP values. SHAP beeswarm plots show the magnitude and direction of predictor contributions across testing samples, whereas SHAP dependence plots characterize their nonlinear relationships with model predictions. Because land-atmosphere predictors can be strongly correlated during heatwave events, SHAP values are interpreted as model-based predictive associations within the full multivariate model, rather than as independent physical or causal effects.

Supplementary Text 7. Distributional shifts between the earlier and recent periods

The comparisons between the earlier (1981–2010) and recent (2011–2024) periods provide additional evidence for the rapid intensification of heatwaves (Supplementary Fig. 35). The probability density function for compound heatwaves shows a pronounced rightward shift, with mean occurrence frequency increasing by 1.72 events (+140.1%), mean duration by 0.60 days (+14.0%), maximum intensity by 0.50 °C (+20.6%), and cumulative intensity by 13.84 °C×days (+109.5%). These shifts are substantially larger than those observed for independent heatwaves, underscoring the growing dominance of compounding air-soil heat hazards in recent decades.

Supplementary Text 8. Harmonization of shallow soil temperature and spatial resolution sensitivity

Soil temperature datasets differ in their native vertical representations. To obtain a consistent shallow soil temperature in the depth of 0–10 cm across datasets, we remap the native soil layers by applying a linear depth-weighted average (a linear interpolation), as suggested by Refs.[24, 25] Specifically, for ERA5, which provides data in depth intervals, we directly apply depth-based weighted averaging within each segment. For MERRA2, which provides data at discrete depths (0.0988, 0.1952, 0.3859, 0.7626, and 1.5071 m), we define depth intervals by taking the midpoint between consecutive layers before performing weighted averaging [24, 25]. CMIP6 models have

nonuniform soil depth definitions (Supplementary Table S2). For models with a native depth coordinate at 5 cm, this value is used as the representative temperature of the upper 0–10 cm layer, because 5 cm corresponds to the midpoint of this layer. For models with multiple depth coordinates within or around the upper 0–10 cm, we apply a depth-weighted averaging method based on the native layer structure [24, 25].

Daily T_a and T_s in two global reanalysis products and all climate model simulations are interpolated into a common resolution of 2.5°×2.5° by the bilinear interpolation to balance detail with computational efficiency [26]. To evaluate sensitivity to spatial resolution, we further identify heatwaves using ERA5 dataset at its native 0.25°×0.25° resolution. The resulting climatology and long-term changes of independent and compound heatwaves are consistent with those based on the 2.5°×2.5° resolution (Supplementary Figs. S11–S12), indicating that the main findings are insensitive to the spatial resolution used in the primary analysis.

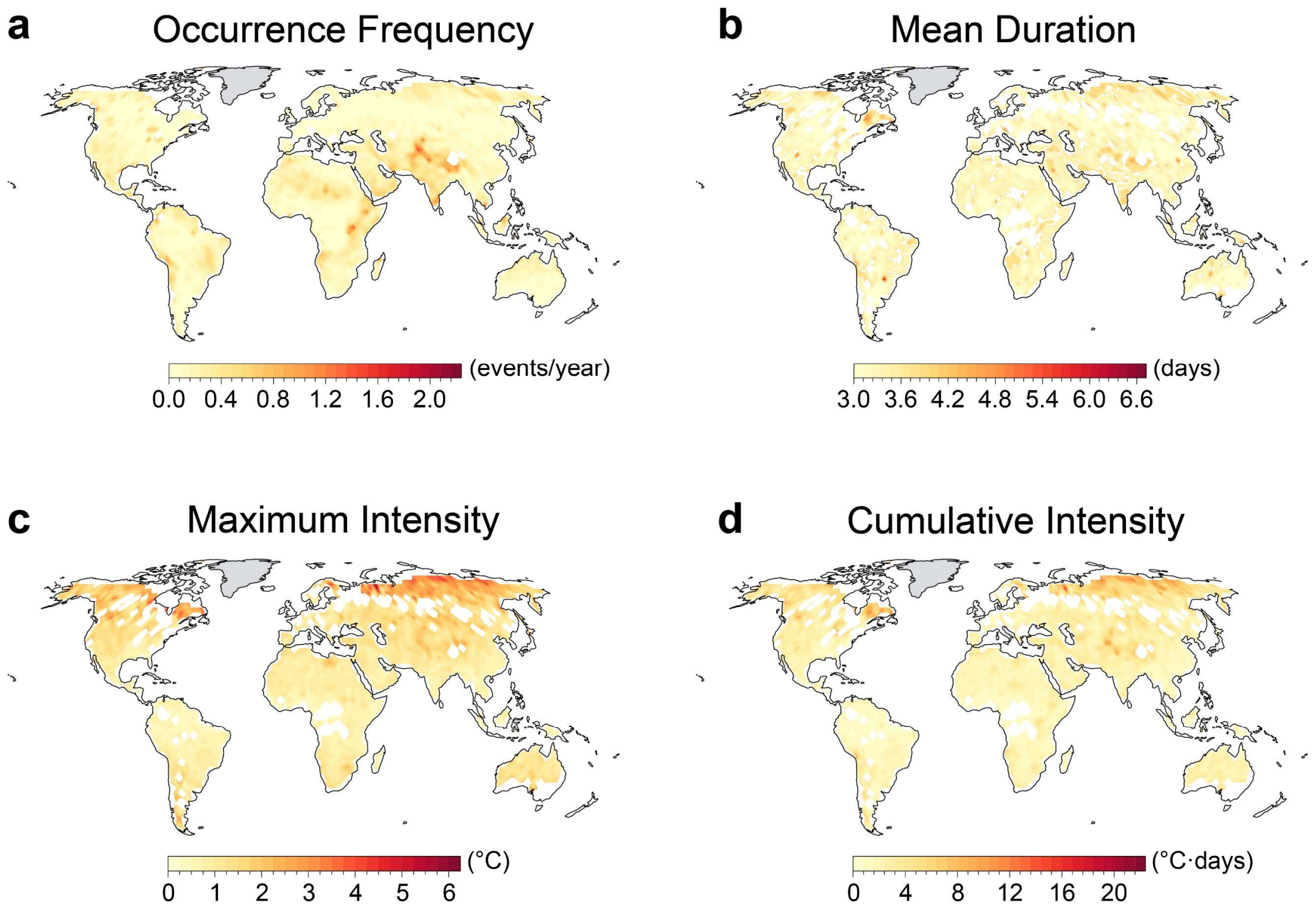

Supplementary Fig. 1 Observed climatology of independent air heatwaves. a–f, Spatial distribution of the climatological mean of annual mean occurrence frequency (**a**), mean duration (**b**), maximum intensity (**c**), and cumulative intensity (**d**) of independent air heatwaves over global land areas from 1979 to 2024.

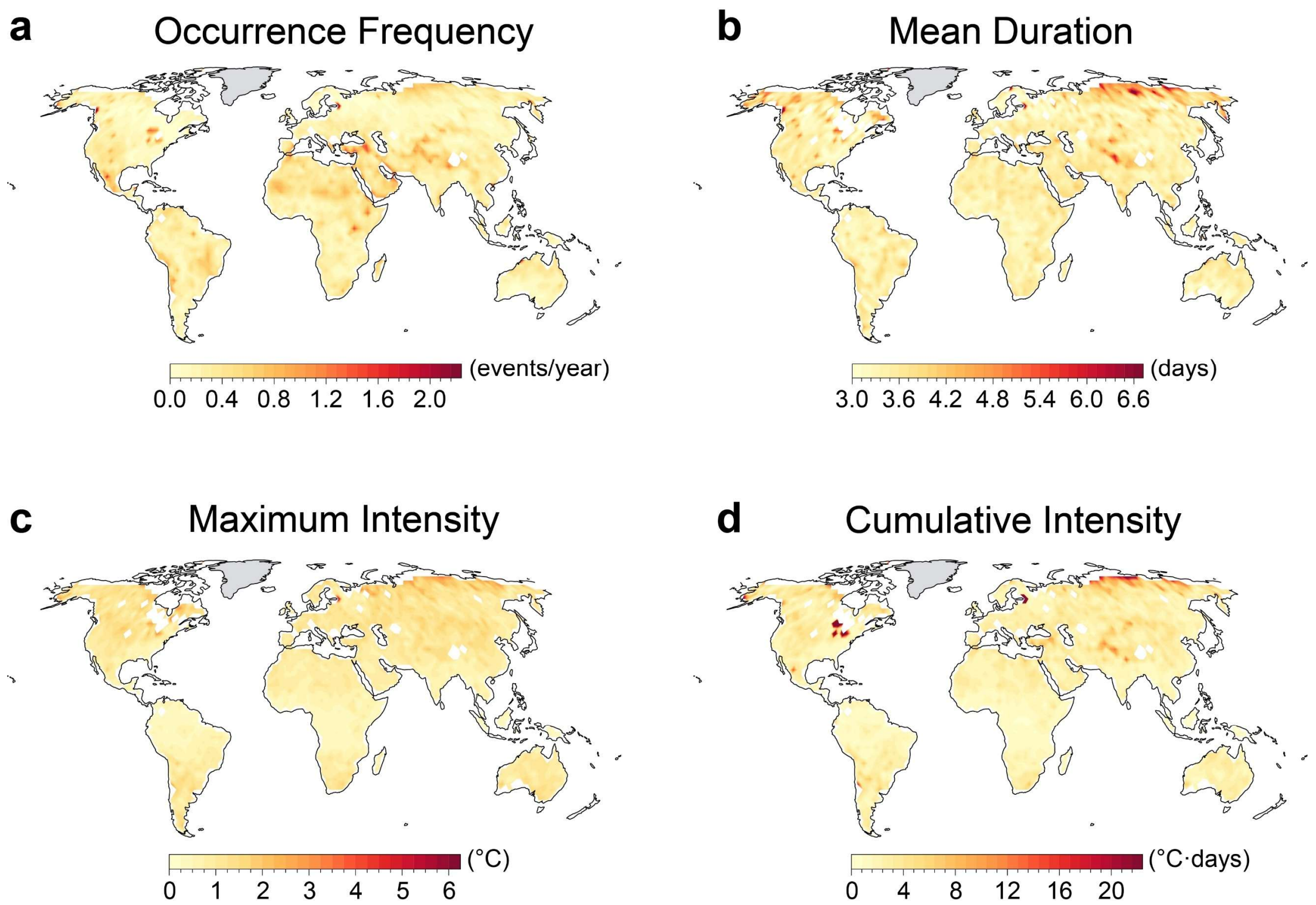

Supplementary Fig. 2 Observed climatology of independent soil heatwaves. a–f, Spatial distribution of the climatological mean of annual mean occurrence frequency (**a**), mean duration (**b**), maximum intensity (**c**), and cumulative intensity (**d**) of independent soil heatwaves over global land areas from 1979 to 2024.

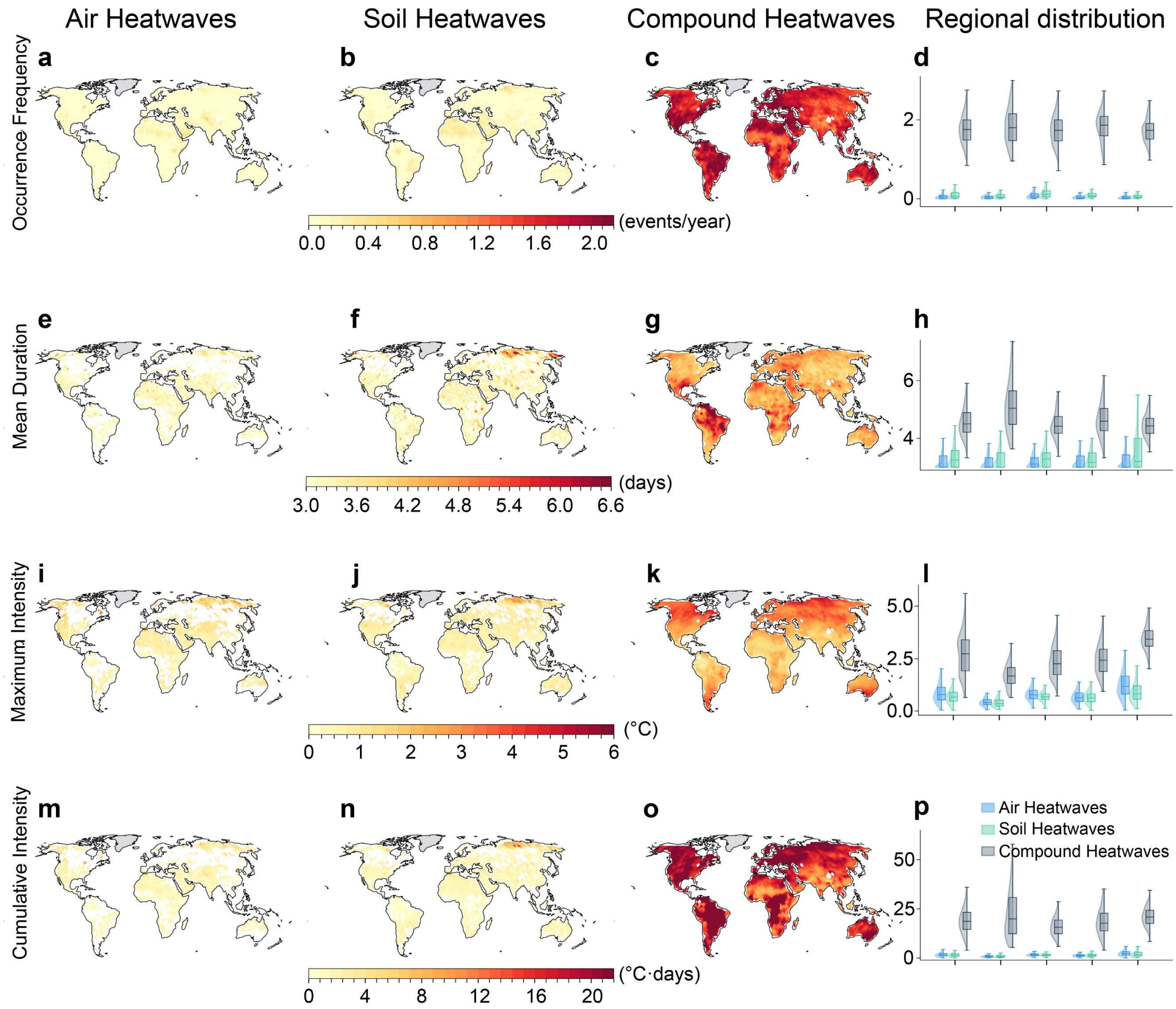

Supplementary Fig. 3 Observed climatology of independent and compound air-soil heatwaves during 1980–2024 based on the MERRA2 dataset. a–w, Maps showing the climatological mean of annual mean occurrence frequency (**a–c**), mean duration (**e–g**), maximum intensity (**i–k**), and cumulative intensity (**m–o**) of independent air heatwaves (left column), independent soil heatwaves (middle column), and compound air-soil heatwaves (right column) over global land areas during 1979–2024. **d**, **h**, **l**, **p**, violin and box plots showing the distributions of climatological mean of the corresponding metrics of independent air heatwaves (blue), independent soil heatwaves (green), and compound air-soil heatwaves (grey) over global land areas and across different climate zones (Glob: global, Trop: tropical zone, Arid: arid zone, Temp: temperate zone, and Cold: cold zone). The centerline indicates the median value, the box bounds depict the 25th and 75th percentile values and the whiskers indicate the lower and upper quartiles±1.5 times the interquartile range of the values.

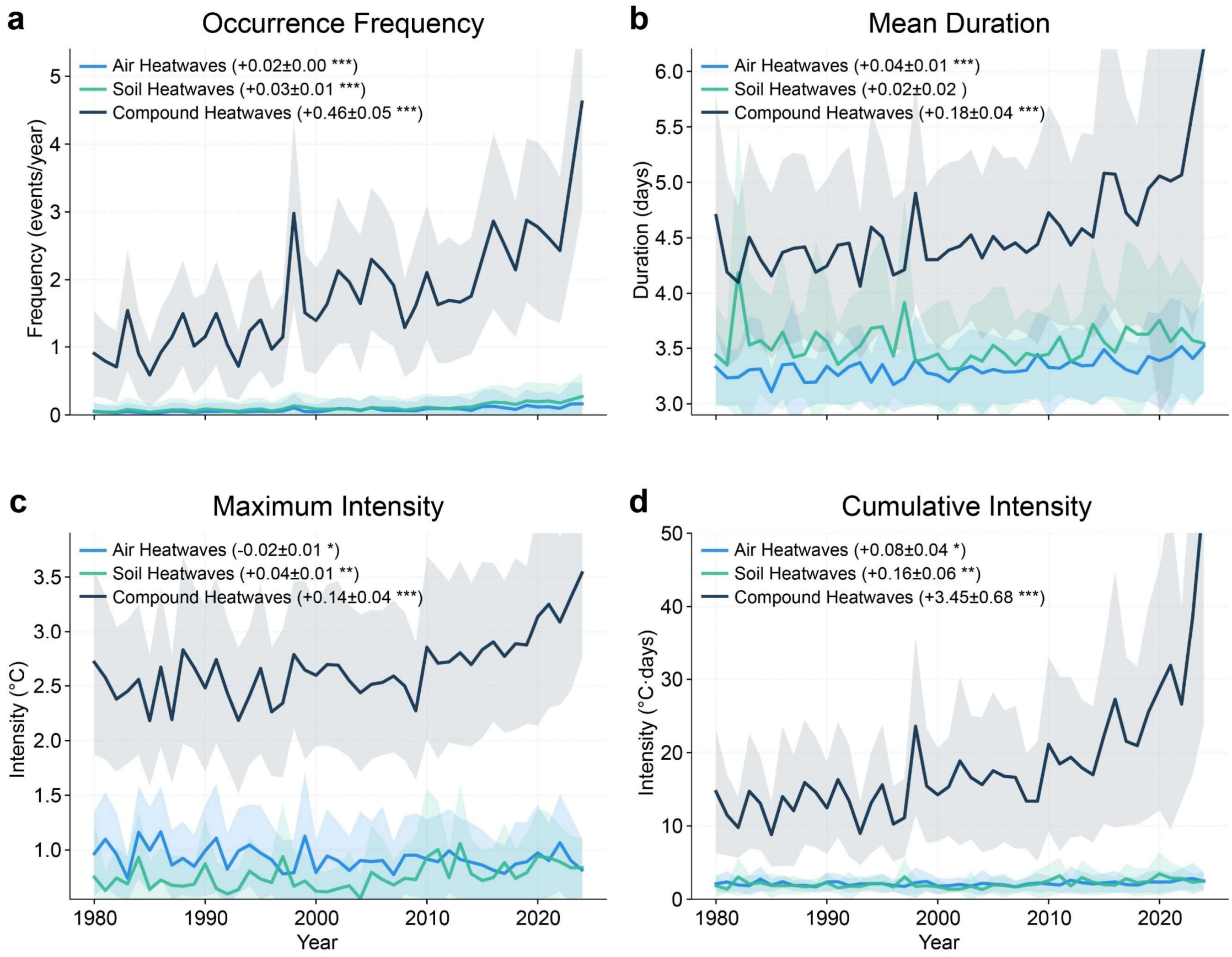

Supplementary Fig. 4 Long-term changes in independent and compound air-soil heatwaves during 1980–2024 based on the MERRA2 dataset. a–f, Time series of the spatially averaged annual mean of occurrence frequency (**a**), mean duration (**b**), maximum intensity (**c**), and cumulative intensity (**d**) of independent air heatwaves (blue), independent soil heatwaves (green), and compound air-soil heatwaves (grey) over global land areas from 1980 to 2024. Solid line represents the time series of regional mean and the shaded regions represent half of the standard deviation. Slope estimates for the trend per decade are given in parentheses. *$p < 0.05$ for long-term trend; **$p < 0.01$; ***$p < 0.001$.

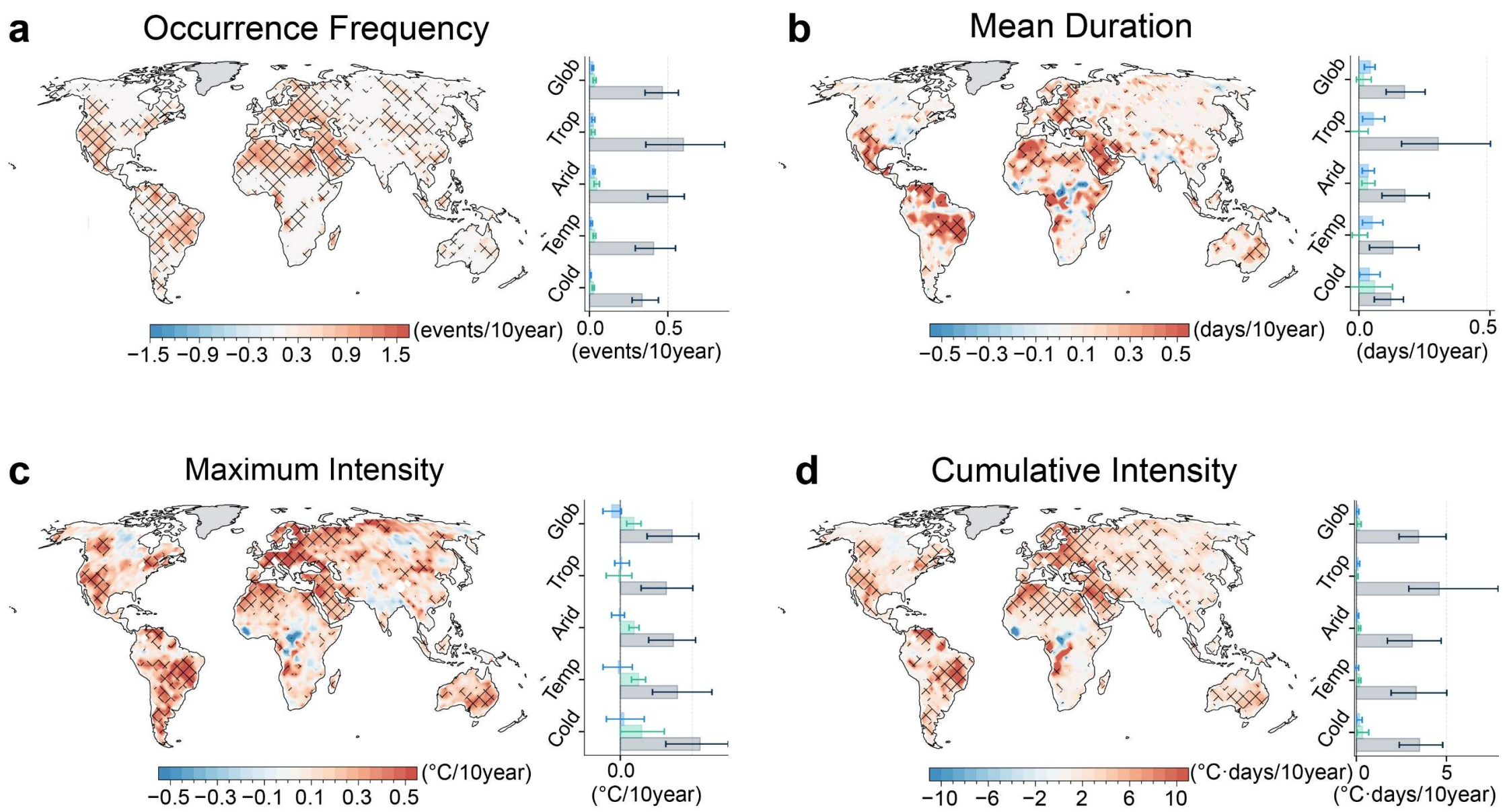

Supplementary Fig. 5 Observed historical trends of compound air-soil heatwaves during 1980–2024 based on the MERRA2 dataset. a–f, Spatial distribution of long-term trends in the occurrence frequency (**a**), mean duration (**b**), maximum intensity (**c**), and cumulative intensity (**d**) of compound air-soil heatwaves over global land areas from 1980 to 2024. The trends passing the 0.1 significance level based on the modified nonparametric Mann-Kendall test [27] are marked by cross-hatching. The bar charts accompanying the maps showing the decadal trends of independent air heatwaves (blue), independent soil heatwaves (green), and compound air-soil heatwaves (grey) averaged over global land areas and across different climate zones (Glob: global, Trop: tropical zone, Arid: arid zone, Temp: temperate zone, and Cold: cold zone). Error bars indicate the 95% confidence interval of the trends.

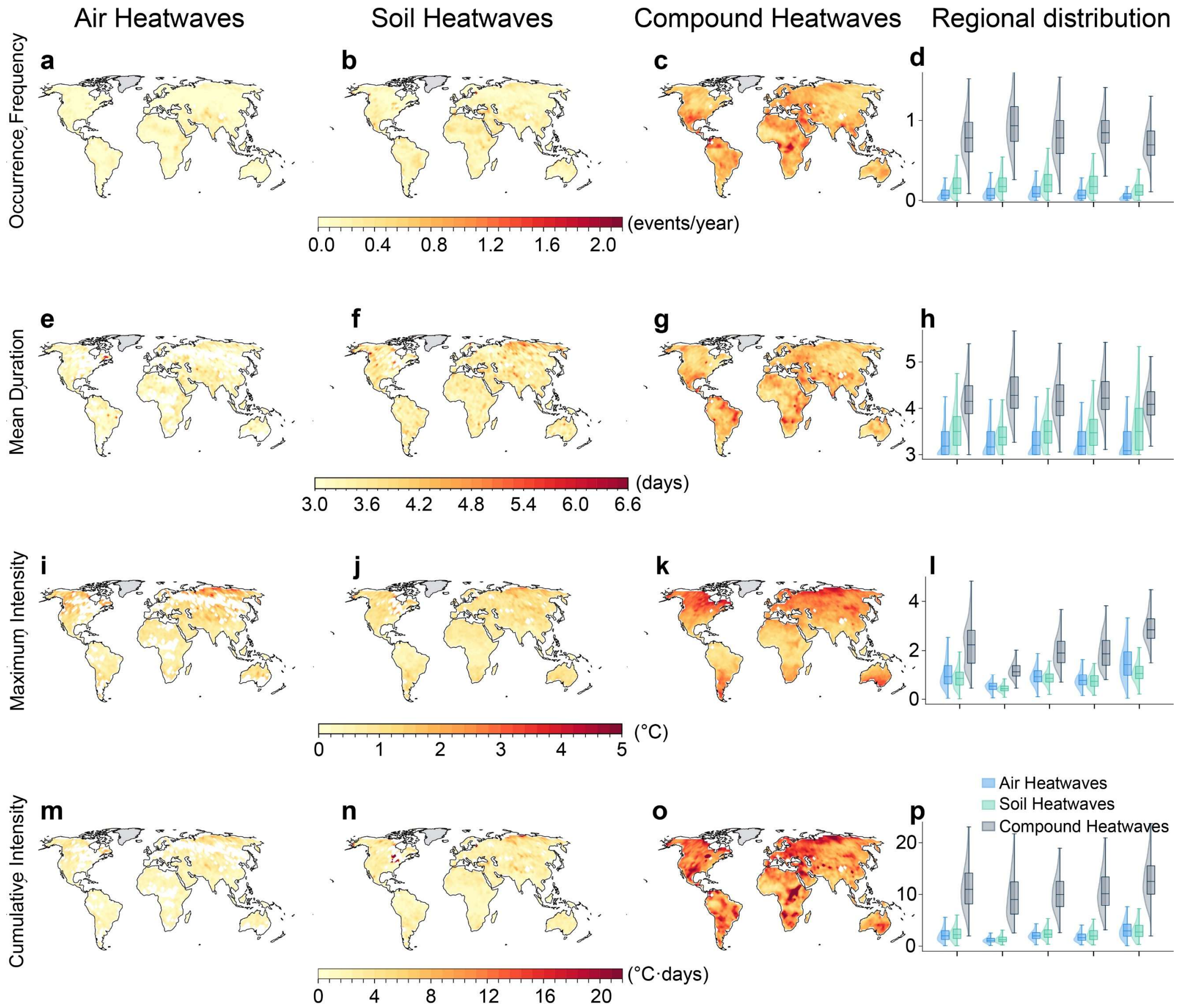

Supplementary Fig. 6 Observed climatology of independent and compound air-soil heatwaves based on the 95th percentile. a–w, Maps showing the climatological mean of annual mean occurrence frequency (**a–c**), mean duration (**e–g**), maximum intensity (**i–k**), and cumulative intensity (**m–o**) of independent air heatwaves (left column), independent soil heatwaves (middle column), and compound air-soil heatwaves (right column) over global land areas during 1979–2024. **d**, **h**, **l**, **p**, violin and box plots showing the distributions of climatological mean of the corresponding metrics of independent air heatwaves (blue), independent soil heatwaves (green), and compound air-soil heatwaves (grey) over global land areas and across different climate zones (Glob: global, Trop: tropical zone, Arid: arid zone, Temp: temperate zone, and Cold: cold zone). The centerline indicates the median value, the box bounds depict the 25th and 75th percentile values and the whiskers indicate the lower and upper quartiles±1.5 times the interquartile range of the values.

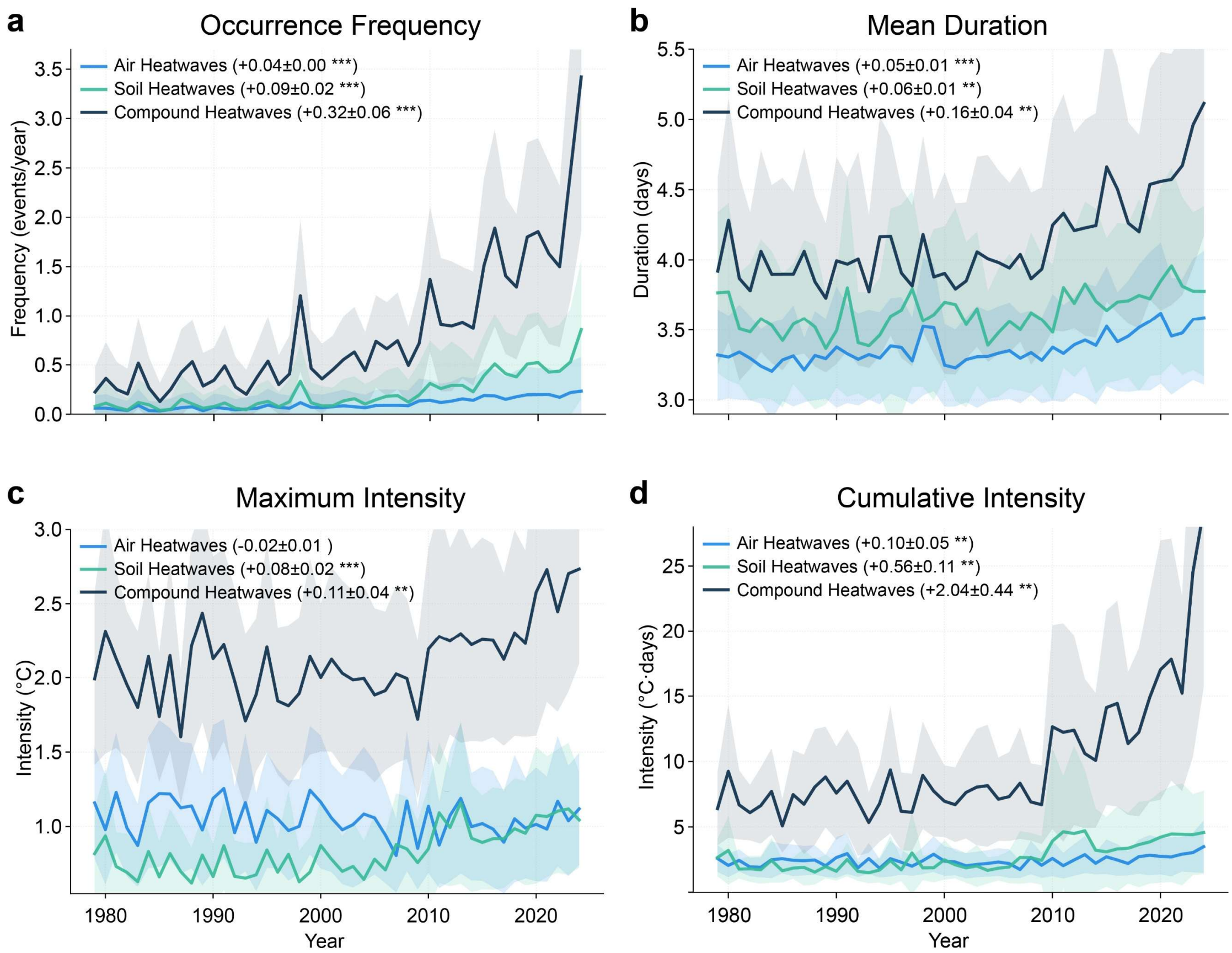

Supplementary Fig. 7 Long-term changes in independent and compound air-soil heatwaves based on the 95th percentile. a–f, Time series of the spatially averaged annual mean of occurrence frequency (**a**), mean duration (**b**), maximum intensity (**c**), and cumulative intensity (**d**) of independent air heatwaves (blue), independent soil heatwaves (green), and compound air-soil heatwaves (grey) over global land areas from 1979 to 2024. Solid line represents the time series of regional mean and the shaded regions represent half of the standard deviation. Slope estimates for the trend per decade are given in parentheses. *$p < 0.05$ for long-term trend; **$p < 0.01$; ***$p < 0.001$.

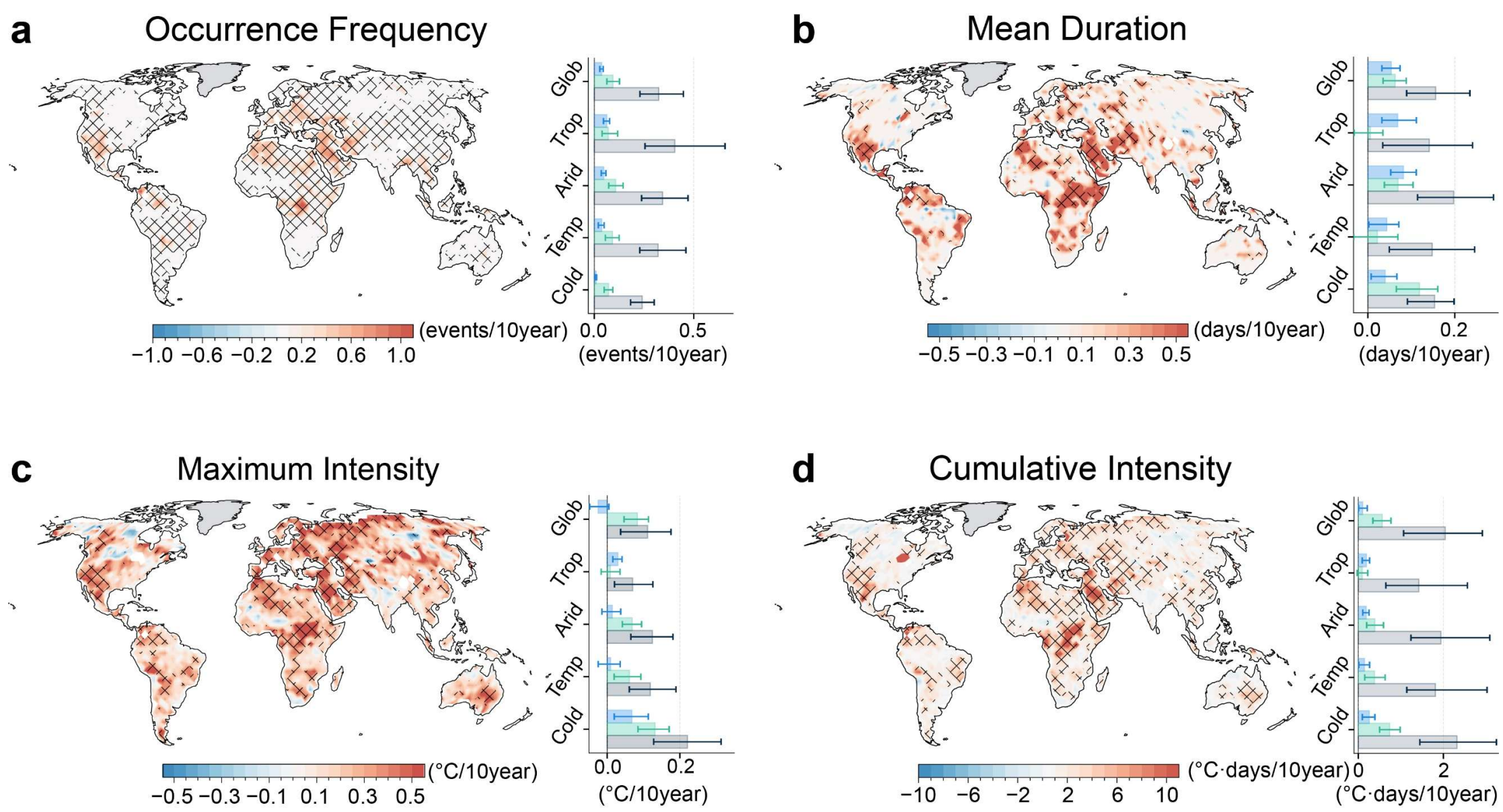

Supplementary Fig. 8 Observed historical trends of compound air-soil heatwaves based on the 95th percentile. a–f, Spatial distribution of long-term trends in the occurrence frequency (**a**), mean duration (**b**), maximum intensity (**c**), and cumulative intensity (**d**) of compound air-soil heatwaves over global land areas from 1979 to 2024. The trends passing the 0.1 significance level based on the modified nonparametric Mann-Kendall test[27] are marked by cross-hatching. The bar charts accompanying the maps showing the decadal trends of independent air heatwaves (blue), independent soil heatwaves (green), and compound air-soil heatwaves (grey) averaged over global land areas and across different climate zones (Glob: global, Trop: tropical zone, Arid: arid zone, Temp: temperate zone, and Cold: cold zone). Error bars indicate the 95% confidence interval of the trends.

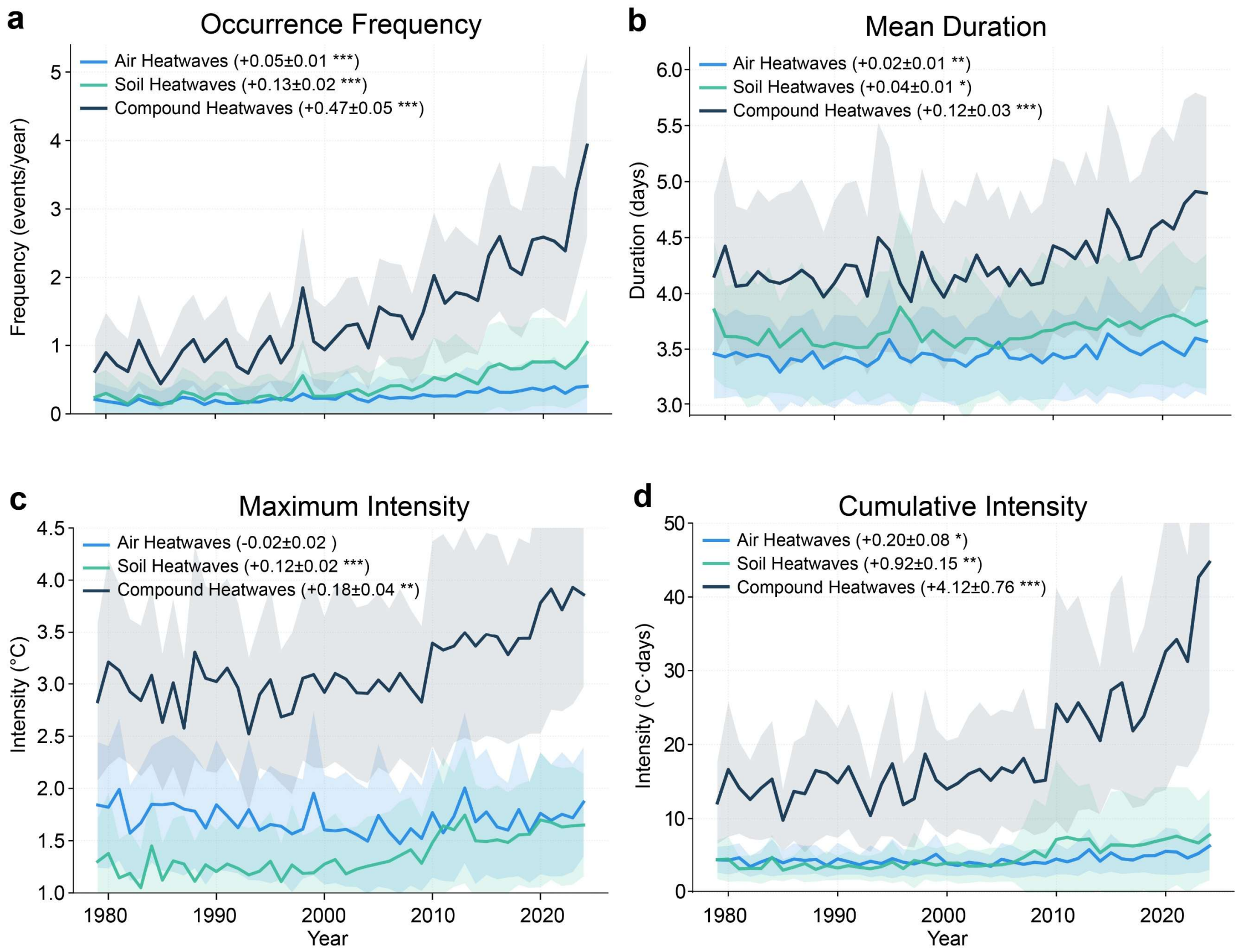

Supplementary Fig. 9 Long-term changes in independent and compound air-soil heatwaves based on the maximum air temperature and soil temperature. a–f, Time series of the spatially averaged annual mean of occurrence frequency (**a**), mean duration (**b**), maximum intensity (**c**), and cumulative intensity (**d**) of independent air heatwaves (blue), independent soil heatwaves (green), and compound air-soil heatwaves (grey) over global land areas from 1979 to 2024. Solid line represents the time series of regional mean and the shaded regions represent half of the standard deviation. Slope estimates for the trend per decade are given in parentheses. *$p < 0.05$ for long-term trend; **$p < 0.01$; ***$p < 0.001$.

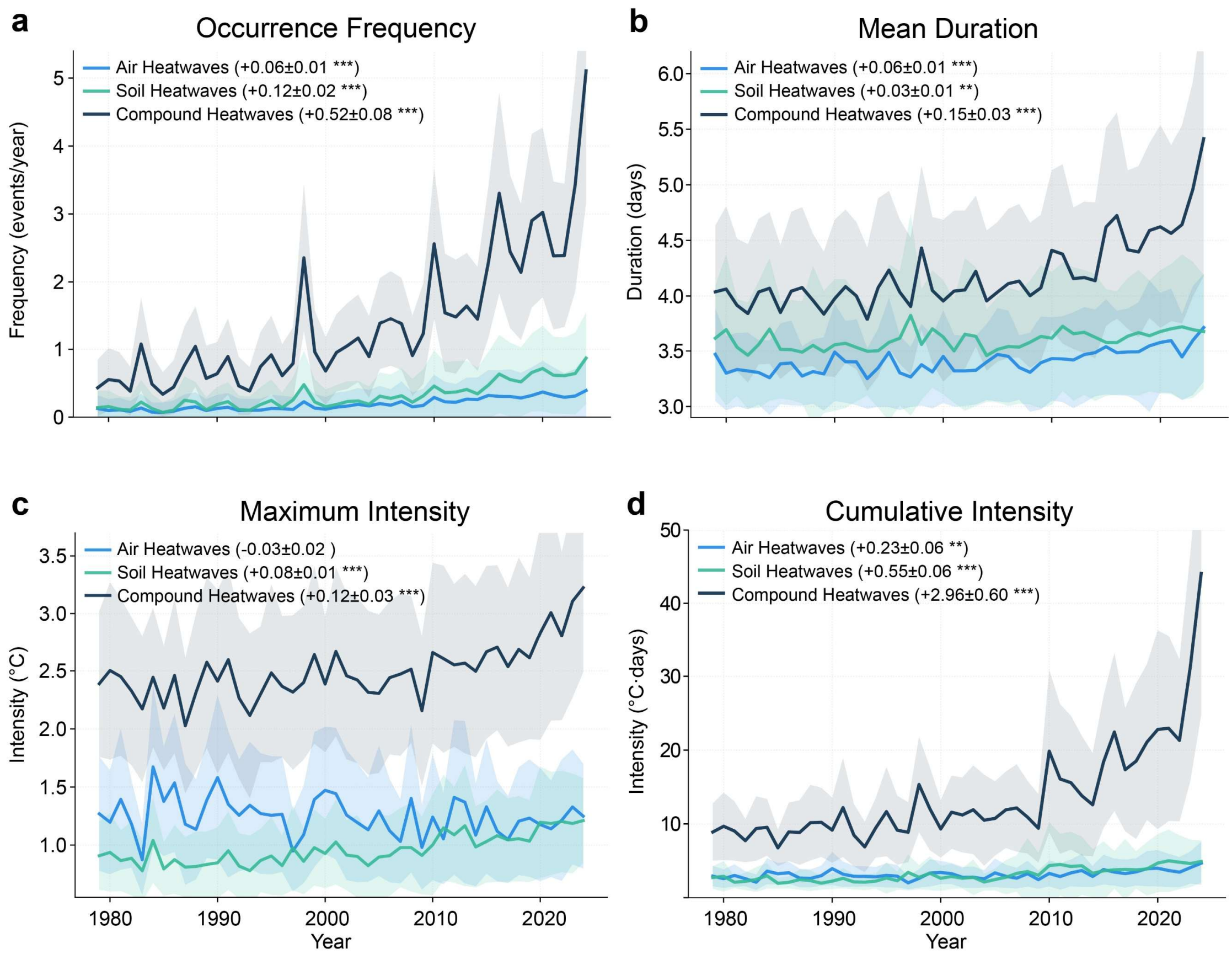

Supplementary Fig. 10 Long-term changes in independent and compound air-soil heatwaves based on the minimum air temperature and soil temperature. a–f, Time series of the spatially averaged annual mean of occurrence frequency (**a**), mean duration (**b**), maximum intensity (**c**), and cumulative intensity (**d**) of independent air heatwaves (blue), independent soil heatwaves (green), and compound air-soil heatwaves (grey) over global land areas from 1979 to 2024. Solid line represents the time series of regional mean and the shaded regions represent half of the standard deviation. Slope estimates for the trend per decade are given in parentheses. *$p < 0.05$ for long-term trend; **$p < 0.01$; ***$p < 0.001$.

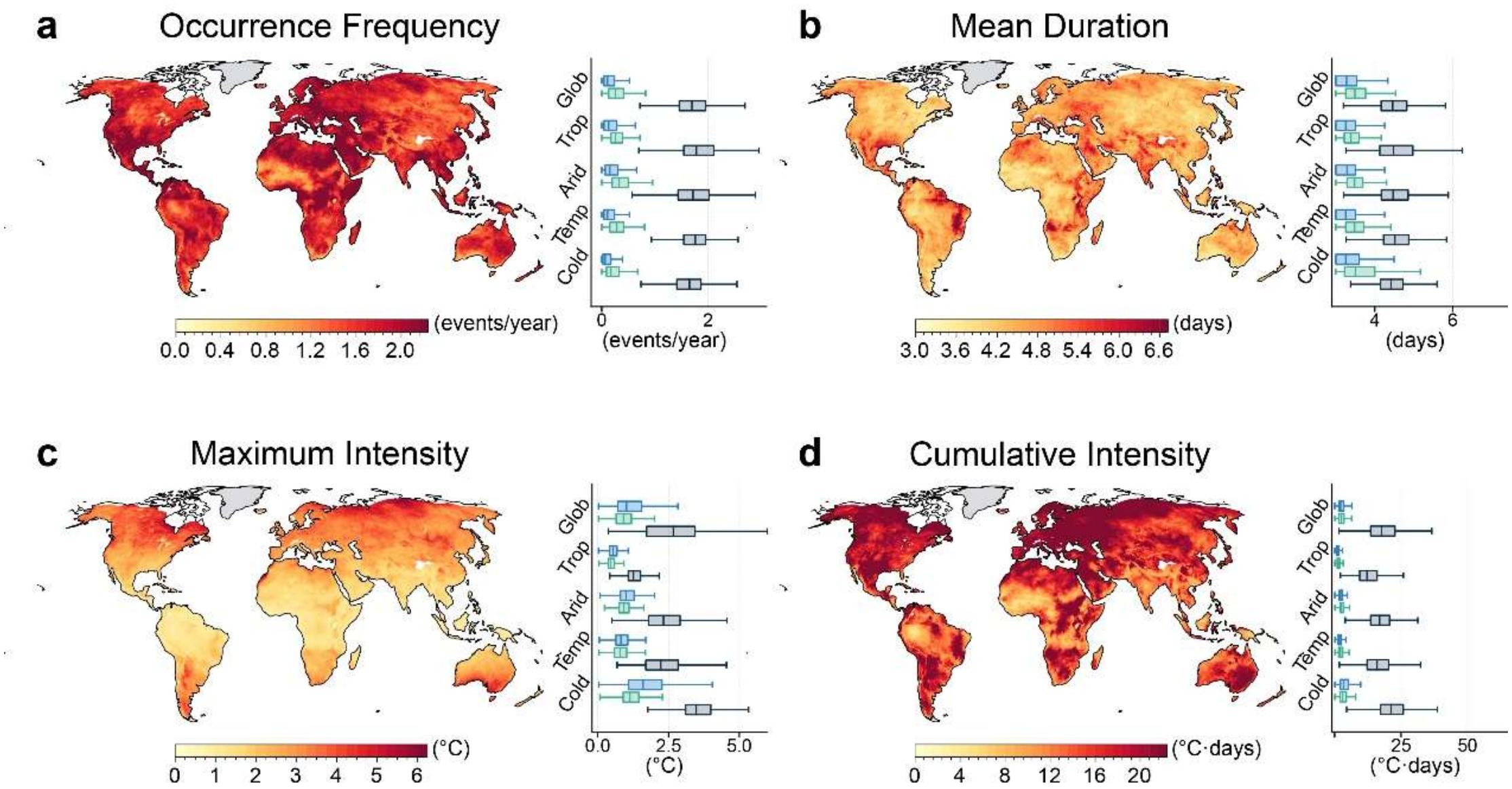

Supplementary Fig. 11 Observed climatology of compound air-soil heatwaves at the 0.25°×0.25° resolution. a–f, Spatial distribution of the climatological mean of annual mean occurrence frequency (**a**), mean duration (**b**), maximum intensity (**c**), and cumulative intensity (**d**), of compound air-soil heatwaves over global land areas from 1979 to 2024. The box plots charts accompanying the maps showing the distributions of climatological mean of the corresponding metrics of independent air heatwaves (blue), independent soil heatwaves (green), and compound air-soil heatwaves (grey) averaged over global land areas and across different climate zones (Glob: global, Trop: tropical zone, Arid: arid zone, Temp: temperate zone, and Cold: cold zone). The centerline indicates the median value, the box bounds depict the 25th and 75th percentile values and the whiskers indicate the lower and upper quartiles±1.5 times the interquartile range of the values.

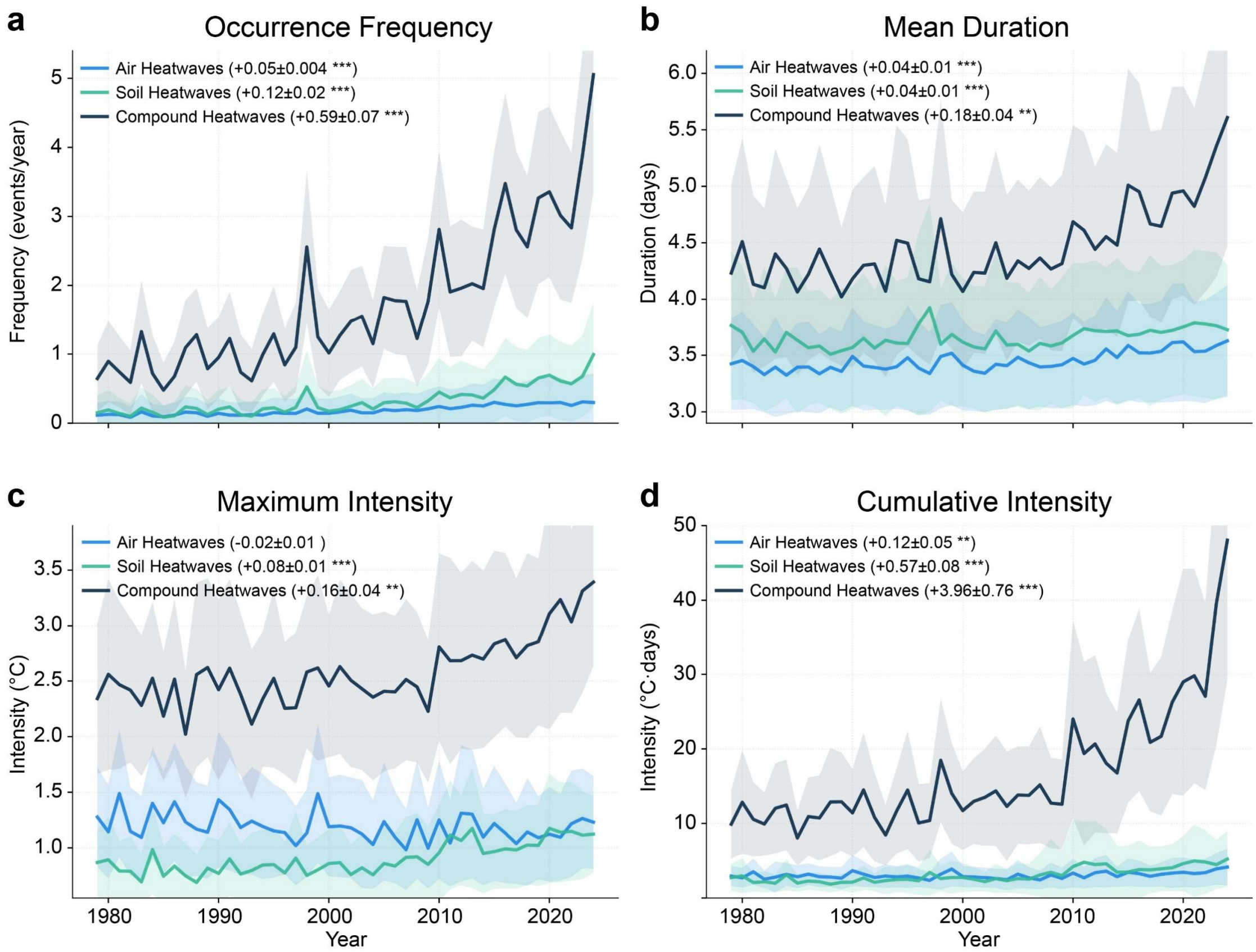

Supplementary Fig. 12 Long-term changes in independent and compound air-soil heatwaves at the 0.25°×0.25° resolution. a–f, Time series of the spatially averaged annual mean of occurrence frequency (**a**), mean duration (**b**), maximum intensity (**c**), and cumulative intensity (**d**) of independent air heatwaves (blue), independent soil heatwaves (green), and compound air-soil heatwaves (grey) over global land areas from 1979 to 2024. Solid line represents the time series of regional mean and the shaded regions represent half of the standard deviation. Slope estimates for the trend per decade are given in parentheses. *$p < 0.05$ for long-term trend; **$p < 0.01$; ***$p < 0.001$.

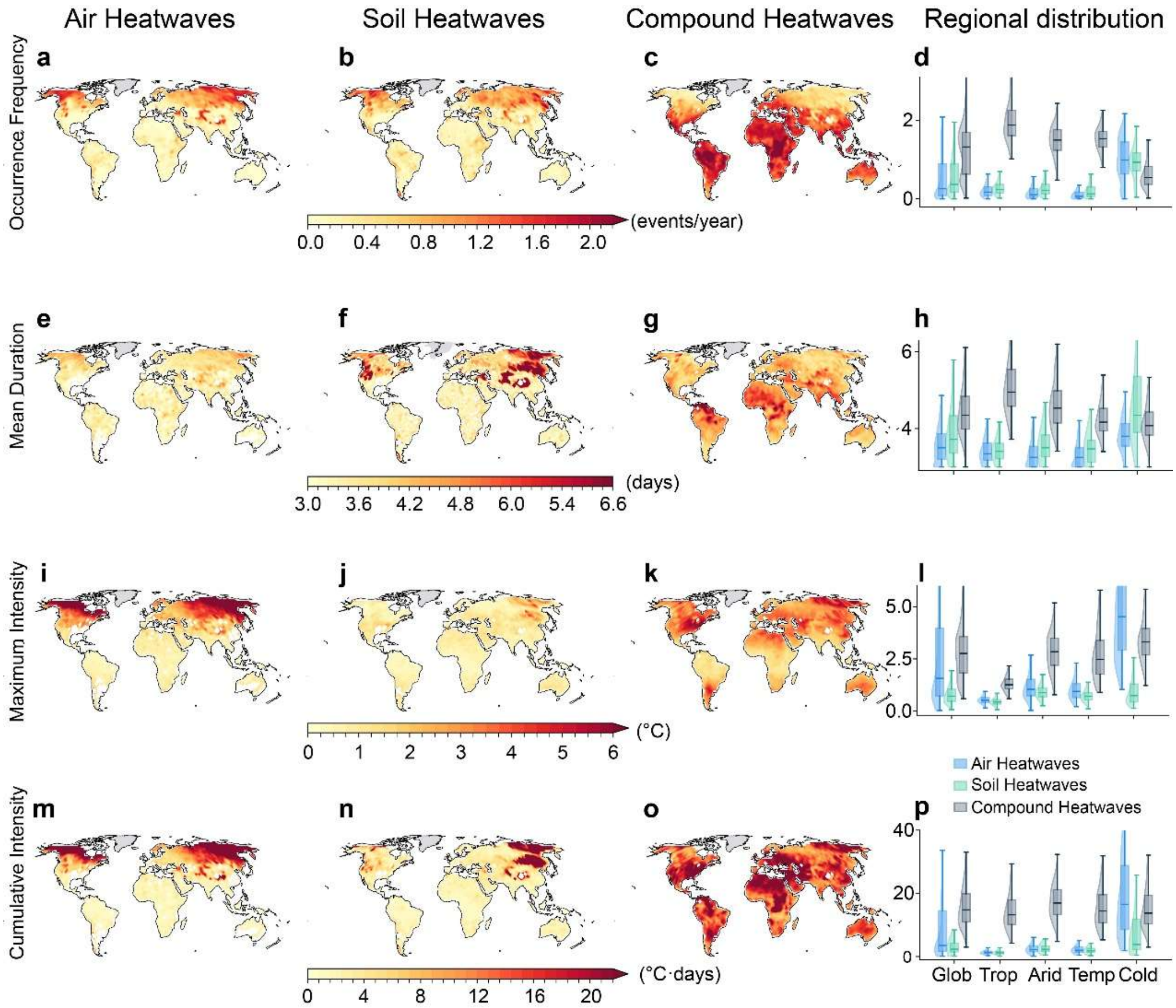

Supplementary Fig. 13 Observed climatology of independent and compound air-soil heatwaves during the cold season. a–w, Maps showing the climatological mean of annual mean occurrence frequency (**a–c**), mean duration (**e–g**), maximum intensity (**i–k**), and cumulative intensity (**m–o**) of independent air heatwaves (left column), independent soil heatwaves (middle column), and compound air-soil heatwaves (right column) over global land areas during cold season during 1979–2024. **d**, **h**, **l**, **p** violin and box plots showing the distributions of climatological mean of the corresponding metrics of independent air heatwaves (blue), independent soil heatwaves (green), and compound air-soil heatwaves (grey) over global land areas and across different climate zones (Glob: global, Trop: tropical zone, Arid: arid zone, Temp: temperate zone, and Cold: cold zone). The centerline indicates the median value, the box bounds depict the 25th and 75th percentile values and the whiskers indicate the lower and upper quartiles±1.5 times the interquartile range of the values. The cold season is defined as November–March in the Northern Hemisphere and May–September in the Southern Hemisphere.

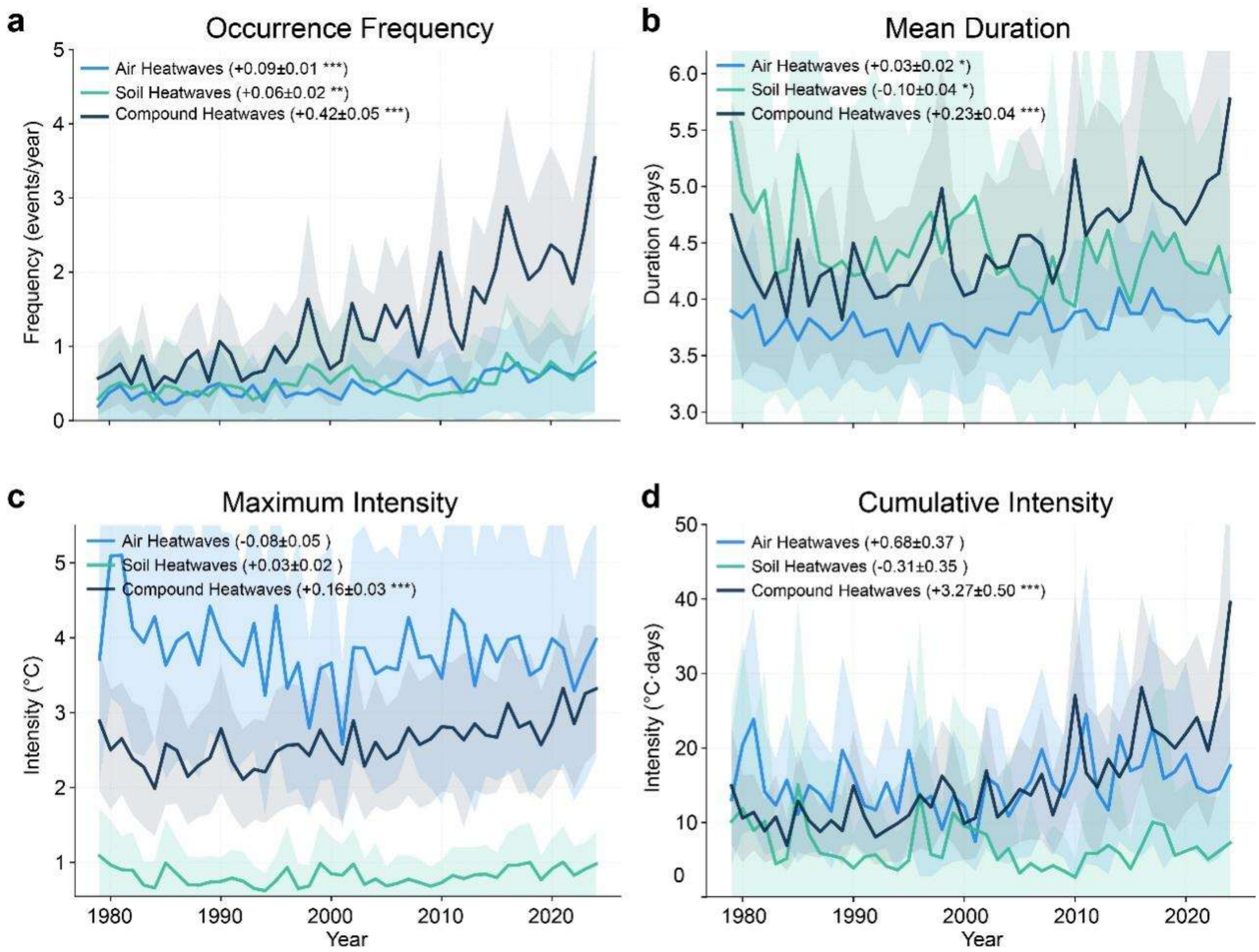

Supplementary Fig. 14 Long-term changes in independent and compound air-soil heatwaves during the cold season. a–f, Time series of the spatially averaged annual mean of occurrence frequency (**a**), mean duration (**b**), maximum intensity (**c**), and cumulative intensity (**d**) of independent air heatwaves (blue), independent soil heatwaves (green), and compound air-soil heatwaves (grey) over global land areas during cold season from 1979 to 2024. Solid line represents the time series of regional mean and the shaded regions represent half of the standard deviation. Slope estimates for the trend per decade are given in parentheses. *$p < 0.05$ for long-term trend; **$p < 0.01$; ***$p < 0.001$. The cold season is defined as November–March in the Northern Hemisphere and May–September in the Southern Hemisphere.

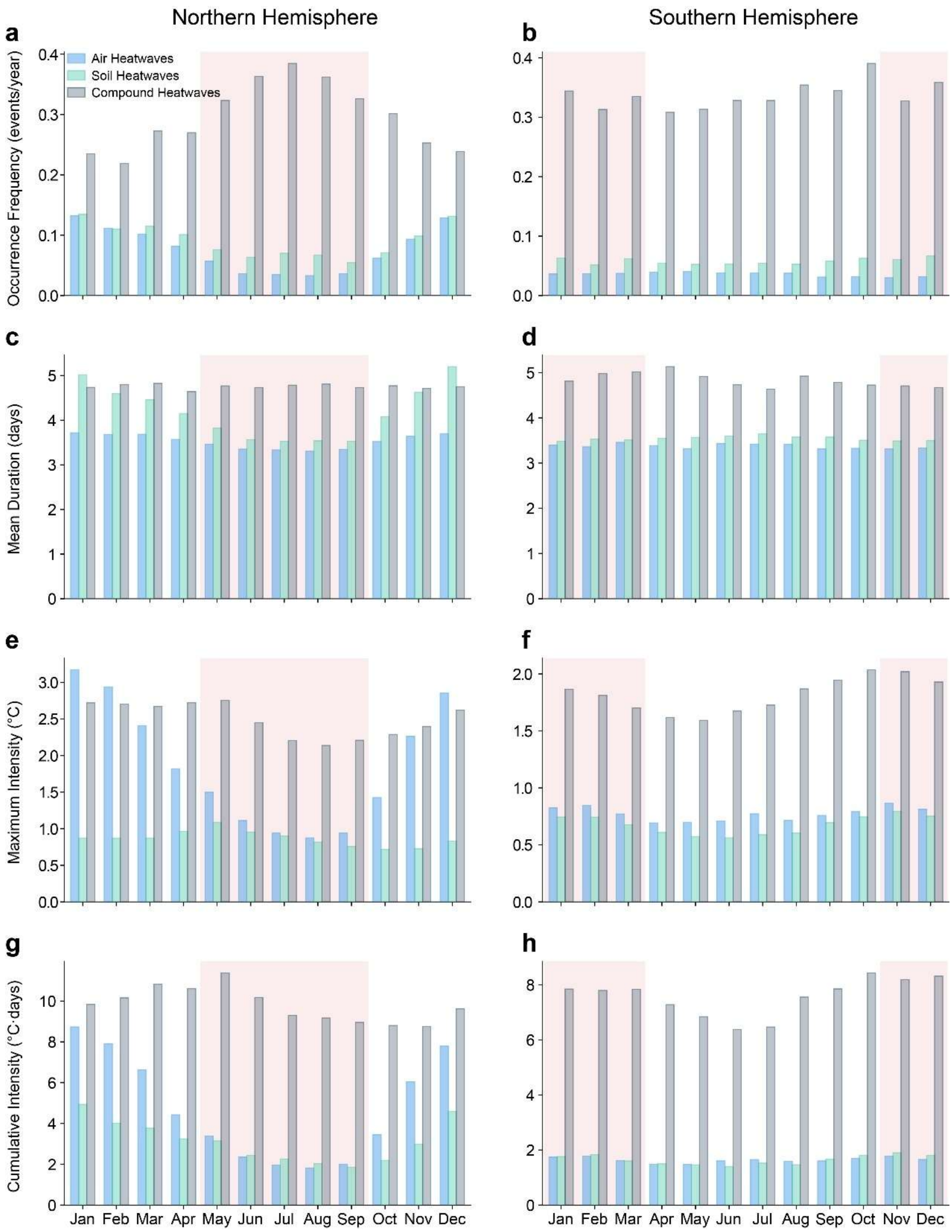

Supplementary Fig. 15 Observed year-round monthly climatology of independent and compound air-soil heatwaves. a–h, Bar charts showing the climatological mean of occurrence frequency (**a, b**), mean duration (**c, d**), maximum intensity (**e, f**), and cumulative intensity (**g, h**) of independent air heatwaves (blue), independent soil heatwaves (green), and compound air-soil heatwaves (grey) for the Northern Hemisphere (left column) and Southern Hemisphere (right column) during 1979–2024. The shaded areas indicate the warm season months (defined as May–September in the Northern Hemisphere and November–March in the Southern Hemisphere).

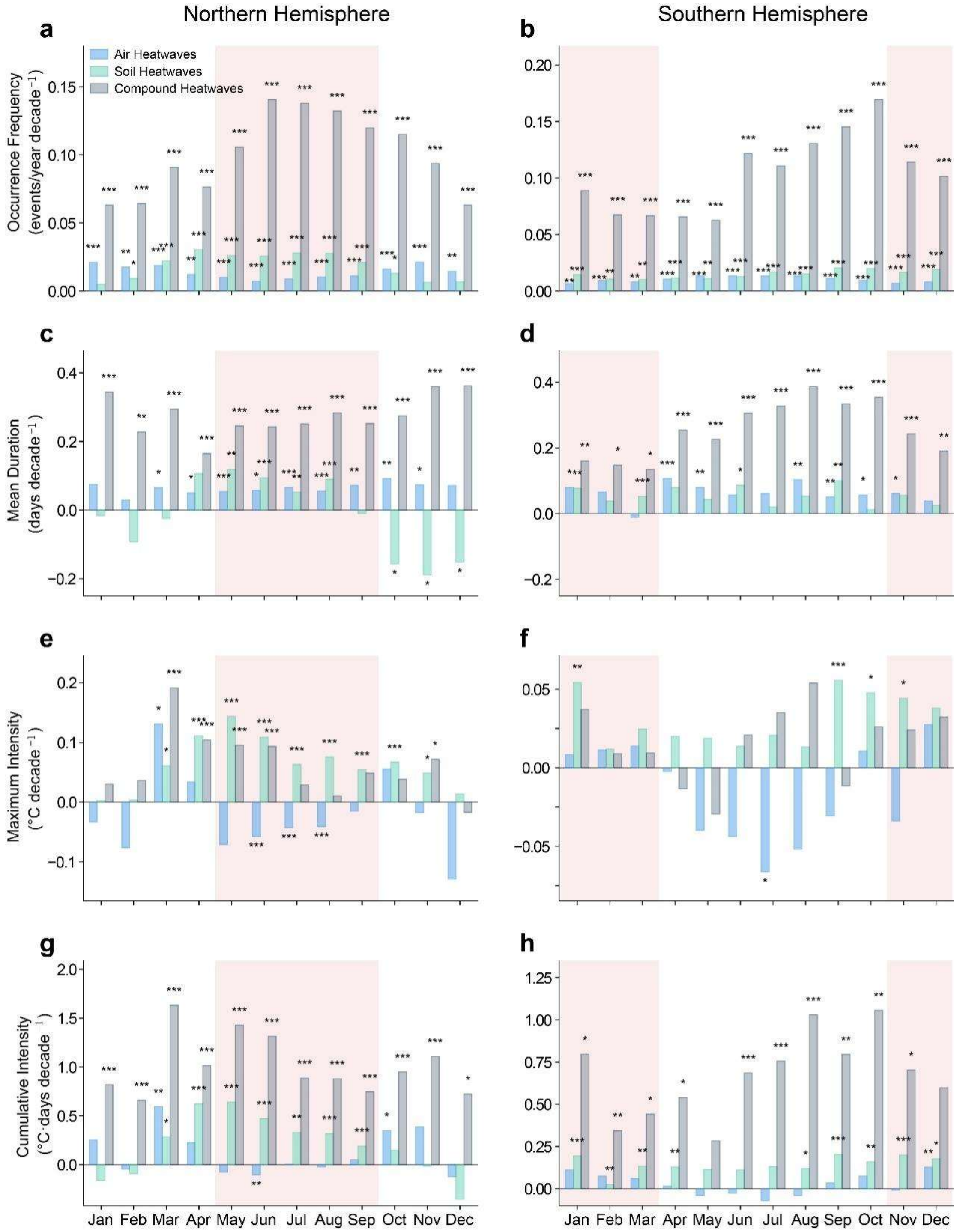

Supplementary Fig. 16 Long-term trends of year-round monthly independent and compound air-soil heatwaves. a–h, Bar charts showing the decadal trends of occurrence frequency (**a, b**), mean duration (**c, d**), maximum intensity (**e, f**), and cumulative intensity (**g, h**) of independent air heatwaves (blue), independent soil heatwaves (green), and compound air-soil heatwaves (grey) for the Northern Hemisphere (left column) and Southern Hemisphere (right column) during 1979–2024. *$p < 0.05$ for long-term trend; **$p < 0.01$; ***$p < 0.001$. The shaded areas indicate the

warm-season months, defined as May–September in the Northern Hemisphere and November–March in the Southern Hemisphere.

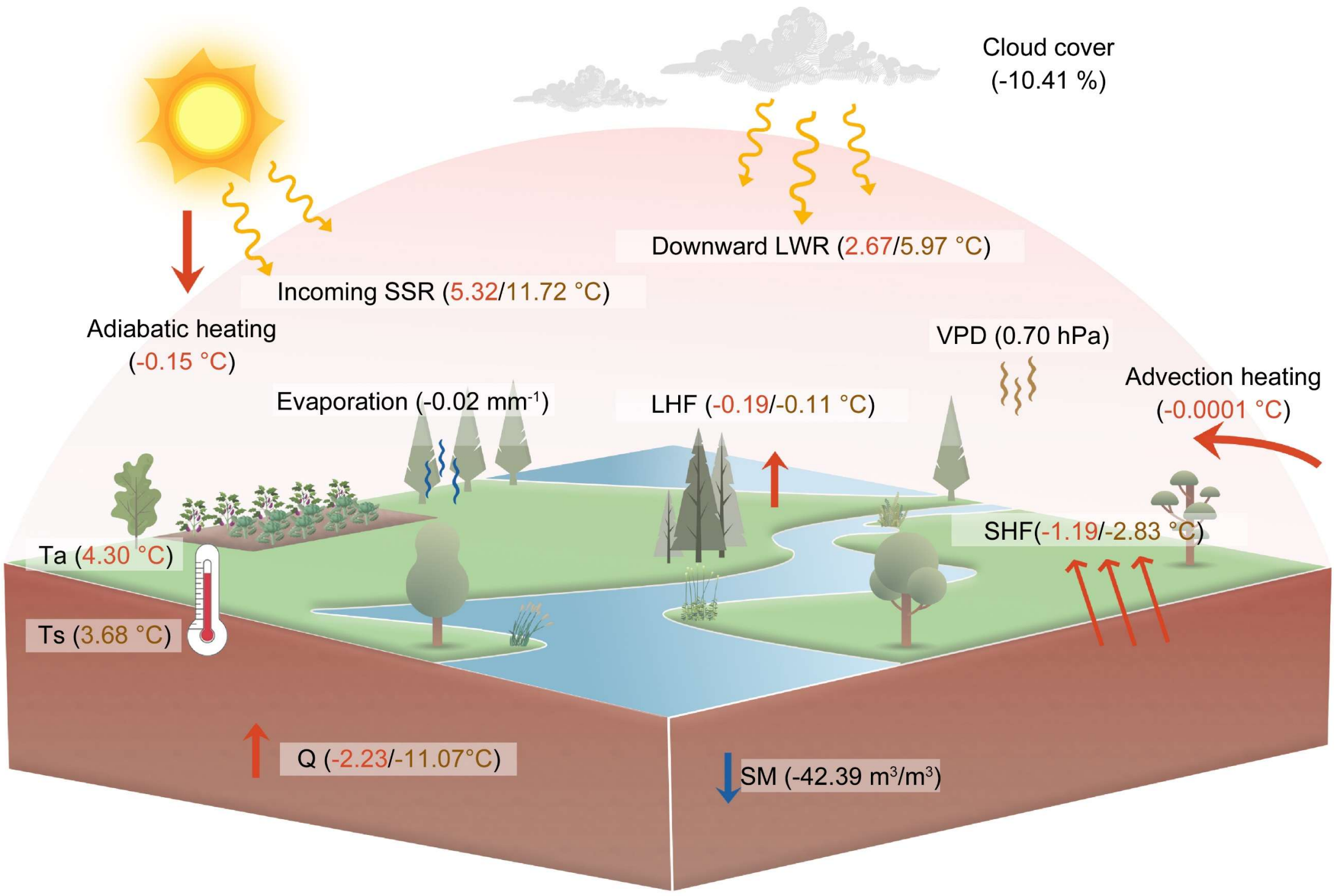

Supplementary Fig. 17 Conceptual diagram illustrating physical processes during compound air-soil heatwaves. Red labels denote the regional mean of the decomposed contributions to the air temperature anomalies; brown labels denote the regional mean of the decomposed contributions to the soil temperature anomalies; and black labels denote the regional mean of the anomalies of relevant variables (e.g., cloud cover, vapor pressure deficit, evaporation, and soil moisture) associated with the compound heatwave events.

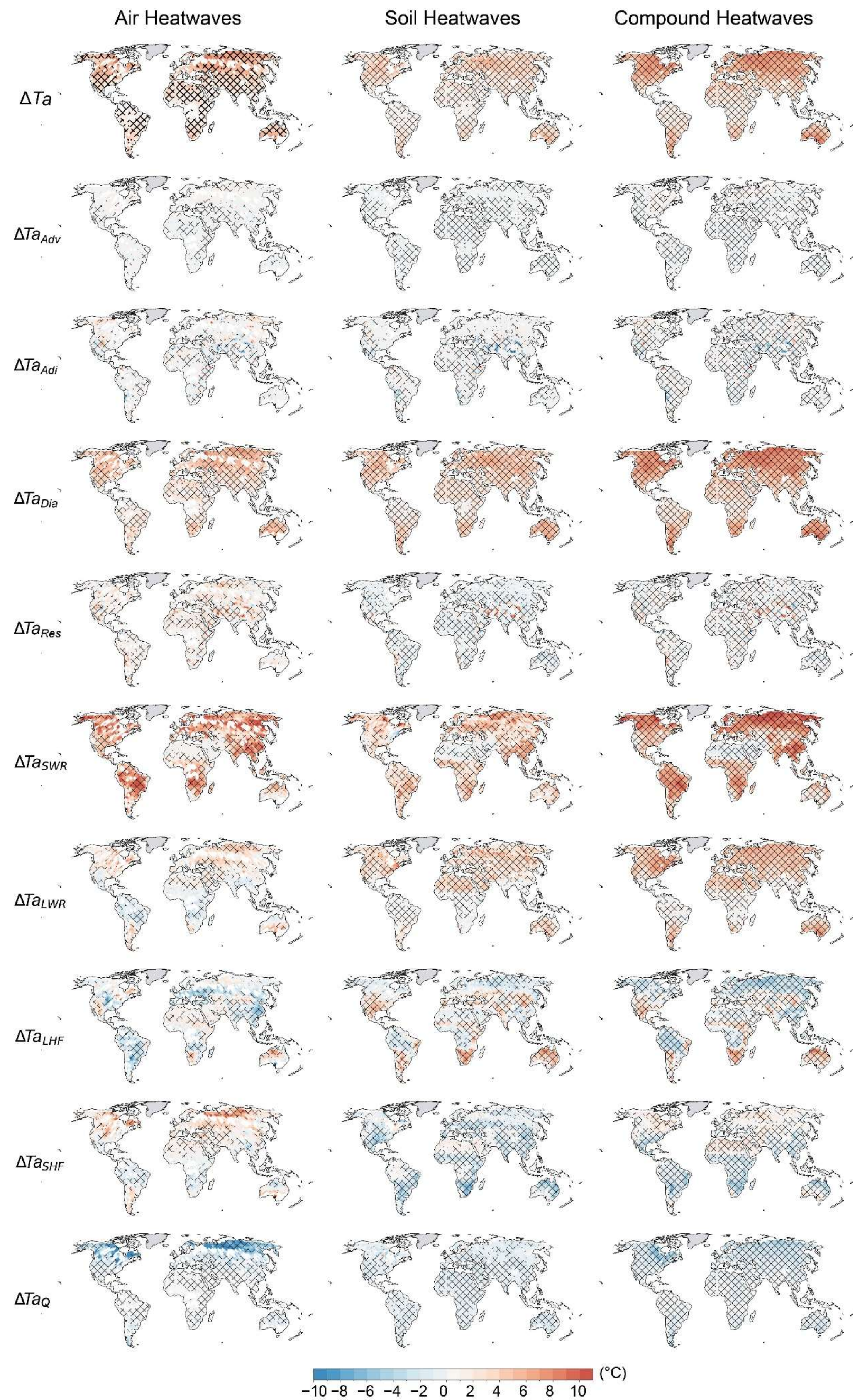
Air Heatwaves
Soil Heatwaves
Compound Heatwaves
ΔTa
ΔTa_Adv
ΔTa_Adi
ΔTa_Dia
ΔTa_Res
ΔTa_SWR
ΔTa_LWR
ΔTa_LHF
ΔTa_SHF
ΔTa_Q
(°C)
−10 −8 −6 −4 −2 0 2 4 6 8 10

Supplementary Fig. 18 Diagnostic decomposition of air temperature anomalies during independent and compound air-soil heatwaves. Composite near-surface air temperature anomalies associated with independent air heatwaves (ΔTa, top left), independent soil heatwaves (top middle), compound air-soil heatwaves (top right) over global land areas from 1979 to 2024, and their decomposition into the contributions of advection heating (ΔTa_{ADV}), adiabatic heating (ΔTa_{ADI}), and diabatic heating (ΔTa_{DIA}). The decomposition of the diabatic heating term into the contributions from net surface shortwave radiation (ΔTa_{SWR}), surface downward longwave radiation (ΔTa_{LWR}), surface latent heat flux (ΔTa_{LHF}), surface sensible heat flux (ΔTa_{SHF}), and ground heat flux (ΔTa_{Q}). Regions where the composite mean passes the 0.1 significance level by the Student's *t*-test are marked by cross-hatching.

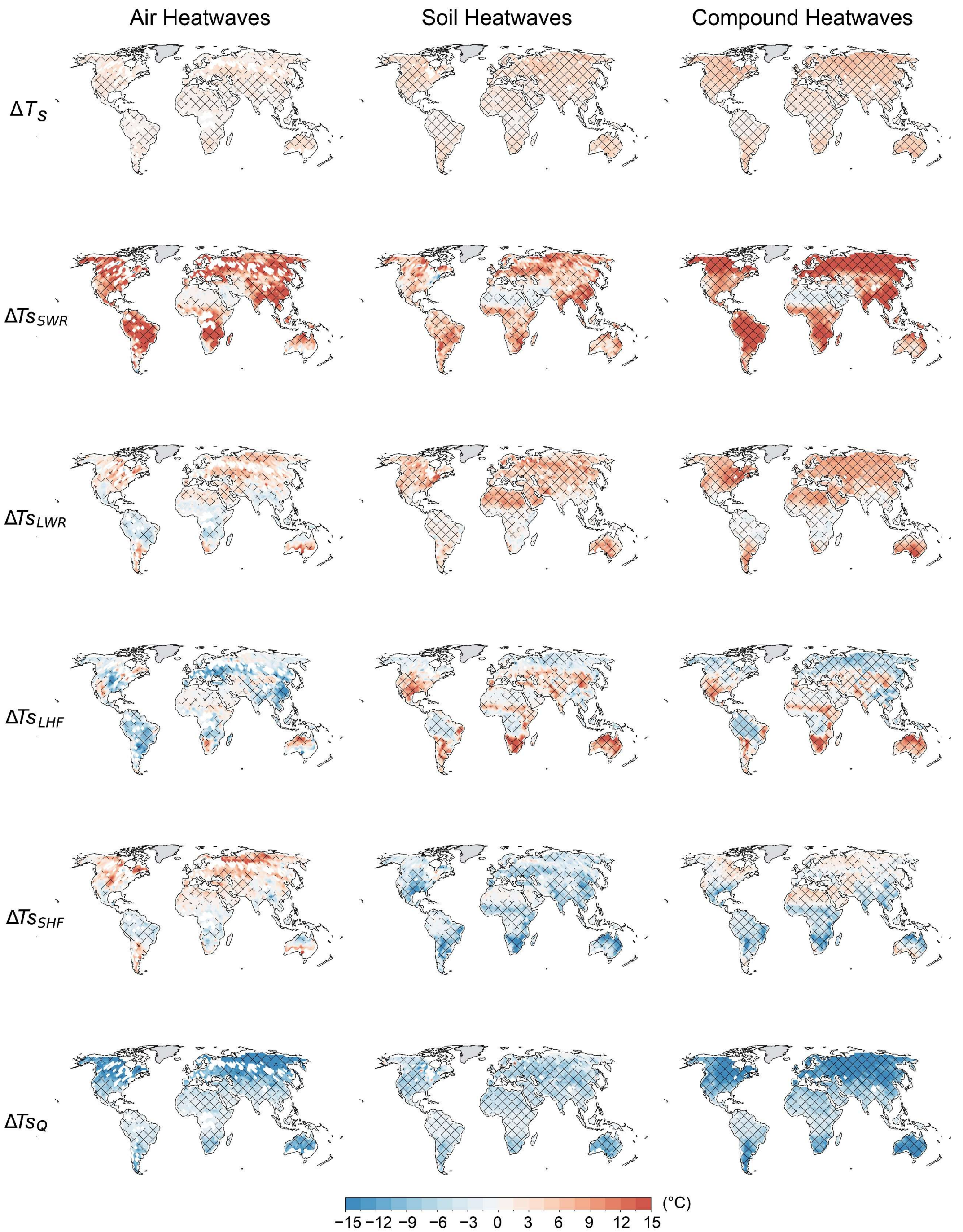

Supplementary Fig. 19 Diagnostic decomposition of soil temperature anomalies during independent and compound air-soil heatwaves. Composite soil temperature anomalies associated with independent air heatwaves (ΔTs, top left), independent soil heatwaves (top middle), compound air-soil heatwaves (top right) over global land areas from 1979 to 2024, and their decomposition) into the contributions from net surface shortwave radiation (ΔTs_{SWR}), surface downward longwave radiation (ΔTs_{LWR}), surface latent heat flux (ΔTs_{LHF}), surface sensible heat flux (ΔTs_{SHF}), and ground heat flux (ΔTs_Q). Regions where the composite mean passes the 0.1 significance level by the Student's *t*-test are marked by cross-hatching.

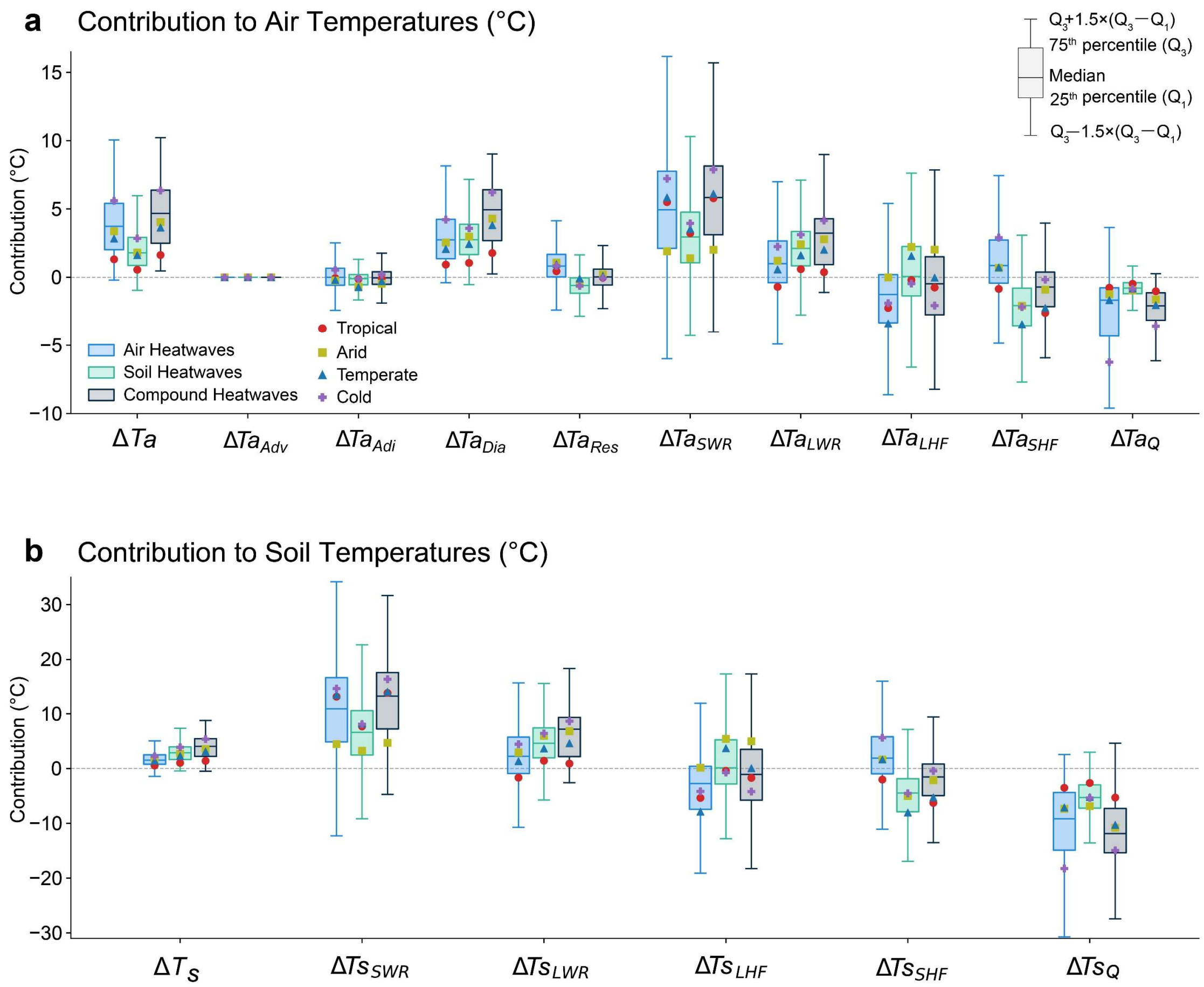

Supplementary Fig. 20 Diagnostic decomposition of temperature anomalies during independent and compound air-soil heatwaves. a, Boxplot showing the regional mean of the air temperature anomalies and its decomposition into the contribution of different heating terms to independent air heatwaves (blue), independent soil heatwaves (green), and compound air-soil heatwaves (grey) over global land areas from 1979 to 2024. The superimposed makers denote the regional mean of the temperature anomalies and their decomposed heating contributions across different climate zones. The centerline indicates the median value, the box bounds depict the 25th and 75th percentile values and the whiskers indicate the lower and upper quartiles±1.5 times the interquartile range of the decomposition of temperature anomalies. **b**, As **a** but for decomposition of soil temperature anomalies.

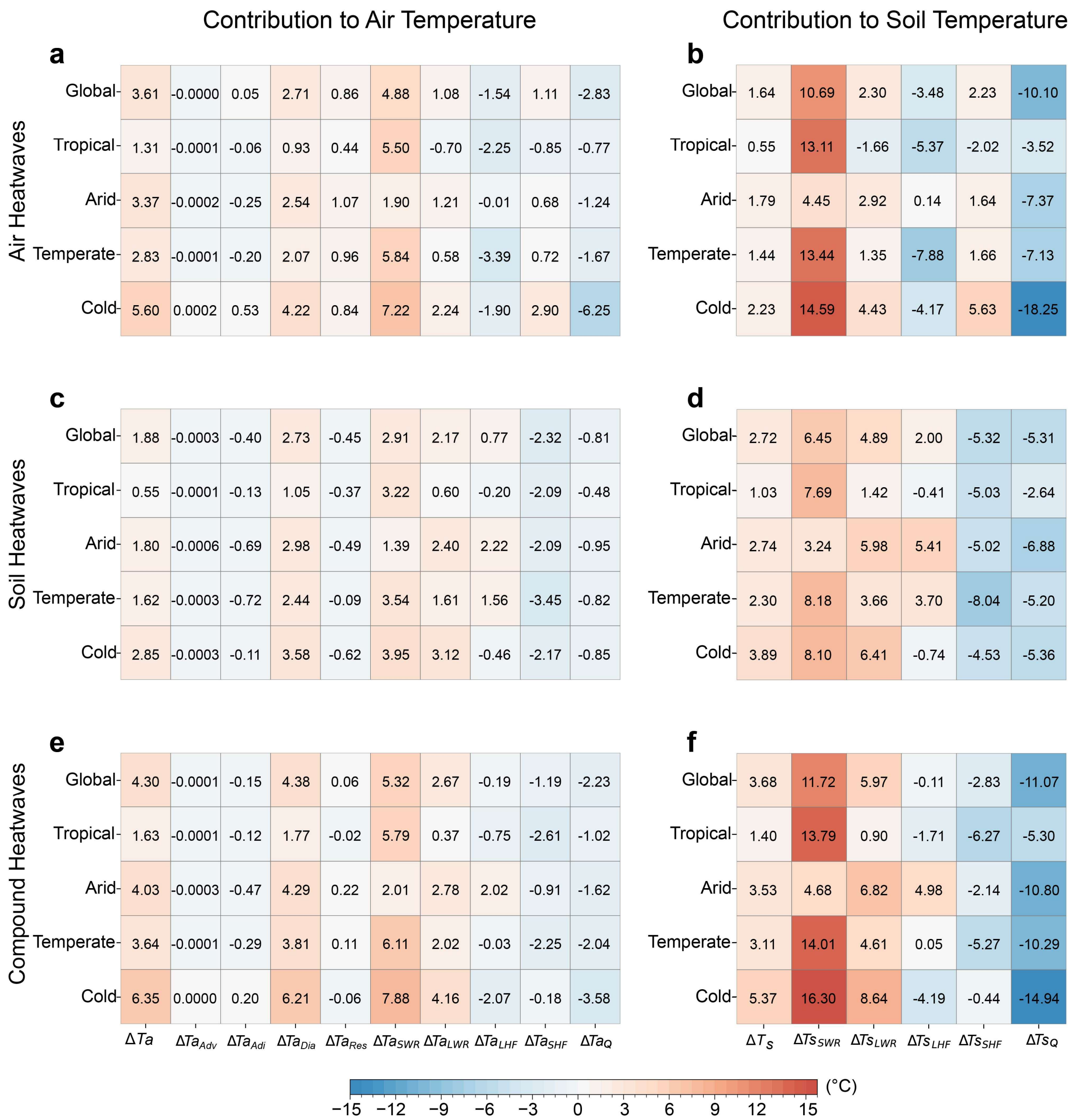

Supplementary Fig. 21 Regional mean of the diagnostic contributions to temperature anomalies during independent and compound air-soil heatwaves. a, c, e, The regional mean of the air temperature anomalies and its decomposition into the contribution of different heating terms to independent air heatwaves (**a**), independent soil heatwaves (**c**), and compound air-soil heatwaves (**e**) over global land areas from 1979 to 2024. **b**, **d**, **f**, As **a**, **c**, **e** but for decomposition of soil temperature anomalies.

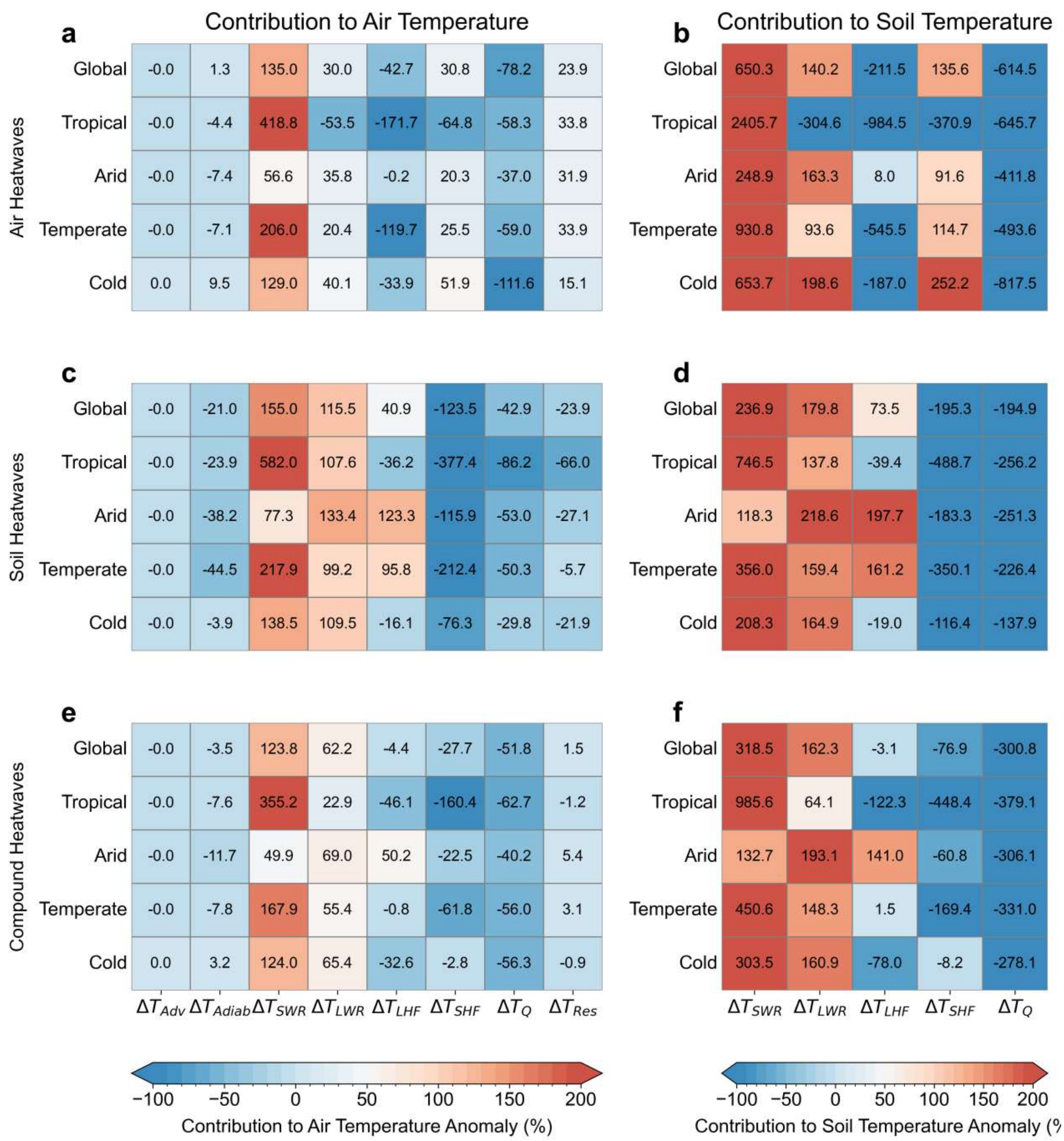

Supplementary Fig. 22 Regional proportional diagnostic contributions to temperature anomalies during independent and compound air-soil heatwaves. a, c, e, Regional proportional contributions of different heating terms to near-surface air temperature anomalies during independent air heatwaves (**a**), independent soil heatwaves (**c**), and compound air-soil heatwaves (**e**) over global land areas and four climate zones. **b, d, f,** As in **a, c, e**, but for shallow soil temperature anomalies. Contributions are expressed as percentages of the corresponding regional mean temperature anomaly. Positive values indicate processes that enhance warming, whereas negative values indicate processes that offset warming. Individual contributions may exceed 100% because the observed temperature anomaly represents the net result of compensating positive and negative terms.

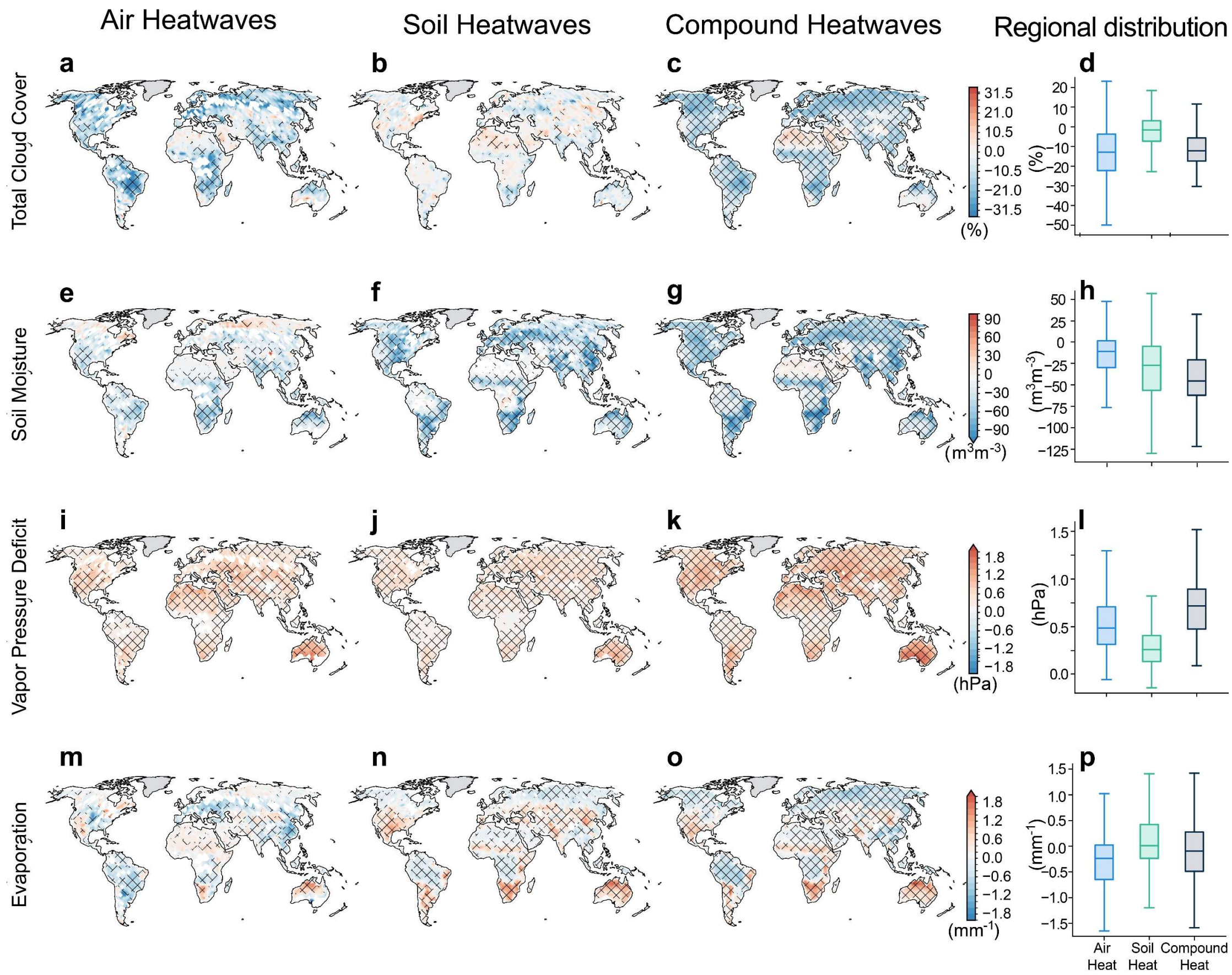

Supplementary Fig. 23 Composite anomalies of key variables associated with independent and compound air-soil heatwaves. The composite mean anomalies of total cloud cover (**a**–**c**), soil moisture (**e**–**g**), vapor pressure deficit (**i**–**k**), and evaporation (**m**–**o**) during independent air heatwaves (**a, e, i, m**), independent soil heatwaves (**b, f, j, n**), and compound air-soil heatwaves (**c, g, k, o**). Evaporation is positive downwards. Regions where the composite mean passes the 0.1 significance level by the Student's *t*-test are marked by cross-hatching. **d, h, l, p** The boxplot chart in right panel shows the regional mean of the anomalies of corresponding variables associated with independent air heatwaves (blue), independent soil heatwaves (green), and compound air-soil heatwaves (grey) over global land areas. The centerline indicates the median value, the box bounds depict the 25th and 75th percentile values, and the whiskers indicate the lower and upper quartiles±1.5 times the interquartile range of the composite mean anomalies.

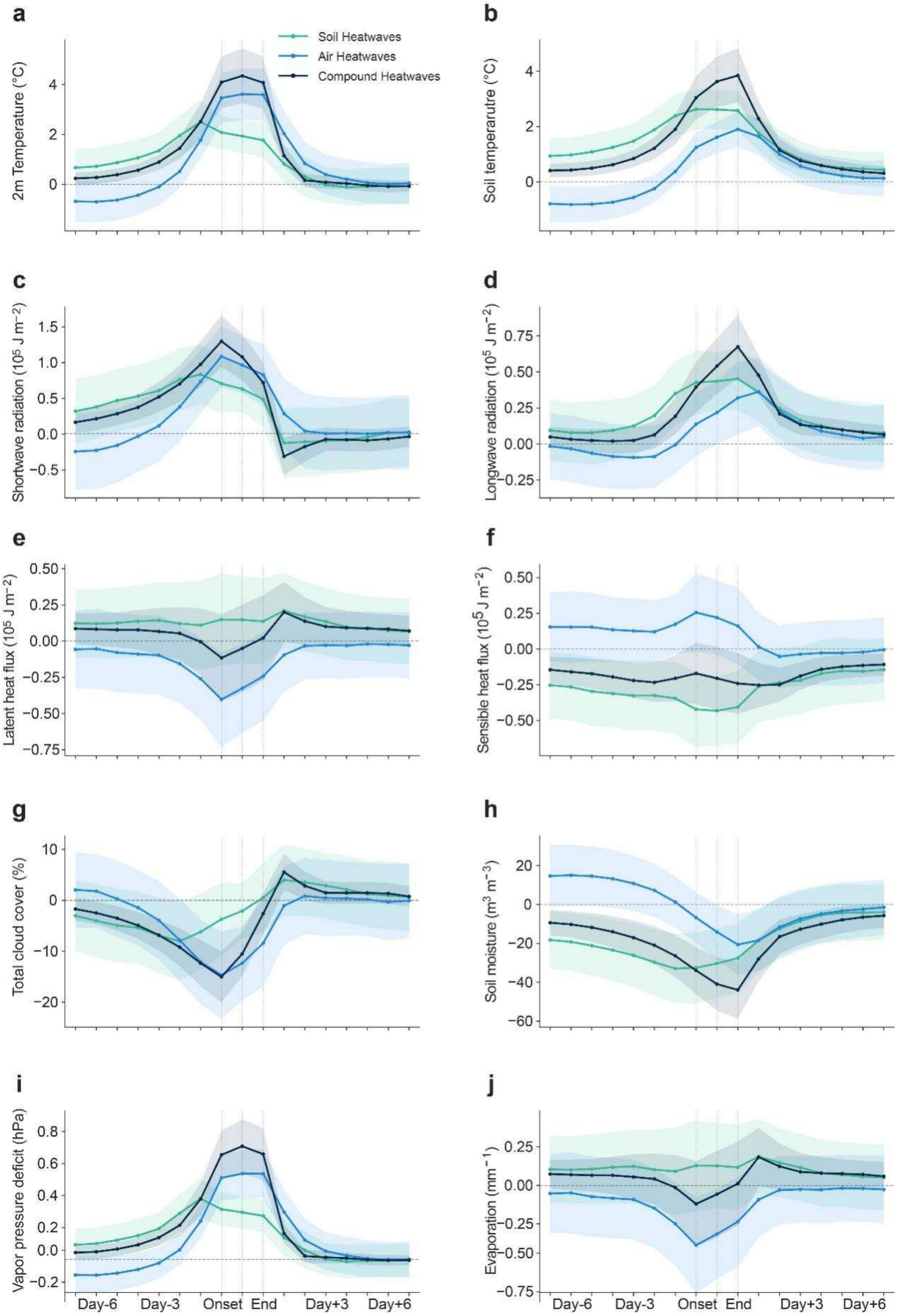

Supplementary Fig. 24 Temporal evolution of land-atmosphere variables associated with independent and compound air-soil heatwaves. Composite anomalies in near-surface air temperature **(a)**, soil temperature **(b)**, surface shortwave radiation **(c)**, surface downward longwave radiation **(d)**, latent heat flux **(e)**, sensible

heat flux **(f)**, total cloud cover **(g)**, soil moisture **(h)**, vapor pressure deficit **(i)**, and evaporation **(j)** from seven days before heatwave event onset to seven days after the termination. Evaporation is positive downwards. Blue, green, and grey lines represent independent air heatwaves, independent soil heatwaves, and compound air-soil heatwaves, respectively. Events followed by another heatwave within the preceding and subsequent seven days are excluded. Vertical dotted lines mark event onset, during, and termination. Solid line represents the time series of regional mean and the shaded regions represent half of the standard deviation.

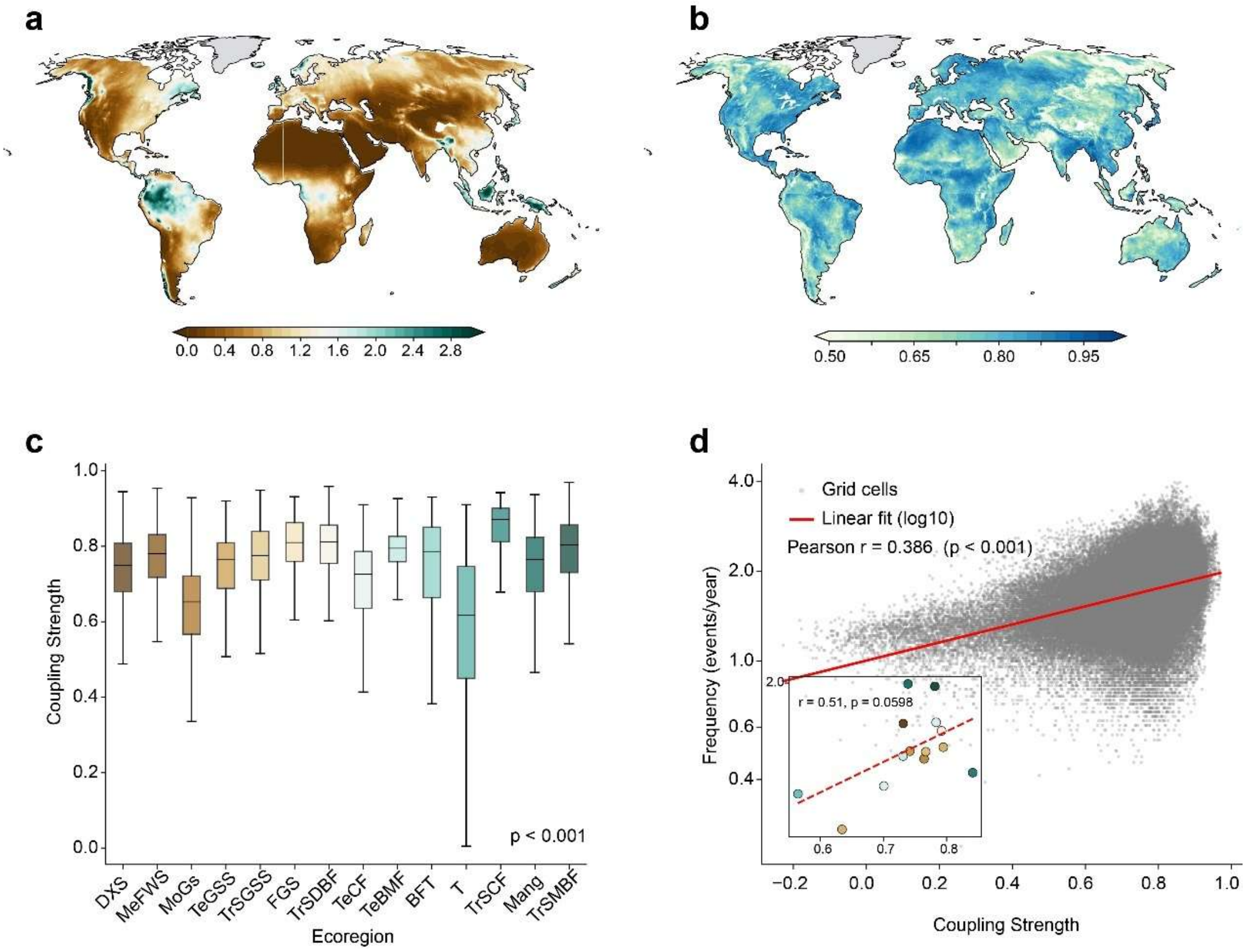

Supplementary Fig. 25 Air-soil temperature coupling and its relationship with compound air-soil heatwave frequency. a and **b,** Spatial distributions of aridity index (**a**) and air-soil temperature coupling strength (**b**). The aridity index is calculated as annual precipitation divided by potential evapotranspiration[28], with higher values indicating wetter conditions. Coupling strength is quantified as the Pearson correlation coefficient between near-surface air temperature and shallow soil temperature anomalies at each grid cell. **c,** Distributions of coupling strength across 14 terrestrial biomes (DXS, Deserts and Xeric Shrublands; MeFWS, Mediterranean Forests, Woodlands, and Scrub; MoGS, Montane Grasslands and Shrublands; TeGSS, Temperate Grasslands, Savannas, and Shrublands; TrSGSS, Tropical and Subtropical Grasslands, Savannas, and Shrublands; FGS, Flooded Grasslands and Savannas; TrSDBF, Tropical and Subtropical Dry Broadleaf Forests; TeCF, Temperate Conifer

Forests; TeBMF, Temperate Broadleaf and Mixed Forests; BFT, Boreal Forests or Taiga; T, Tundra; TrSCF, Tropical and Subtropical Coniferous Forests; Mang, Mangroves; and TrSMBF, Tropical and Subtropical Moist Broadleaf Forests)[29]. Boxplots show the median, interquartile range, and whiskers extending to 1.5 times the interquartile range; *P* values are from the Kruskal-Wallis test of comparing coupling strength among biomes. **d**, Relationships between the frequency of compound air-soil heatwaves and coupling strength at the grid scale. Grey points represent individual grid cells, red lines show fitted relationships, and the inset map shows biome mean values colored according to the aridity index.

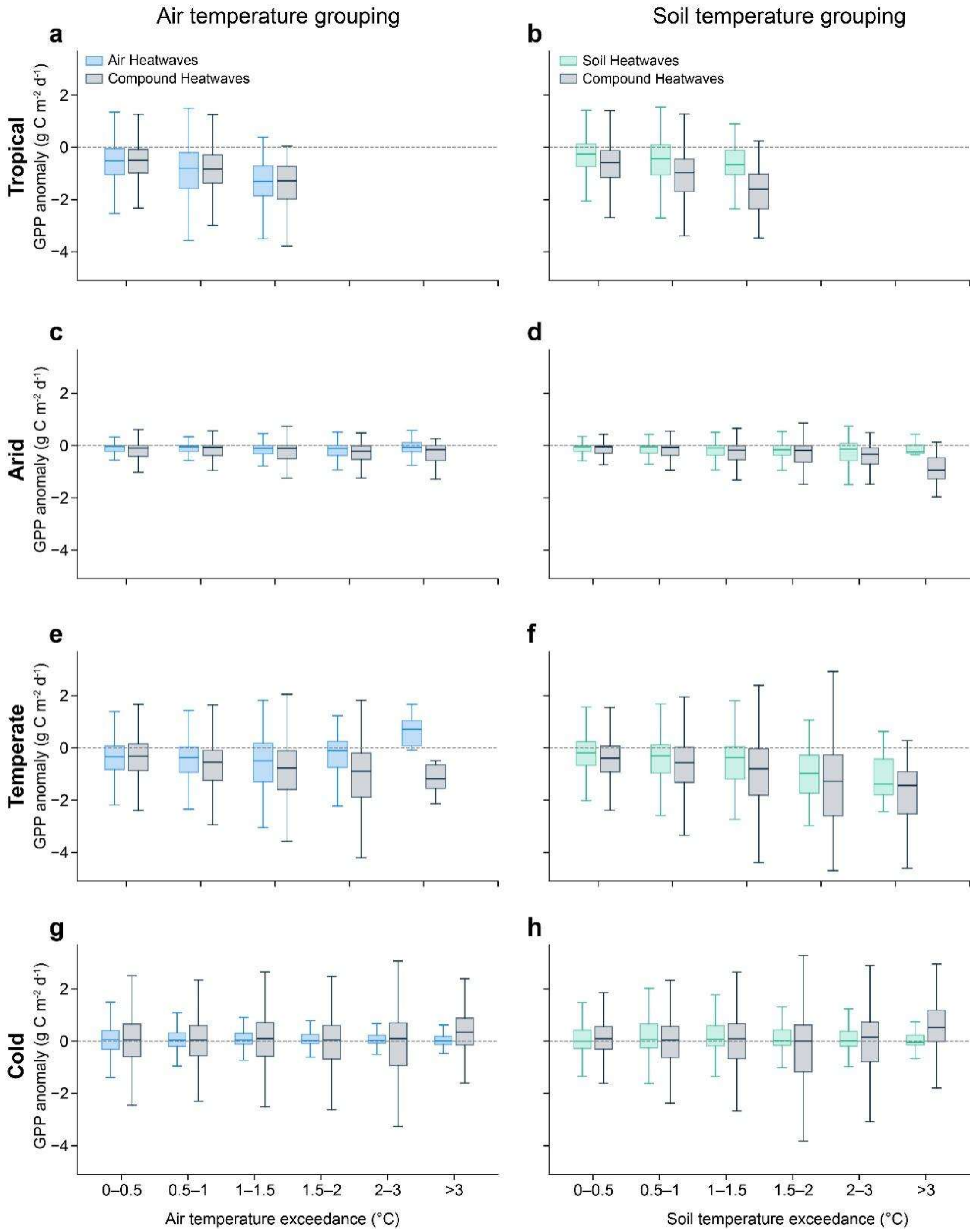

Supplementary Fig. 26 Comparison of GPP responses between independent and compound air-soil heatwaves after matching temperature exceedance and event duration. a–h, Box plots showing the distributions of GPP anomalies within bins of mean temperature exceedance across tropical (**a, b**), arid (**c, d**), temperate (**e, f**), and cold (**g, h**) zones. The GPP anomaly is calculated as the minimum daily GPP anomaly recorded during each heatwave event. **a**, Comparison between independent air heatwaves (blue) and compound air-soil heatwaves (grey) across bins of mean air temperature exceedance. **b**, Comparison between independent soil heatwaves (green) and compound air-soil heatwaves (grey) across bins of mean soil temperature

exceedance. The centerline indicates the median value, the box bounds depict the 25th and 75th percentile values and the whiskers indicate the lower and upper quartiles±1.5 times the interquartile range of the values.

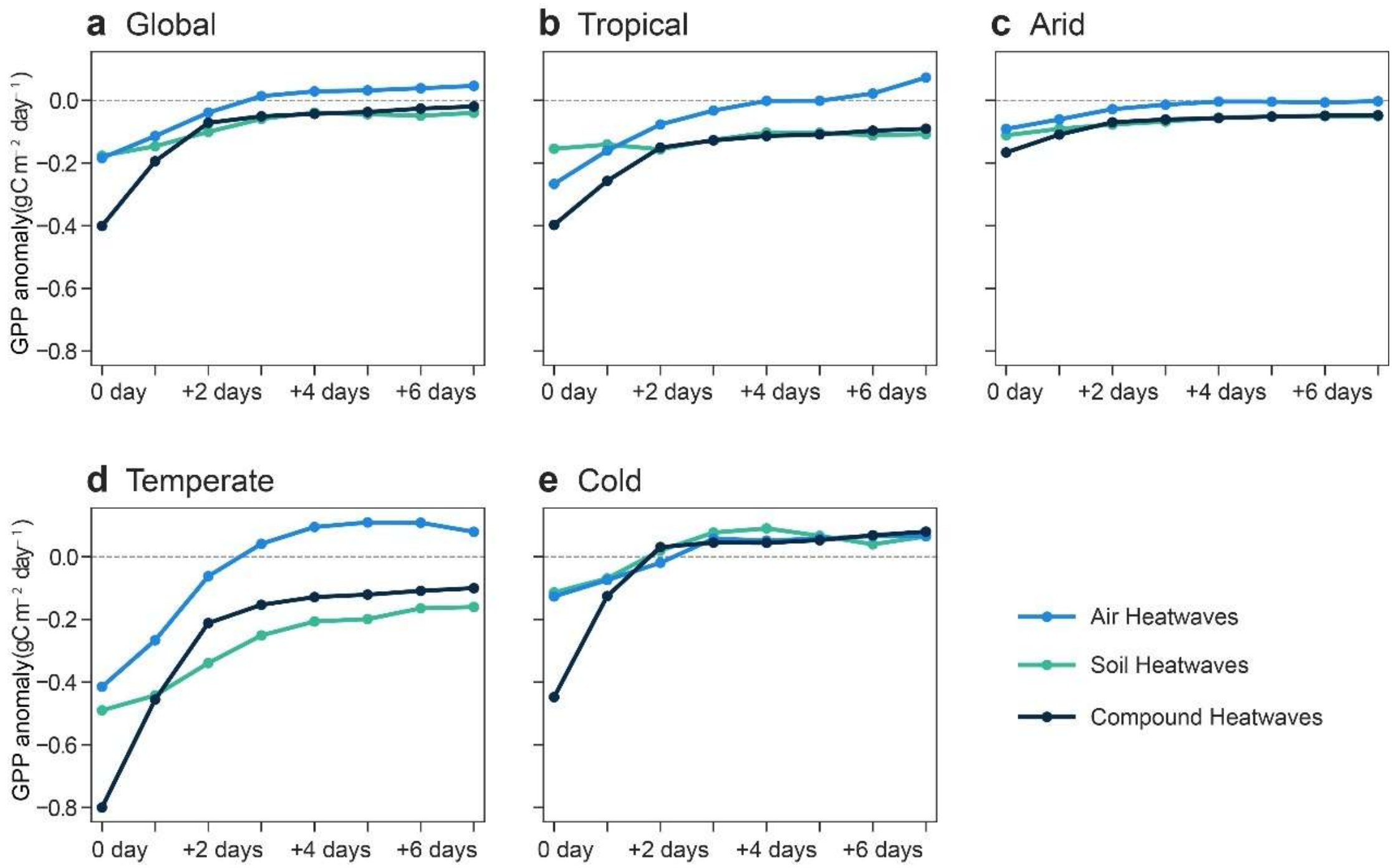

Supplementary Fig. 27 Temporal evolution of GPP anomalies during the following seven days after the termination of independent and compound air-soil heatwaves. Regional mean of GPP anomalies from the end day of each heatwave event (i.e., day 0) to seven days after event termination (i.e., day +7) over global land areas **(a)** and in tropical **(b)**, arid **(c)**, temperate **(d)**, and cold **(e)** climate zones. Blue, green, and grey lines represent independent air heatwaves, independent soil heatwaves, and compound air-soil heatwaves, respectively. Events followed by another heatwave within the subsequent seven days are excluded.

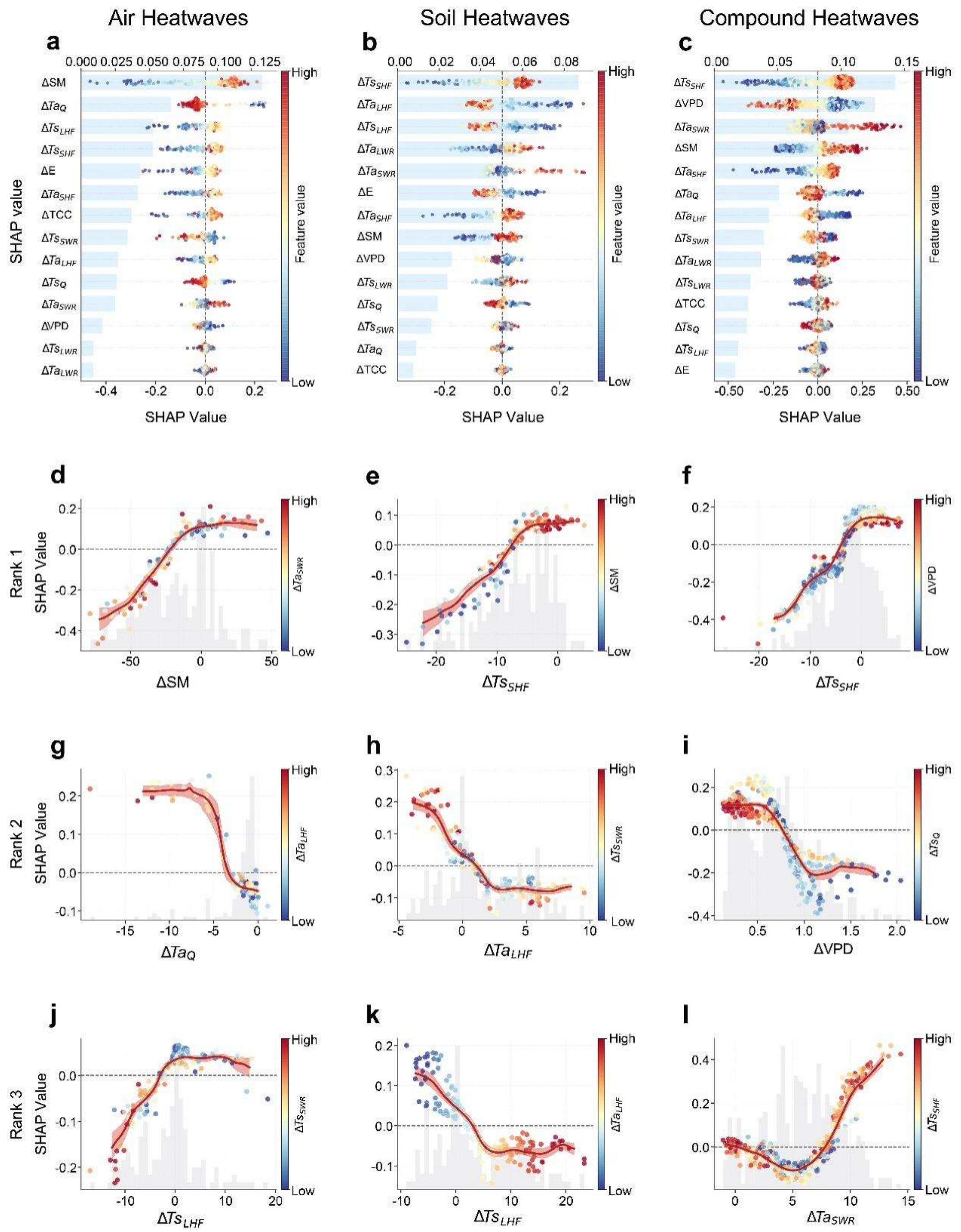

Supplementary Fig. 28 SHAP-based interpretation of random forest models for GPP anomalies during independent and compound air-soil heatwaves. a–c, SHAP beeswarm plots for random forest models predicting GPP anomalies during independent air heatwaves, independent soil heatwaves, and compound air-soil heatwaves over global land areas. Predictors are ranked by mean absolute SHAP values, with light blue bars showing mean |SHAP|. Each point represents one testing grid cell, and colors indicate the corresponding predictor value. Positive SHAP values indicate contributions toward higher predicted GPP anomalies, whereas negative values indicate

contributions toward stronger predicted GPP reductions. The GPP product is derived from BESS v2.0 (the Breathing Earth System Simulator) dataset (1982–2022) [30]. **d–l,** SHAP dependence plots for the three highest-ranked predictors in each heatwave model. Grey histograms show the distribution of the focal predictor. Red curves show locally weighted mean SHAP responses, and shaded bands indicate the 95% confidence interval. Point colors indicate the predictor with the strongest approximate SHAP interaction with the focal predictor, or the most strongly correlated predictor when interaction selection is unavailable. Predictors are grouped into: 1) air temperature contributions (ΔTa), including contributions from net surface shortwave radiation (ΔTa_{SWR}), downward longwave radiation (ΔTa_{LWR}), latent heat flux (ΔTa_{LHF}), sensible heat flux (ΔTa_{SHF}), and ground heat flux (ΔTa_{Q}); 2) soil temperature contributions (ΔTs), including net surface shortwave radiation (ΔTs_{SWR}), downward longwave radiation (ΔTs_{LWR}), latent heat flux (ΔTs_{LHF}), sensible heat flux (ΔTs_{SHF}), and ground heat flux (ΔTs_{Q}), and 3) other climate factors, including total cloud cover (ΔTCC), soil moisture (ΔSM), vapor pressure deficit (ΔVPD), and evaporation (ΔE).

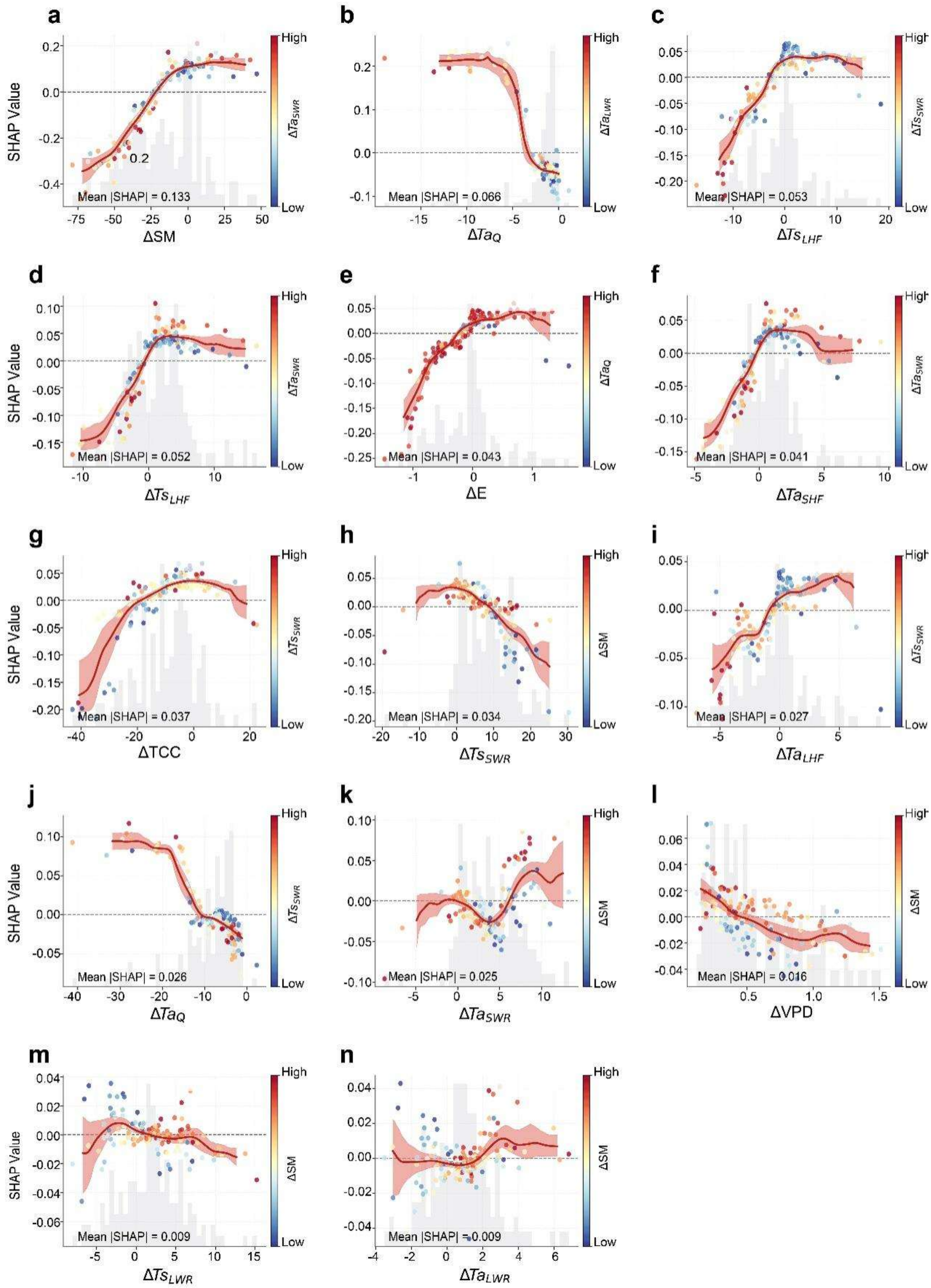

Supplementary Fig. 29 SHAP dependence plots for the ranking predictors in independent air heatwave model. Grey histograms show the distribution of the focal predictor. Red curves show locally weighted mean SHAP responses, and shaded bands indicate 95% confidence interval. Point colors indicate the predictor with the strongest approximate SHAP interaction with the focal predictor, or the most strongly correlated

predictor when interaction selection is unavailable.

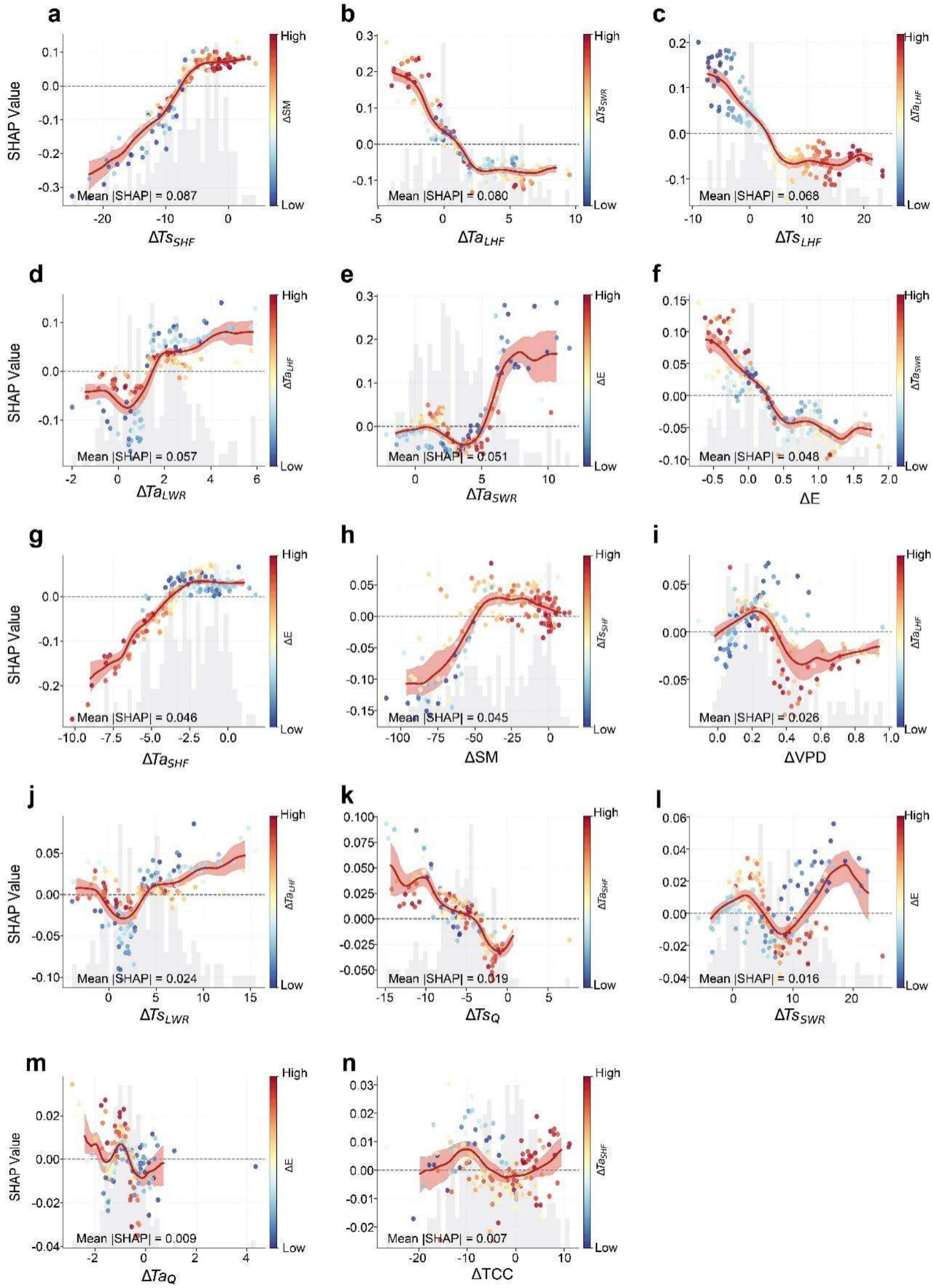

Supplementary Fig. 30 SHAP dependence plots for the ranking predictors in independent soil heatwave model. Grey histograms show the distribution of the focal predictor. Red curves show locally weighted mean SHAP responses, and shaded bands

indicate 95% confidence interval. Point colors indicate the predictor with the strongest approximate SHAP interaction with the focal predictor, or the most strongly correlated predictor when interaction selection is unavailable.

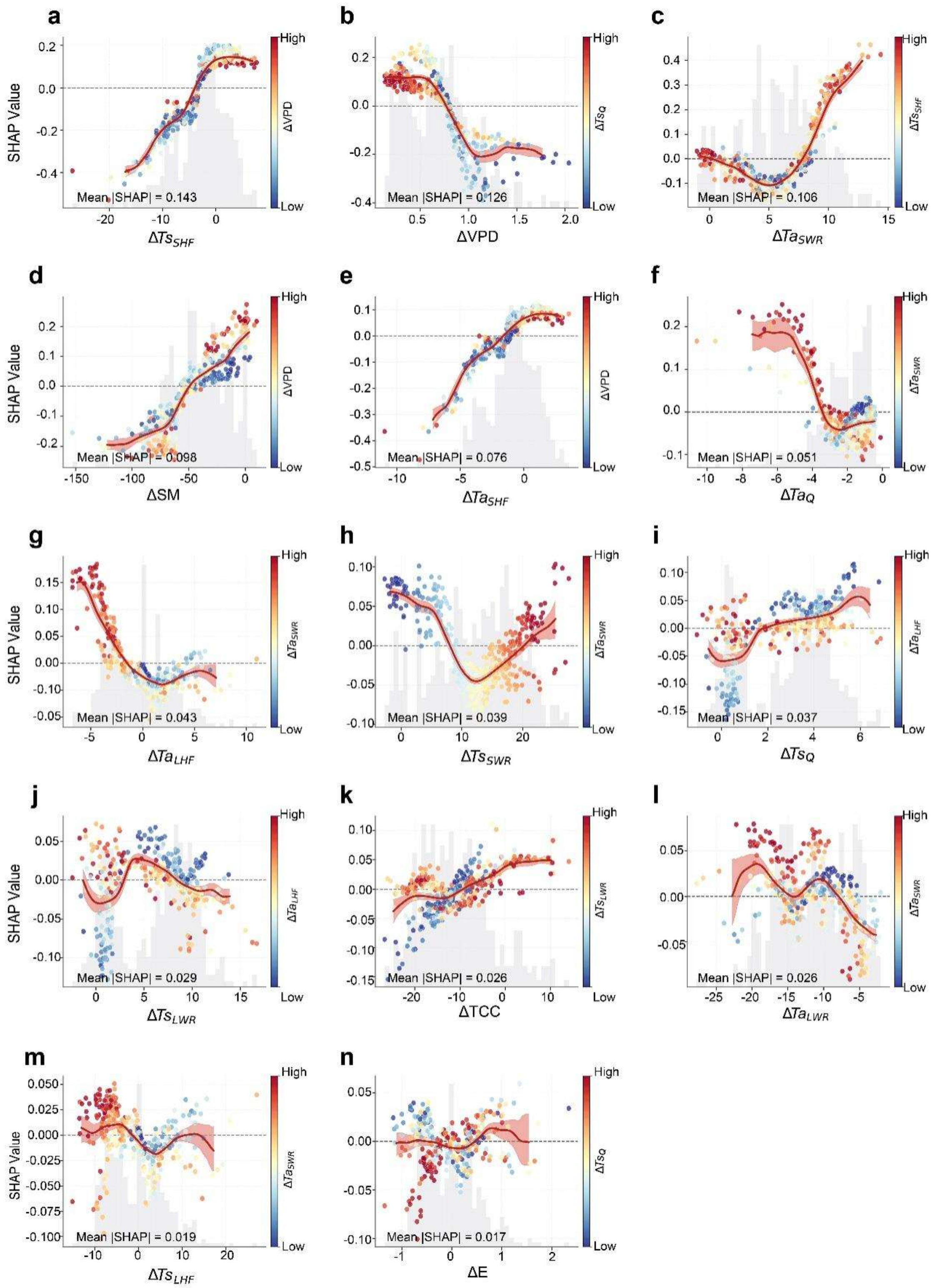

Supplementary Fig. 31 SHAP dependence plots for the ranking predictors in

compound air-soil heatwave model. Grey histograms show the distribution of the focal predictor. Red curves show locally weighted mean SHAP responses, and shaded bands indicate 95% confidence interval. Point colors indicate the predictor with the strongest approximate SHAP interaction with the focal predictor, or the most strongly correlated predictor when interaction selection is unavailable.

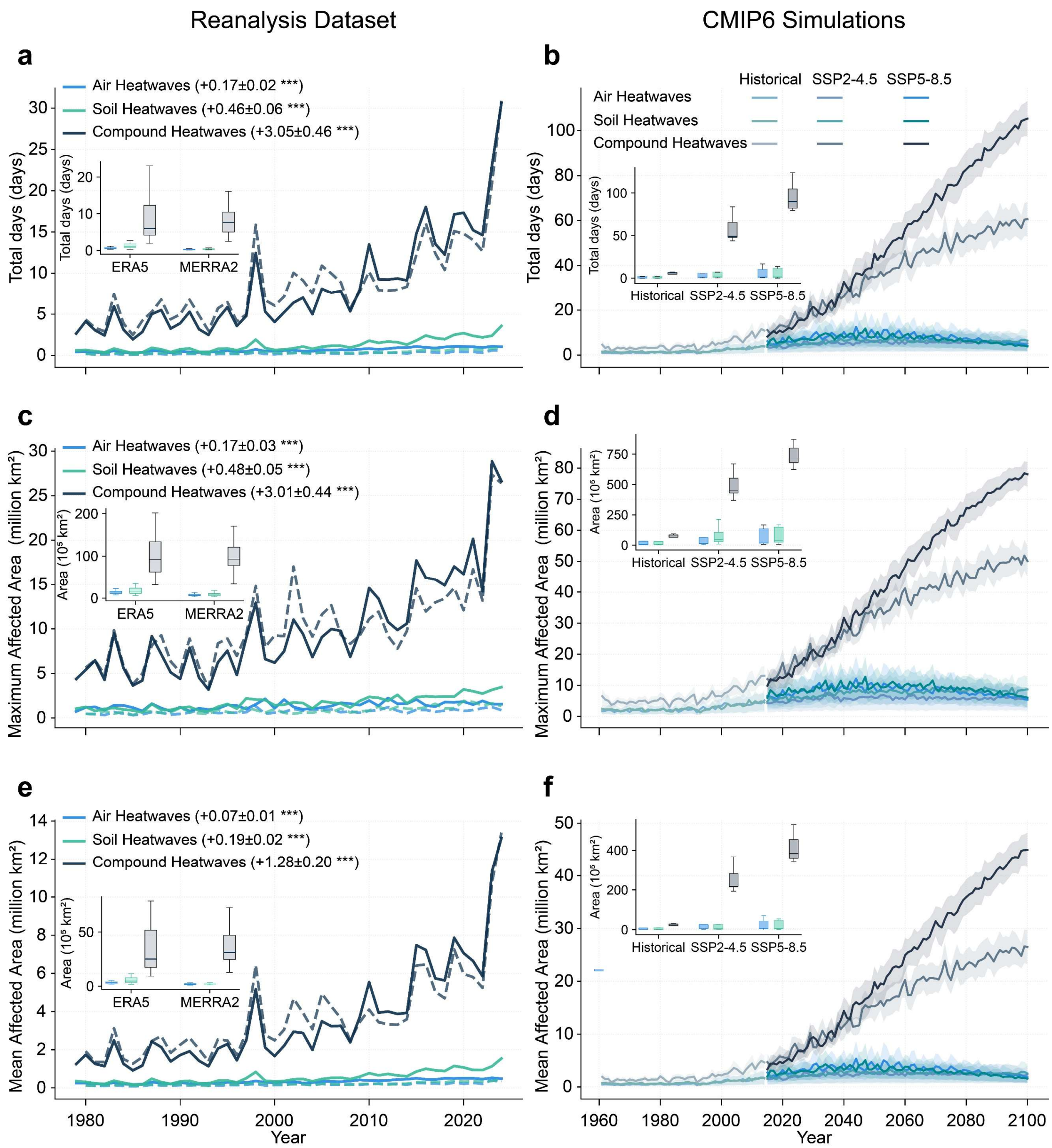

Supplementary Fig. 32 Historical and projected changes in the total days and spatial extent of independent and compound air-soil heatwaves. a–f, Time series of annual total days (**a, b**), maximum affected area (**c, d**), and mean affected area (**e, f**) for independent air heatwaves (blue), independent soil heatwaves (green), and compound air-soil heatwaves (grey). Left panel (**a, c, e**) represents that the estimates from reanalysis, with ERA5 (1979–2024; solid lines) and MERRA-2 (1980–2024; dashed lines). The numbers in parentheses indicate the decadal trends (per decade) with the

standard error. *$p < 0.05$ for long-term trend; **$p < 0.01$; ***$p < 0.001$. The embedded box plots show the distribution of the annual metrics for the two datasets. Right panel (**b, d, f**) represents that the estimates of historical (1961–2014) and projected (2015–2100) changes from CMIP6 under SSP2–4.5 and SSP5–8.5. Solid lines show the multi-model ensemble mean and shading indicates ± half the inter-model standard deviation. The embedded box plots display the distribution of the metrics during the baseline period (1981–2010) and the late 21st century (2081–2100) under the two future scenarios.

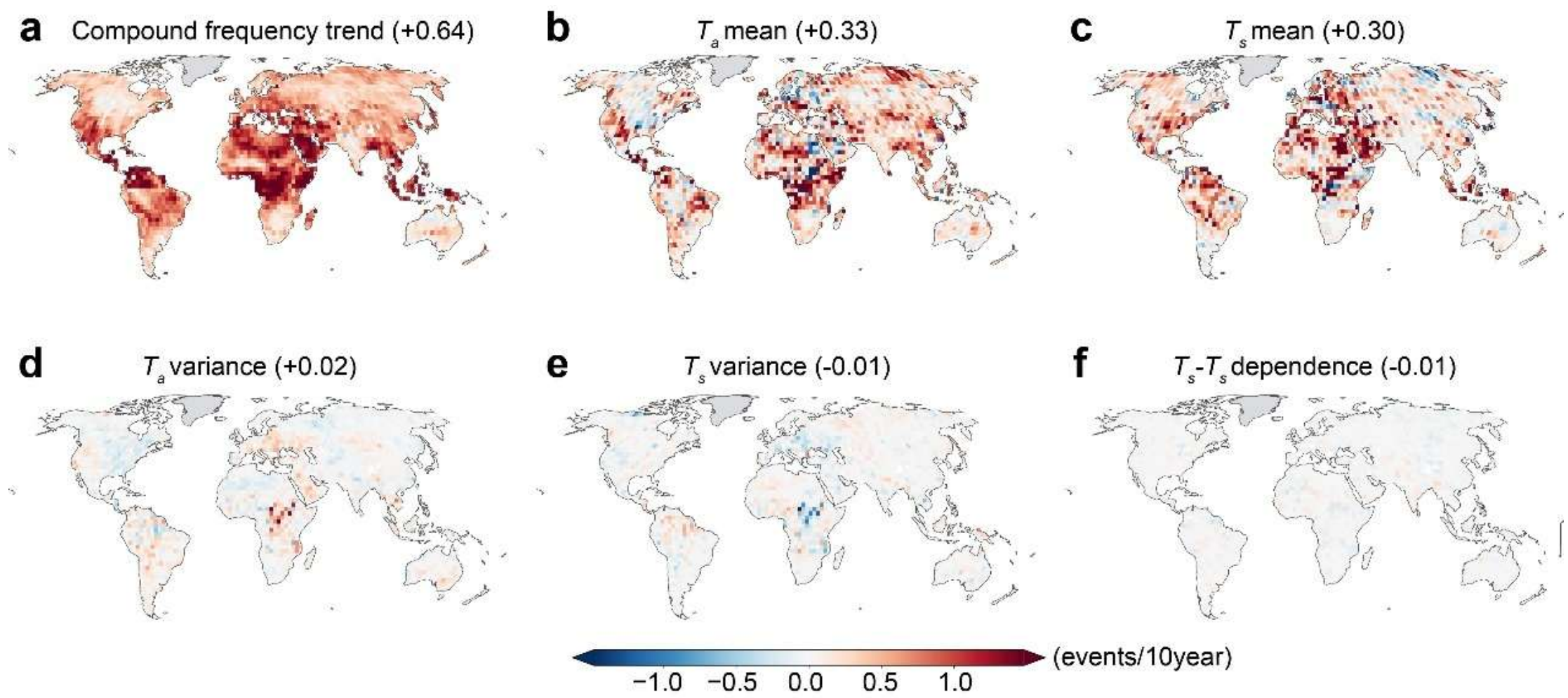

Supplementary Fig. 33 Decomposition of the historical trend in compound air-soil heatwave frequency. a, Spatial distribution of the reconstructed trend in annual compound air-soil heatwave frequency, calculated as the sum of the five fitted component trends. **b–f,** Contributions associated with changes in mean near-surface air temperature (**b**), mean soil temperature (**c**), air temperature variance (**d**), soil temperature variance (**e**), and air-soil temperature dependence (**f**). Air-soil temperature dependence is represented by the annual warm-season Pearson correlation between daily air and soil temperatures. Separate linear models are fitted at each grid cell, and trends in the fitted components are estimated using the Theil-Sen estimator. Values in parentheses indicate the area-weighted global means of the trends.

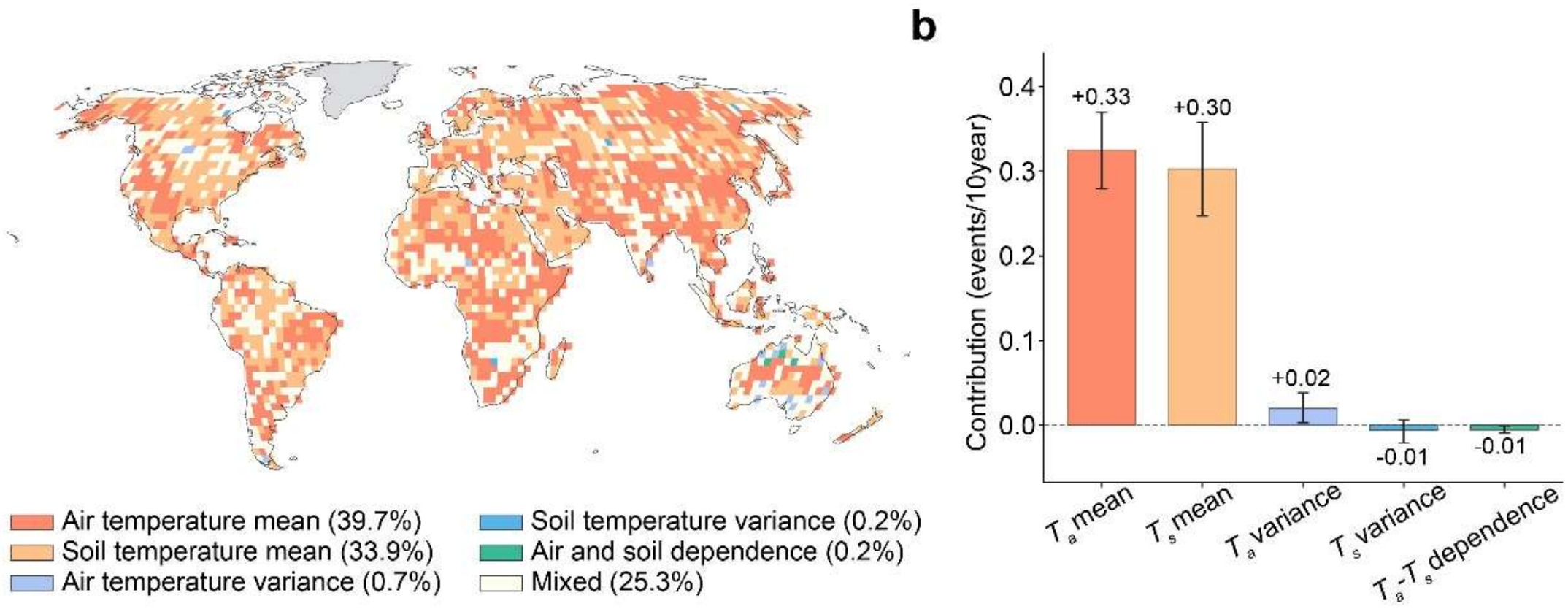

Supplementary Fig. 34 Contributions to the historical trend in compound air-soil heatwave frequency. a, Spatial distribution of the dominant contribution at each grid cell. Dominance is determined from the largest absolute contribution among mean air temperature, mean soil temperature, air temperature variance, soil temperature variance, and air-soil temperature dependence. Grid cells are classified as mixed when the largest component accounts for less than 50% of the sum of the five absolute contributions. Percentages in the legend indicate the area-weighted fractions of evaluated global land.

b, Area-weighted global mean contribution of each fitted component. Error bars indicate the 95% confidence intervals estimated using a spatial block bootstrap.

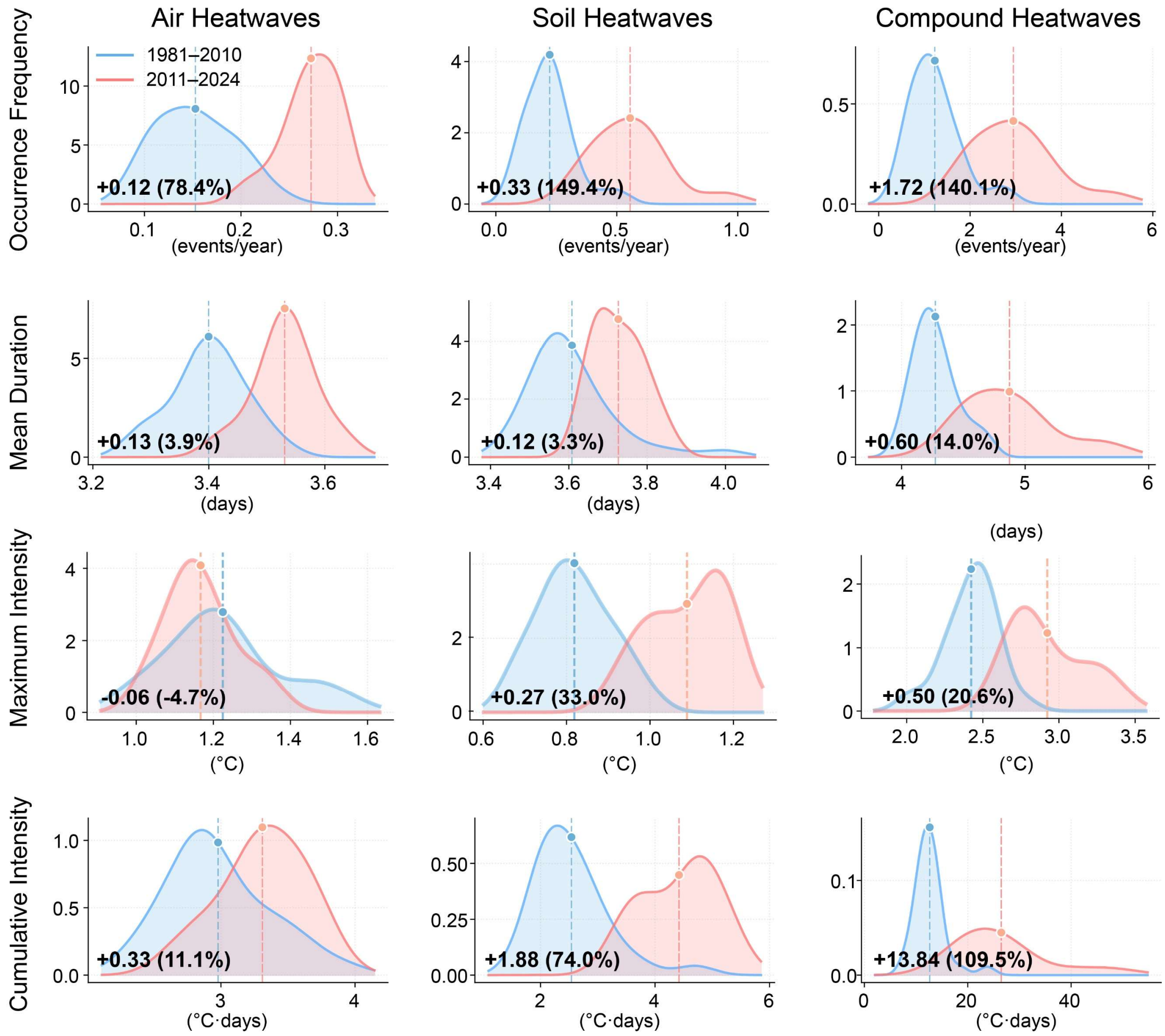

Supplementary Fig. 35 Shifts in the probability distributions of heatwave characteristics between 1981–2010 and 2011–2024. The probability density functions (PDFs) of spatially averaged annual metrics for independent air heatwaves (left column), independent soil heatwaves (middle column), and compound air-soil heatwaves (right column). The rows represent occurrence frequency, mean duration, maximum intensity, and cumulative intensity, respectively. Blue curves and shading indicate the baseline period (1981–2010), while red curves and shading indicate the recent period (2011–2024). Vertical dashed lines and dots mark the mean values of the distributions for each period. The text annotations provide the absolute change in the mean value (recent period minus baseline period), and the corresponding percentage change is given in parentheses.

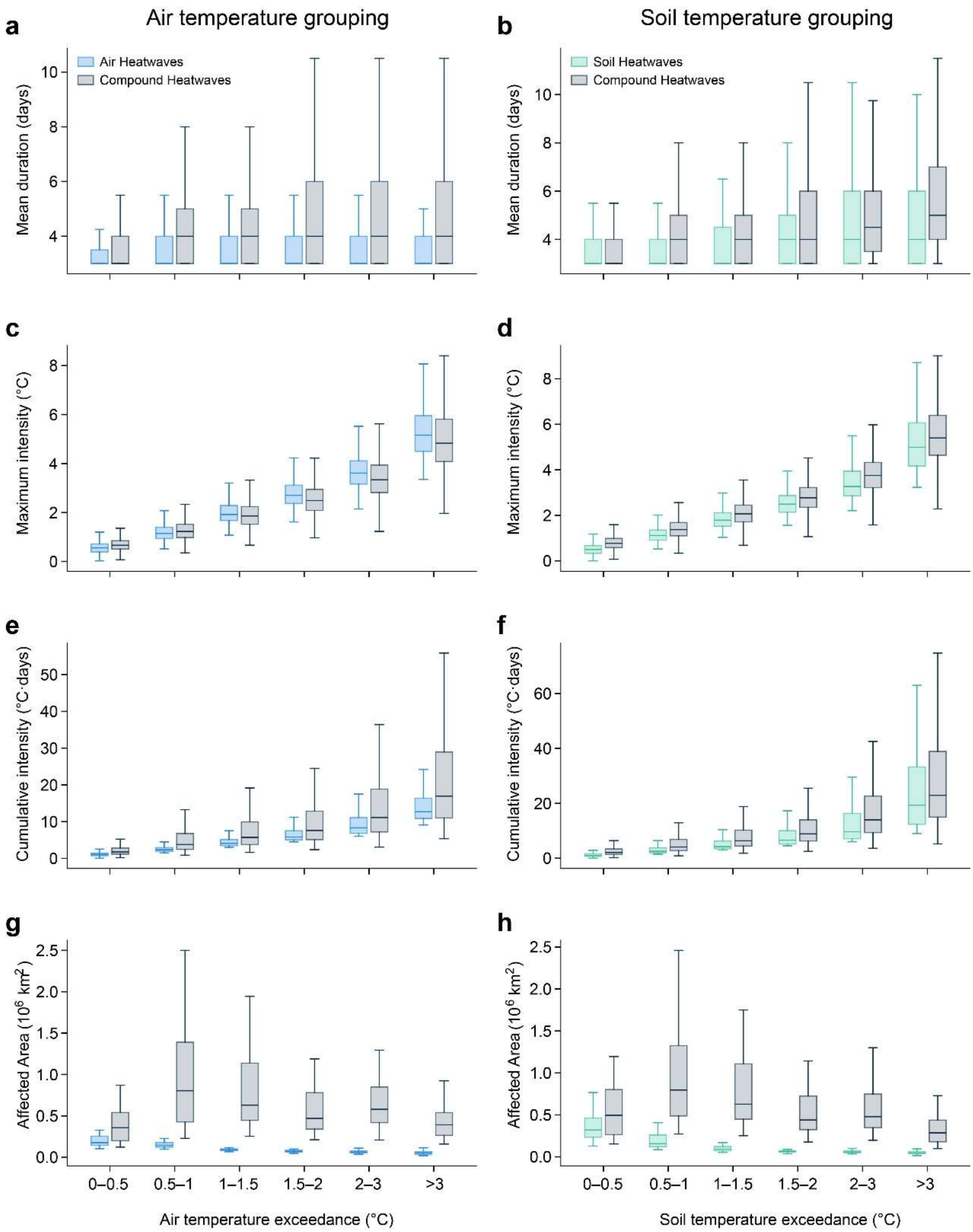

Supplementary Fig. 36 Comparison of independent and compound air-soil heatwaves under comparable temperature exceedance. a–h, Box plots showing the distributions of mean duration **(a, b)**, maximum intensity **(c, d)**, cumulative intensity **(e, f)**, and mean affected area **(g, h)** within bins of mean temperature exceedance during each event. **a, c, e, g,** Comparison of independent air heatwaves (blue) with compound air-soil heatwaves (grey) using bins of mean air temperature exceedance. **b, d, f, h**, Comparison of independent soil heatwaves (green) with compound air-soil heatwaves (grey) using bins of mean soil temperature exceedance. The centerline indicates the median value, the box bounds depict the 25th and 75th percentile values and the whiskers

indicate the lower and upper quartiles±1.5 times the interquartile range of the values.

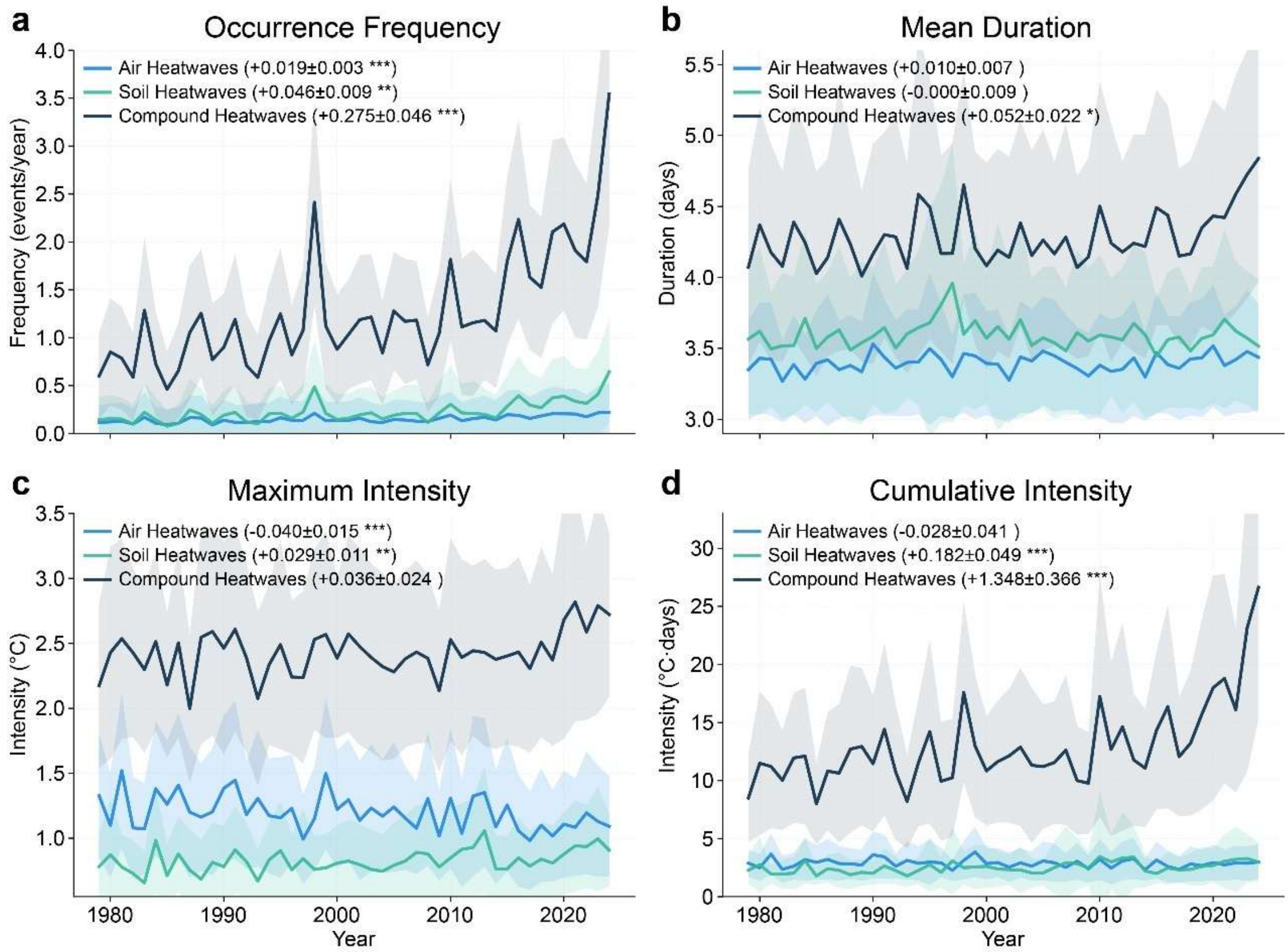

Supplementary Fig. 37 Long-term changes in independent and compound air-soil heatwaves based on a sliding 30-year baseline. a–f, Time series of the spatially averaged annual mean of occurrence frequency (**a**), mean duration (**b**), maximum intensity (**c**), and cumulative intensity (**d**) of independent air heatwaves (blue), independent soil heatwaves (green), and compound air-soil heatwaves (grey) over global land areas during cold season from 1979 to 2024. Solid line represents the time series of regional mean and the shaded regions represent half of the standard deviation. Slope estimates for the trend per decade are given in parentheses. *$p < 0.05$ for long-term trend; **$p < 0.01$; ***$p < 0.001$. The cold season is defined as November–March in the Northern Hemisphere and May–September in the Southern Hemisphere.

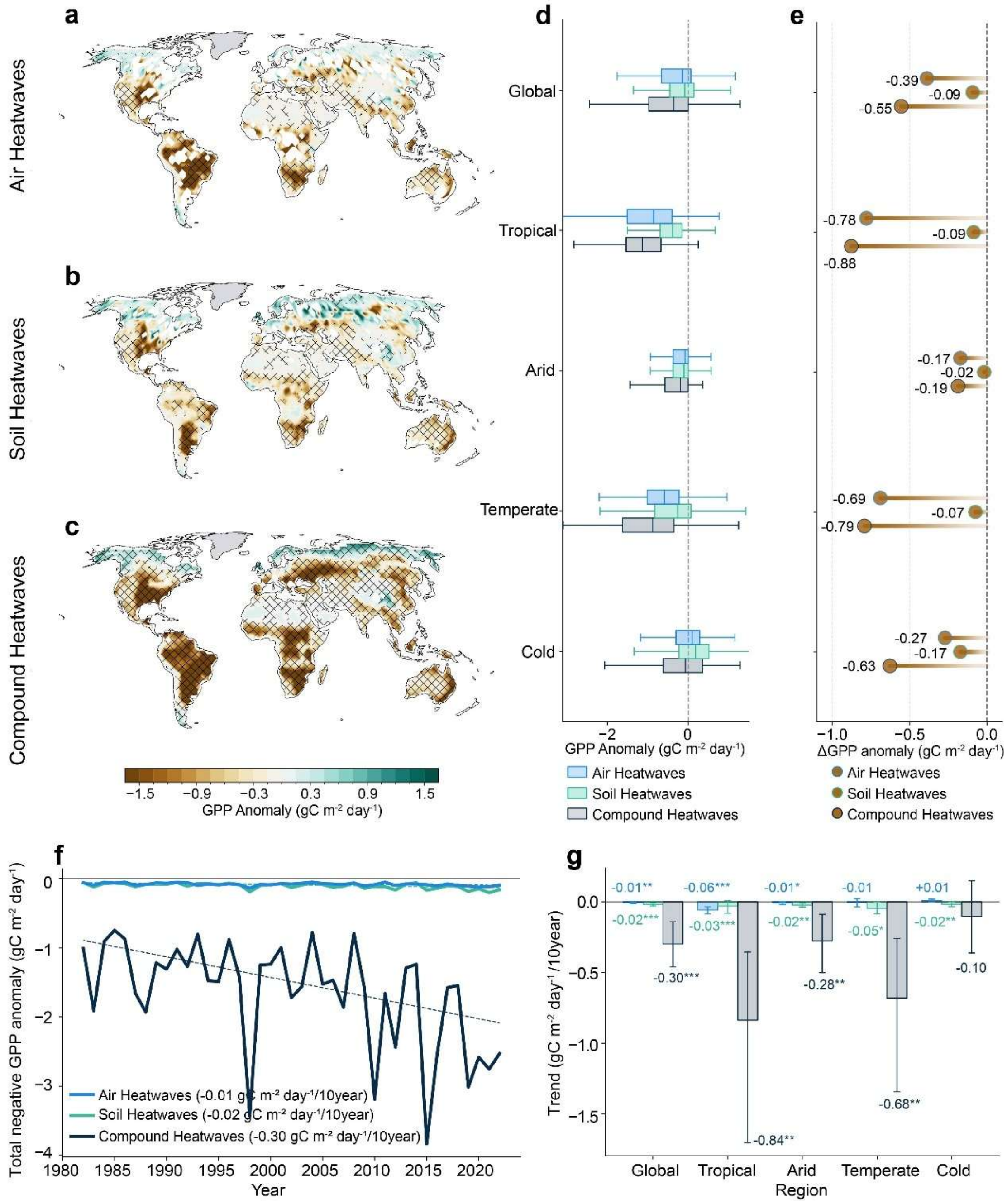

Supplementary Fig. 38 Ecological impacts of independent and compound air-soil heatwaves based on a sliding 30-year baseline. a–c, Spatial distribution of the composite gross primary productivity (GPP) anomalies during independent air heatwaves (**a**), independent soil heatwaves (**b**), and compound air-soil heatwaves (**c**) over global land areas. The GPP product is derived from BESS v2.0 (the Breathing Earth System Simulator) dataset (1982–2022) [30]. Regions where the composite mean passes the 0.1 significance level by the Student's *t*-test are marked by cross-hatching. **d**, Boxplots showing the distribution of regional composite GPP anomalies for during independent air heatwaves (blue), independent soil heatwaves (green), and compound air-soil heatwaves (grey) over global land areas and across different climate zones. **e**, Difference between the minimum composite anomalies of GPP during the heatwave

event and the GPP anomaly on 1 day before the onset over global land areas and across different climate zones. Negative values (brown) indicate a reduction in GPP triggered by the heatwave relative to the pre-event state. **f–g,** Changes in ecological impacts of independent and compound air-soil heatwaves. **f**, Time series of the spatially averaged annual GPP loss, defined as the sum of negative GPP anomalies on heatwave days from 1982 to 2022. Dashed lines indicate the corresponding linear trends that pass the 0.05 significance level based on the Theil-Sen estimator. Slope estimates for the trend per decade are given in parentheses. **g**, Decadal trends of the annual GPP loss for three types of heatwaves over global land areas and across different climate zones. The error bars indicate the 95% bootstrap confidence interval of the trends. *$p < 0.05$ for long-term trend; **$p < 0.01$; ***$p < 0.001$.

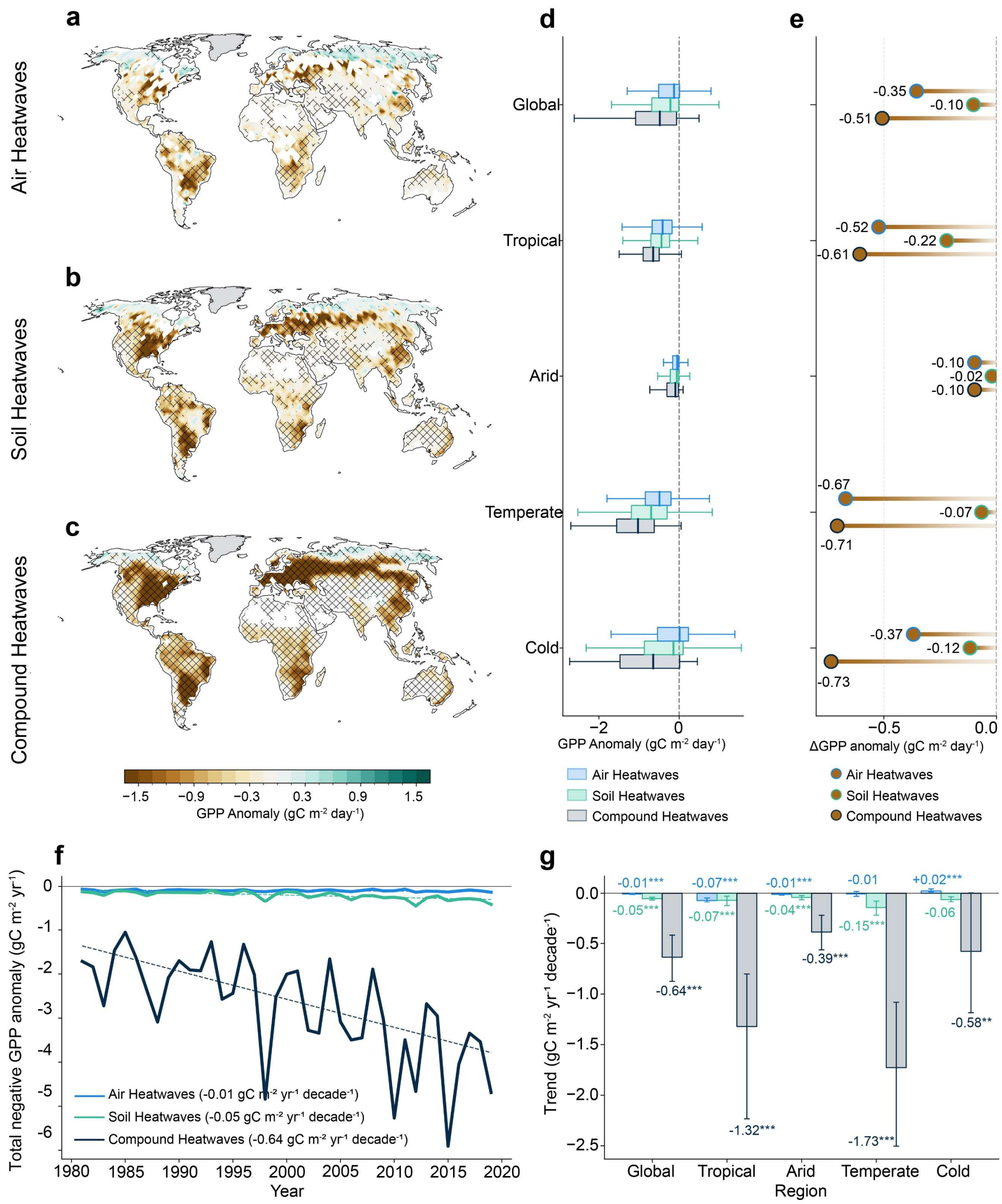

Supplementary Fig. 39 Ecological impacts of independent and compound air-soil heatwaves. a–c, Spatial distribution of the composite gross primary productivity (GPP) anomalies during independent air heatwaves (**a**), independent soil heatwaves (**b**), and compound air-soil heatwaves (**c**) over global land areas. The GPP product is generated by the Boreal Ecosystem Productivity Simulator (BEPS) model for 1981–2019 [14, 31]. Regions where the composite mean passes the 0.1 significance level by the Student's *t*-test are marked by cross-hatching. **d**, Boxplots showing the distribution of regional composite GPP anomalies during independent air heatwaves (blue), independent soil heatwaves (green), and compound air-soil heatwaves (grey) over global land areas and across different climate zones. **e**, Difference between the minimum composite

anomalies of GPP during the heatwave event and the GPP anomaly on 1 day before the onset over global land areas and across different climate zones. Negative values (brown) indicate a reduction in GPP triggered by the heatwave relative to the pre-event state. **f–g,** Changes in ecological impacts of independent and compound air-soil heatwaves. **f**, Time series of the spatially averaged annual GPP loss, defined as the sum of negative GPP anomalies on heatwave days from 1981 to 2019. Dashed lines indicate the corresponding linear trends that pass the 0.05 significance level based on the Theil-Sen estimator. Slope estimates for the trend per decade are given in parentheses. **g**, Decadal trends of the annual GPP loss for three types of heatwaves over global land areas and across different climate zones. The error bars indicate the 95% bootstrap confidence interval of the trends. *$p < 0.05$ for long-term trend; **$p < 0.01$; ***$p < 0.001$.

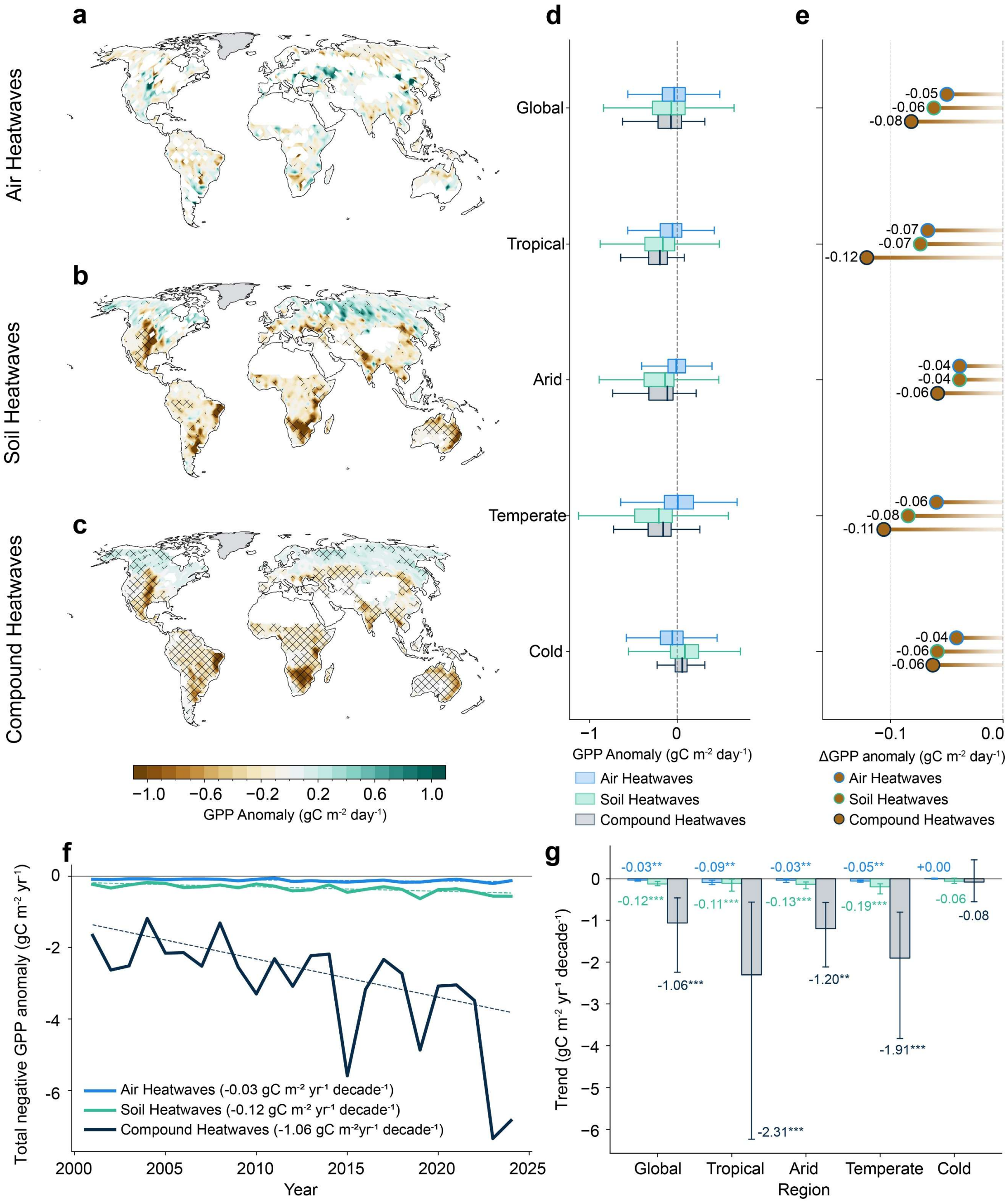

Supplementary Fig. 40 Ecological impacts of independent and compound air-soil heatwaves. a–c, Spatial distribution of the composite gross primary productivity (GPP) anomalies during independent air heatwaves (**a**), independent soil heatwaves (**b**), and compound air-soil heatwaves (**c**) over global land areas. The GPP product is FluxSat GPP version 2.2 product during 2001–2024 [32]. Regions where the composite mean passes the 0.1 significance level by the Student's t-test are marked by cross-hatching. **d**, Boxplots showing the distribution of regional composite GPP anomalies during independent air heatwaves (blue), independent soil heatwaves (green), and compound air-soil heatwaves (grey) over global land areas and across different climate zones. **e**, Difference between the minimum composite anomalies of GPP during the heatwave event and the GPP anomaly on 1 day before the onset over global land areas and across

different climate zones. Negative values (brown) indicate a reduction in GPP triggered by the heatwave relative to the pre-event state. **f–g,** Changes in ecological impacts of independent and compound air-soil heatwaves. **f**, Time series of the spatially averaged annual GPP loss, defined as the sum of negative GPP anomalies on heatwave days from 2001 to 2024. Dashed lines indicate the corresponding linear trends that pass the 0.05 significance level based on the Theil-Sen estimator. Slope estimates for the trend per decade are given in parentheses. **g**, Decadal trends of the annual GPP loss for three types of heatwaves over global land areas and across different climate zones. The error bars indicate the 95% bootstrap confidence interval of the trends. *$p < 0.05$ for long-term trend; **$p < 0.01$; ***$p < 0.001$.

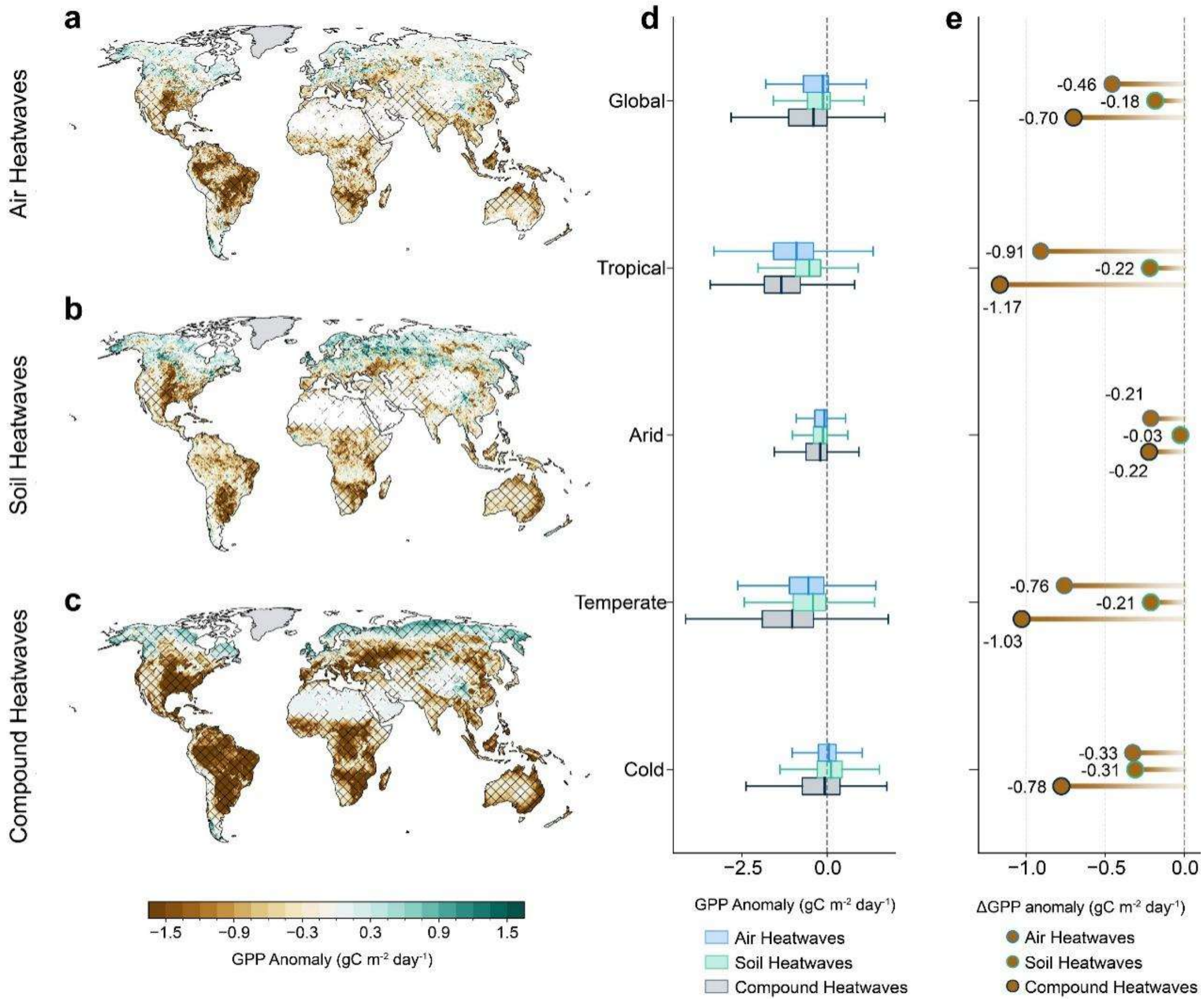

Supplementary Fig. 41 Ecological impacts of independent and compound air-soil heatwaves at the 0.25°×0.25° resolution. a–c, Spatial distribution of the composite gross primary productivity (GPP) anomalies during independent air heatwaves (**a**), independent soil heatwaves (**b**), and compound air-soil heatwaves (**c**) over global land areas. The GPP product is derived from the BESS v2.0 (the Breathing Earth System Simulator) dataset (1982–2022) [30], with both heatwave metrics and GPP evaluated at 0.25° × 0.25° resolution. Regions where the composite mean passes the 0.1

significance level by the Student's *t*-test are marked by cross-hatching. **d**, Boxplots showing the distribution of regional composite GPP anomalies for during independent air heatwaves (blue), independent soil heatwaves (green), and compound air-soil heatwaves (grey) over global land areas and across different climate zones. **e**, Difference between the minimum composite anomalies of GPP during the heatwave event and the GPP anomaly on 1 day before the onset over global land areas and across different climate zones. Negative values (brown) indicate a reduction in GPP triggered by the heatwave relative to the pre-event state.

Supplementary Table 1. Predictive performance of the random forest models during independent and compound air-soil heatwaves.

Event	Train R^2	Test R^2	Test RMSE	Test EV	out-of-bag R^2
Air heatwaves	0.943	0.522	0.430	0.525	0.589
Soil heatwaves	0.944	0.575	0.382	0.603	0.585
Compound heatwaves	0.962	0.709	0.414	0.709	0.722

Supplementary Table 2. Earth system models from CMIP6 used in this study. Models are listed in alphabetical order.

Model	Research institution	Native soil depth coordinates or layer midpoints (meter)
ACCESS-ESM1-5	Commonwealth Scientific and Industrial Research Organization, Australia	0.05
CESM2	National Center for Atmospheric Research, USA	0.01, 0.04, 0.09, 0.16, 0.26, 0.4, 0.58, 0.8, 1.06, 1.36, 1.7, 2.08, 2.5, 2.99, 3.58, 4.27, 5.06, 5.95, 6.94, 8.03, 9.80, 13.33, 19.48, 28.87, 42.00
EC-Earth3	Swedish Meteorological and Hydrological Institute, Sweden	0.05
IPSL-CM6A-LR	Institute Pierre- Simon Laplace, France	4.89×10^{-4}, 1.96×10^{-3}, 5.87×10^{-3}, 1.37×10^{-3}, 2.93×10^{-2}, 6.06×10^{-2}, 0.12, 0.25, 0.50, 1.00, 1.75, 2.5, 3.50, 5.50, 9.51, 17.52, 33.53, 65.56
MIROC6	Consortium AMSTEC, AORI, NIES, R-CCS, Japan	0.05
MIROC-ES2L	Consortium AMSTEC, AORI, NIES, R-CCS, Japan	0.05
MPI-ESM1-2-HR	Max Planck Institute for Meteorology, Germany	0.05
MPI-ESM1-2-LR	Max Planck Institute for Meteorology, Germany	0.03, 0.19, 0.78, 2.68, 6.98

Reference:

1. Perkins-Kirkpatrick SE, Lewis SC. Increasing trends in regional heatwaves. *Nat Commun*. 2020; **11**(1): 3357.
2. Lin L, Wu S, Shi M *et al.* Accelerating increases in soil heatwaves and cropland exposure in China. *Environ Res Lett*. 2026; **21**(1): 014024.
3. Beck HE, McVicar TR, Vergopolan N *et al.* High-resolution (1 km) Köppen-Geiger maps for 1901–2099 based on constrained CMIP6 projections. *Sci Data*. 2023; **10**(1): 724.
4. Röthlisberger M, Papritz L. Quantifying the physical processes leading to atmospheric hot extremes at a global scale. *Nat Geosci*. 2023; **16**(3): 210-216.
5. Tian Y, Kleidon A, Lesk C *et al.* Characterizing heatwaves based on land surface energy budget. *Commun Earth Environ*. 2024; **5**(1): 617.
6. Tian Y, Ghausi SA, Zhang Y *et al.* Radiation as the dominant cause of high-temperature extremes on the eastern Tibetan Plateau. *Environ Res Lett*. 2023; **18**(7): 074007.
7. Zeppetello LRV, Tétreault-Pinard É, Battisti DS *et al.* Identifying the sources of continental summertime temperature variance using a diagnostic model of land–atmosphere interactions. *J Clim*. 2020; **33**(9): 3547-3564.
8. Lee S, Gong T, Feldstein SB *et al.* Revisiting the cause of the 1989–2009 Arctic surface warming using the surface energy budget: downward infrared radiation dominates the surface fluxes. *Geophys Res Lett*. 2017; **44**(20): 10,654-610,661.
9. Hersbach H, Bell B, Berrisford P *et al.* The ERA5 global reanalysis. *Q J R Meteorol Soc*. 2020; **146**(730): 1999-2049.
10. European Centre for Medium-Range Weather Forecasts. *IFS Documentation-Cy41r2-Part IV: Physical Processes*. 2016 (DHHS publication no.: Report Number)| (GPO o. Document Number)|.
11. Zhou Z, Zhang L, Zhang Q *et al.* Global increase in future compound heat stress-heavy precipitation hazards and associated socio-ecosystem risks. *npj Clim Atmos Sci*. 2024; **7**(1): 33.
12. Wu S, Luo M, Zhao R *et al.* Local mechanisms for global daytime, nighttime, and compound heatwaves. *npj Clim Atmos Sci*. 2023; **6**(1): 36.
13. Lau N-C, Nath M. Model simulation and projection of European heat waves in present-day and future climates. *J Clim*. 2014; **27**(10): 3713-3730.
14. Ju W, Zhou Y. Global Daily GPP Simulation Dataset from 1981 to 2019. 2021.
15. NASA Goddard Space Flight Center. *Daily FluxSat GPP of biomass over Land, Based on MODIS Terra and Aqua adjusted reflectance Collection 6.1, on a Global 0.5 by 0.625 Degree Grid, Level 3*. NASA Goddard Earth Sciences Data and Information Services Center: 2025 (DHHS publication no.: Report Number)| (GPO o. Document Number)|.
16. Chen L, Brun P, Buri P *et al.* Global increase in the occurrence and impact of multiyear droughts. *Science*. 2025; **387**(6731): 278-284.
17. Wang Z, Chen W, Piao J *et al.* Synergistic effects of high atmospheric and soil dryness on record-breaking decreases in vegetation productivity over Southwest China in 2023. *npj Clim Atmos Sci*. 2025; **8**(1): 6.
18. Gu L, Schumacher DL, Fischer EM *et al.* Flash drought impacts on global ecosystems amplified by extreme heat. *Nat Geosci*. 2025.
19. Huang S, Wang S, Wang C *et al.* Soil moisture deficits amplify and prolong vegetation productivity losses under compound dry-hot extremes. *Innov Geosci*. 2026: 100235.

20. Duan H, Wu J, Huang G *et al.* Individual and interactive effects of drought and heat on leaf physiology of seedlings in an economically important crop. *AoB PLANTS*. 2017; **9**(1): plw090.

21. Gampe D, Zscheischler J, Reichstein M *et al.* Increasing impact of warm droughts on northern ecosystem productivity over recent decades. *Nat Clim Change*. 2021; **11**(9): 772-779.

22. Breiman L. Random Forests. *Mach Learn*. 2001; **45**(1): 5-32.

23. Lundberg SM, Erion G, Chen H *et al.* From local explanations to global understanding with explainable AI for trees. *Nat Mach Intell*. 2020; **2**(1): 56-67.

24. Zhou J, Zhang J, Huang Y. Evaluation of soil temperature in CMIP6 multimodel simulations. *Agric For Meteorol*. 2024; **352**: 110039.

25. Soong JL, Phillips CL, Ledna C *et al.* CMIP5 Models Predict Rapid and Deep Soil Warming Over the 21st Century. *J Geophys Res Biogeosci*. 2020; **125**(2): e2019JG005266.

26. Lejeune Q, Davin EL, Gudmundsson L *et al.* Historical deforestation locally increased the intensity of hot days in northern mid-latitudes. *Nat Clim Change*. 2018; **8**(5): 386-390.

27. Yue S, Wang C. The Mann-Kendall test modified by effective sample size to detect trend in serially correlated hydrological series. *Water Resour Manag*. 2004; **18**(3): 201-218.

28. Zomer RJ, Xu J, Trabucco A. Version 3 of the Global Aridity Index and Potential Evapotranspiration Database. *Sci Data*. 2022; **9**(1): 409.

29. Dinerstein E, Olson D, Joshi A *et al.* An Ecoregion-Based Approach to Protecting Half the Terrestrial Realm. *BioScience*. 2017; **67**(6): 534-545.

30. Li B, Ryu Y, Jiang C *et al.* BESSv2.0: A satellite-based and coupled-process model for quantifying long-term global land–atmosphere fluxes. *Remote Sens Environ*. 2023; **295**: 113696.

31. Chen JM, Ju W, Ciais P *et al.* Vegetation structural change since 1981 significantly enhanced the terrestrial carbon sink. *Nat Commun*. 2019; **10**(1): 4259.

32. Joiner J, Yoshida Y, Zhang Y *et al.* Estimation of terrestrial global gross primary production (GPP) with satellite data-driven models and eddy covariance flux data. *Remote Sens*. 2018; **10**(9): 1346.
